# Comparative effects of exercise modalities on visceral adipose tissue in children and adolescents with overweight or obesity: a systematic review and three-level meta-analysis

**DOI:** 10.3389/fpubh.2026.1922842

**Published:** 2026-10-22

**Authors:** Chuan Chen, Wei Zhang, Kaihua Liu, Xianming Ding, Peng Tong

**Affiliations:** 1 Jishou University, Jishou, China; 2 Zhangjiajie People’s Hospital, Zhangjiajie, China; 3 Medical School of Jishou University, Jishou, China

**Keywords:** body composition, cardiorespiratory fitness, exercise intervention, pediatric obesity, three-level meta-analysis, visceral adipose tissue

## Abstract

**Objective:**

Previous syntheses included broader age ranges and heterogeneous exercise classifications, leaving uncertainty about comparative effects across exercise modalities in pediatric populations. This review examined whether exercise modalities differ in their effects on visceral adipose tissue (VAT) and waist circumference (WC) in children and adolescents with overweight or obesity, and whether any modality shows superior effectiveness.

**Methods:**

Randomized and partially randomized controlled trials were identified from PubMed, Embase, the Cochrane Library, Web of Science, SPORTDiscus, and CNKI. VAT and WC were primary outcomes; SAT, body fat percentage, BMI, body weight, fat mass, and VO₂max were secondary outcomes. Hedges’ g was synthesized using three-level random-effects models to accommodate dependent effect sizes within studies. Risk of bias was assessed with the Cochrane tool.

**Results:**

Twenty-one trials involving 1,754 participants were included. Exercise interventions were associated with lower VAT (g = −0.725, 95% CI [−1.120, −0.330], *p* < 0.001) and WC (g = −0.692, 95% CI [−0.984, −0.400], *p* < 0.001). HIIT showed the largest VAT point estimate (g = −1.332, 95% CI [−1.874, −0.789]), but between-modality differences were not significant for VAT (*p* = 0.116) or WC (*p* = 0.184). Prediction intervals for both primary outcomes crossed the null, indicating uncertainty across study settings. Secondary outcomes generally favored exercise.

**Conclusion:**

Exercise-based interventions were associated with reductions in VAT and WC, but current evidence does not establish the superiority of any single modality. Although HIIT showed the largest VAT point estimate, non-significant between-modality tests and prediction intervals crossing the null preclude firm conclusions about an optimal exercise modality.

**Systematic review registration:**

CRD420261431627, https://www.crd.york.ac.uk/PROSPERO/view/CRD420261431627.

## 1 Introduction

Childhood and adolescent overweight and obesity have become major public health challenges in global efforts to prevent non-communicable diseases. According to the World Health Organization, more than 390 million children and adolescents aged 5–19 years were living with overweight in 2022, including more than 160 million with obesity; the prevalence of overweight or obesity in this age group increased from 8% in 1990 to 20% in 2022 (1). The UNICEF 2025 Child Nutrition Report further showed that, for the first time, the global prevalence of obesity among children and adolescents aged 5–19 years exceeded that of underweight, indicating a shift in the burden of childhood malnutrition from traditional undernutrition towards a new pattern characterized by the coexistence of undernutrition and overnutrition, and by the rapid rise of obesity (2). This trend has important life-course implications, because obesity during childhood and adolescence can track into adulthood and is associated with an increased risk of cardiometabolic disease later in life (3, 4).

Importantly, cardiometabolic risk associated with obesity is determined not only by the total amount of adiposity but also by its regional distribution. Visceral adipose tissue (VAT), which accumulates within the abdominal cavity around internal organs, and ectopic fat, referring to lipid deposition in non-adipose tissues such as the liver and skeletal muscle, are particularly relevant to metabolically adverse phenotypes (5, 6). In children and adolescents, excessive VAT accumulation has been associated with insulin resistance, dyslipidaemia, elevated blood pressure, and other cardiometabolic abnormalities (7, 8). Importantly, these associations are not fully explained by general measures of body size or total adiposity. For example, VAT remained associated with several cardiometabolic risk factors after adjustment for total body fat in a cohort of 472 youth aged 6–18 years (9), and a study of 337 adolescent girls reported associations of VAT with triglycerides and insulin resistance after accounting for body mass index (BMI) percentile (10). These findings indicate that VAT can provide cardiometabolic risk information beyond that conveyed by BMI alone, which cannot directly characterize regional adipose tissue distribution. Accordingly, evaluating exercise-induced changes in VAT may offer a more specific assessment of changes in metabolically relevant adiposity than relying solely on changes in body weight or BMI.

Exercise modalities differ in intensity, metabolic demand, muscle recruitment, and training structure, and may therefore influence visceral adiposity through partly distinct physiological pathways. Sustained aerobic exercise may facilitate lipid mobilization and oxidation through prolonged cardiometabolic loading, whereas resistance training increases skeletal muscle recruitment and may improve metabolic function, including contraction-mediated, insulin-independent glucose uptake by skeletal muscle (11, 12). High-intensity interval training (HIIT) imposes repeated high-intensity metabolic and cardiopulmonary stimuli, while combined aerobic and resistance training integrates endurance- and strength-oriented adaptations (13–15). However, whether these physiological differences translate into meaningful differences in VAT reduction remains uncertain. Randomized trials in children and adolescents with obesity have reported varying modality-specific patterns: aerobic training reduced VAT whereas resistance training did not in one trial (16), combined aerobic and resistance training produced greater improvements in visceral adiposity than aerobic training alone in another (17), whereas other studies reported broadly comparable body-composition and metabolic responses across exercise approaches or improvements in fitness without parallel reductions in visceral adiposity (18, 19). Thus, existing evidence provides a biological rationale for comparing exercise modalities but does not establish the consistent superiority of any single modality for reducing VAT. Cardiorespiratory fitness was retained as a complementary secondary outcome because it represents an important marker of health in youth and captures a functional adaptation to exercise that may occur independently of changes in VAT or other adiposity measures (20).

Previous evidence syntheses have shown that exercise can reduce visceral or abdominal adiposity in children and adolescents with overweight or obesity (21, 22), indicating that the central question is no longer simply whether exercise is effective. Rather, an unresolved issue is whether specific exercise modalities produce meaningfully different reductions in visceral and central adiposity. A previous meta-analysis of randomized controlled trials involving individuals aged 6–24 years reported reductions in visceral fat with aerobic exercise and HIIT, but not with resistance or combined exercise, and suggested that HIIT might produce a greater reduction than aerobic exercise (23). However, that analysis combined pediatric and young-adult populations in its modality-specific estimates, and a formal overall test of between-modality differences was not reported (23). Importantly, a larger pooled effect observed within one exercise modality cannot be interpreted as evidence of superiority unless differences between modalities are formally tested. As the pediatric evidence base has expanded, an updated comparative synthesis is therefore needed to determine whether the apparent differences among exercise modalities are statistically supported specifically in children and adolescents. Such comparative evidence is also important for informing modality-specific exercise prescription in pediatric obesity.

A further methodological challenge is that many eligible trials contribute multiple correlated effect sizes through multi-arm designs, multiple intervention comparisons, or multiple eligible measurements. Treating these estimates as statistically independent may underestimate uncertainty, whereas selecting only one effect size per study can discard relevant information. A three-level meta-analytic framework allows multiple eligible effect sizes to be retained while accommodating their nested within-study structure and separating sampling error, within-study variability, and between-study heterogeneity. Accordingly, this systematic review and three-level meta-analysis aimed to determine whether the effects on visceral adipose tissue (VAT) and waist circumference (WC) differ across exercise modalities in children and adolescents with overweight or obesity, and whether current evidence supports the superiority of any specific modality. SAT, body fat percentage, BMI, body weight, fat mass, and VO₂max were evaluated as secondary outcomes to provide complementary information on body composition and cardiorespiratory fitness.

## 2 Methods

This systematic review and meta-analysis was designed, conducted, and reported in accordance with the PRISMA 2020 statement (24). Where applicable, the PRISMA 2020 Explanation and Elaboration document was also consulted to enhance the completeness and transparency of reporting (25). The review protocol was registered with PROSPERO on 6 June 2026 (registration number: CRD420261431627). The database searches had been completed before registration; however, formal eligibility screening of the retrieved records had not yet commenced, and registration preceded formal study selection, data extraction, and quantitative synthesis. Protocol registration was used to enhance the transparency, reproducibility, and reporting rigor of the review process (26, 27).

### 2.1 Search strategy and study selection

#### 2.1.1 Data sources and searches

To systematically identify randomized controlled trials examining the effects of different exercise modalities on visceral adipose tissue (VAT) in children, adolescents, and youth with overweight or obesity, we searched PubMed, the Cochrane Library, Embase, Web of Science, SPORTDiscus via EBSCOhost, and China National Knowledge Infrastructure (CNKI). Searches covered each database from inception to 20 April 2026. An updated search was conducted on 16 May 2026 to capture the most recent available evidence. These searches were completed before PROSPERO registration; formal eligibility screening of the retrieved records commenced thereafter. Eligible studies were restricted to those published in Chinese or English because these were the languages in which the review team could independently assess full-text eligibility and verify extracted data without reliance on translation. This restriction was applied to maintain consistency and accuracy in study selection and data extraction. The search strategy was developed according to the PICOS framework and included five core concepts: children, adolescents, or youth; overweight or obesity; exercise or physical activity interventions; VAT-related outcomes; and randomized controlled trials (28, 29). Search terms were adapted for each database using controlled vocabulary, Emtree terms, MeSH terms, free-text terms, and database-specific field tags where applicable. Within each concept block, terms were combined using “OR”; different concept blocks were combined using “AND.” The complete search strategies for all databases were prepared in line with the reporting principles of PRISMA-S and are provided in Supplementary Method S1 (28). To reduce the risk of missing potentially eligible studies, we also manually screened the reference lists of included studies and relevant systematic reviews.

#### 2.1.2 Study selection and eligibility criteria

All retrieved records were imported into EndNote X9 for reference management. Duplicate records were removed using a combination of the software’s automatic duplicate-detection function and manual verification. Two reviewers (CC and XD) independently screened titles and abstracts according to the prespecified inclusion and exclusion criteria. Full texts were obtained and independently assessed for all records considered potentially eligible. Disagreements during the selection process were first resolved through discussion; unresolved disagreements were adjudicated by a third reviewer (KL). The processes of study identification, deduplication, title and abstract screening, full-text assessment, and final inclusion were summarized using a PRISMA flow diagram (24, 25).

Eligible studies were selected according to the following criteria.

*(1) Population*. Eligible participants were children and adolescents with overweight or obesity, as defined in the original studies using established age-appropriate criteria, including BMI, BMI z-score, BMI percentile, or other recognized age- and sex-specific standards (30, 31). The review primarily targeted pediatric and adolescent populations. Studies explicitly characterized by the original investigators as adolescent or youth populations were not automatically excluded when the reported age distribution extended marginally beyond the conventional adolescent age boundary, provided that the sample remained substantively consistent with the target developmental population. Studies including broader populations were eligible only when data corresponding to the target population could be extracted separately or when the reported sample as a whole met the above operational population criterion. No restrictions were imposed on sex, country, or region.

*(2) Intervention*. Eligible interventions were those primarily involving exercise training, physical activity promotion, or structured exercise programmes. Exercise interventions were classified *a priori* according to training modality, intensity, and training structure, based on previously published exercise-classification frameworks (32–34). Classification was determined from the actual intervention characteristics reported in the original trials rather than solely from the terminology used by the study authors. Seven categories were defined: moderate-intensity aerobic exercise (AE[M]), moderate-to-vigorous-intensity aerobic exercise (AE[MV]), vigorous-intensity aerobic exercise (AE[V]), resistance training (RT), aerobic exercise combined with resistance training (AE + RT), high-intensity interval training (HIIT), and high-intensity interval training combined with resistance training (HIIT+RT). For aerobic exercise, moderate intensity was defined as approximately 45–65% VO₂max, 50–65% heart rate reserve (HRR), or 65–75% maximum heart rate (MHR), whereas vigorous intensity was defined as >65% VO₂max, >65% HRR, or >75% MHR. Interventions spanning both moderate and vigorous intensity ranges that could not be separated into distinct effects were classified as AE[MV]. Resistance training was defined as structured resistance exercise, generally performed at ≥50% one-repetition maximum (1RM) when intensity was reported. HIIT was characterized by repeated bouts of vigorous or near-maximal exercise interspersed with recovery periods, including sprint interval protocols where applicable. Combined interventions were classified as AE + RT or HIIT+RT according to whether the aerobic component consisted predominantly of conventional aerobic training or high-intensity interval training, respectively. When exercise training was delivered together with dietary advice, health education, behavioral counselling, or other lifestyle components, studies were eligible when structured exercise remained a clearly identifiable component of the intervention and the comparison permitted a meaningful evaluation of the exercise modality, intensity, or training structure under investigation. The presence and nature of accompanying non-exercise components were extracted and considered when interpreting the resulting effect estimates. Full operational definitions and decision rules are provided in Supplementary Method S2.

*(3) Comparator*. Eligible comparator conditions included no additional structured exercise, wait-list control, usual lifestyle, usual care, routine school physical education, health education, nutritional or lifestyle advice, attention control, light stretching, delayed intervention, minimal-exercise conditions, or another eligible structured exercise intervention. Because the review addressed both the effects of exercise-based interventions relative to non-exercise or minimal-exercise comparators and the comparative effects of different exercise modalities, trials directly comparing two or more eligible exercise interventions were also eligible. For studies involving dietary, educational, behavioral, or other lifestyle components, the comparison was required to permit a meaningful evaluation of the exercise modality, intensity, or training structure under investigation. The nature of additional non-exercise components was recorded and considered when interpreting the resulting effect estimates.

*(4) Outcomes*. Studies were required to report at least one VAT or equivalent visceral fat-related outcome and to provide sufficient data for effect-size calculation. VAT and WC were designated as the primary outcomes; reporting of VAT or an equivalent visceral fat indicator was required for study eligibility, whereas WC was analyzed when available among otherwise eligible studies. Secondary outcomes included subcutaneous adipose tissue (SAT), body fat percentage, BMI or BMI z-score, body weight, fat mass, and VO₂max, VO₂peak, or other comparable cardiorespiratory fitness indicators. The operational definitions, measurement rules, and effect directions for the primary and secondary outcomes are provided in Supplementary Table S2.

*(5) Study design*. Eligible studies were randomized controlled trials or partially randomized controlled trials with prospective concurrent comparison groups. Eligible designs included individually randomized, parallel-group, multi-arm, cluster-randomized, pilot, and feasibility trials. Partially randomized trials were eligible when the original study prospectively assigned participants to concurrent intervention and comparator conditions and otherwise satisfied the population, intervention, comparator, and outcome criteria of the review. Studies in which the randomization procedure was incompletely reported were retained only when the original report clearly described prospective allocation to concurrent comparison groups and provided sufficient VAT-related data for the eligible comparison. Crossover trials were eligible only when independent first-period data could be extracted or when sufficient information was available to minimize the potential influence of carryover effects.

If multiple reports were available for the same trial, they were treated as a single study (29). The report providing the most complete information relevant to this review was used as the primary data source, while companion publications were used to supplement missing or additional information where necessary.

Studies were excluded if they did not meet the prespecified eligibility criteria, lacked sufficient VAT-related data for quantitative synthesis after all available sources had been examined, or represented duplicate or overlapping reports of an already included trial.

### 2.2 Data extraction and quality assessment

#### 2.2.1 Data extraction

Two reviewers (CC and XD) independently extracted data using a prespecified data extraction form. Extracted information included basic study characteristics, study design, participant characteristics (including sample size, age, and sex distribution where reported), criteria used to define overweight or obesity, intervention and comparator conditions, exercise modality, exercise dose, intervention duration, outcomes, measurement methods, measurement time points, and data required for effect-size calculation. Participant characteristics were extracted at the intervention-arm level when these data were reported separately in the original publication. When only whole-sample characteristics were available, the study-level values were retained as reported and identified accordingly rather than being treated as arm-specific estimates. Information not reported in the original study was recorded as not reported (NR). Detailed characteristics of the included studies are summarized in Supplementary Table S1, and outcome definitions and measurement rules are provided in Supplementary Table S2. The assessment closest to the end of the intervention was selected as the primary time point for quantitative synthesis. Effect-size calculations were based on change-from-baseline scores from baseline to the immediate post-intervention assessment. When change scores and their corresponding standard deviations were reported directly, these values were extracted. When change scores were not reported but sufficient baseline and post-intervention data were available, change scores were derived from the reported data using established methods. Post-intervention values alone were not combined with change-score effect sizes in the primary meta-analysis. For incompletely reported data, relevant information was sought from the main text, tables, Supplementary materials, figures, and trial registration records, and the original authors were contacted when necessary. If the information required to obtain or derive change-from-baseline data remained unavailable, the corresponding outcome was excluded from the quantitative synthesis but retained for narrative description where appropriate. Disagreements during data extraction were resolved through discussion; unresolved disagreements were adjudicated by a third reviewer (KL).

#### 2.2.2 Risk of bias assessment

Two reviewers independently assessed the risk of bias of the included controlled trials using the Cochrane risk-of-bias tool (35). The assessed domains included random sequence generation, allocation concealment, blinding of outcome assessment, incomplete outcome data, selective reporting, and other potential sources of bias. Each domain was judged as low, unclear, or high risk (36). The overall risk-of-bias judgment was classified as high when at least one domain was rated as high risk, low when all assessed domains were rated as low risk, and unclear otherwise. Disagreements were resolved through discussion and, when necessary, by adjudication from a third reviewer. Risk-of-bias assessments were used to inform the interpretation of evidence robustness and certainty of evidence, rather than serving as exclusion criteria for the primary quantitative synthesis (37). Detailed study-level and domain-level assessments are summarized in Supplementary Table S3 and Supplementary Figure S1.

### 2.3 Meta-analytic procedures

#### 2.3.1 Effect-size calculation

The small-sample-corrected standardized mean difference, Hedges’ g, was used as the effect-size metric for the primary and secondary outcomes. A standardized effect-size metric was selected because several outcome domains were reported using non-equivalent scales or units. In particular, VAT was quantified as area, volume, mass, or other regional visceral fat indices using different measurement approaches; similar scale heterogeneity was present for SAT, BMI-related outcomes, and cardiorespiratory fitness measures. Using Hedges’ g therefore allowed these measures to be expressed on a common standardized scale and enabled a consistent analytical framework across outcome domains (38, 39). Effect sizes were calculated from change-from-baseline scores for the intervention and comparator groups from baseline to the assessment closest to the end of the intervention. When mean change scores and their corresponding standard deviations were reported directly, these values were used. When mean change scores were not reported but baseline and post-intervention means were available, the mean change was derived from the reported values. When the standard deviation of the change score was not reported but baseline and post-intervention standard deviations were available, it was estimated as follows:

$$\begin{array}{l} {\mathrm{SD}}_{\text{change}}=\surd ({\mathrm{SD}}_{{\text{baseline}}^{2}}+{\mathrm{SD}}_{{\text{post}}^{2}}\\ -2r\times {\mathrm{SD}}_{\text{baseline}}\times {\mathrm{SD}}_{\text{post}}) \end{array}$$

Here, r represents the correlation between baseline and post-intervention measurements. When this correlation was not reported or could not be derived from the original study, a correlation coefficient of r = 0.50 was assumed consistently. Post-intervention values alone were not combined with change-score effect sizes in the primary meta-analysis. When outcomes were reported as standard errors, confidence intervals, or other convertible statistics, these values were converted to standard deviations using established formulae before effect-size calculation. Additional procedures for data conversion and estimation of missing standard deviations are provided in Supplementary Method S3. Effect directions were harmonized so that, for adiposity-related outcomes, negative values indicated lower values or greater reductions in the intervention arm relative to the comparator arm, whereas for cardiorespiratory fitness outcomes, positive values indicated greater improvement in the intervention arm relative to the comparator arm. Standardized effect sizes were interpreted with reference to conventional benchmarks of <0.20 as trivial, 0.20–0.50 as small, 0.50–0.80 as moderate, and >0.80 as large; these thresholds were used only as general interpretative guides (40).

#### 2.3.2 Data processing and analysis

Basic characteristics of the included studies were summarized using frequencies, percentages, means, standard deviations, medians, or interquartile ranges, as appropriate. Because some studies used multi-arm randomized designs or reported multiple eligible outcomes, a single study could contribute more than one effect size. For multi-arm trials, eligible comparisons sharing a common control group were retained as separate effect sizes and nested within the same study. To account for the dependency of effect sizes within studies, a three-level random-effects model was used as the primary analytic framework (38, 41). This model decomposed the total variability in effect sizes into sampling error at Level 1, variance among effect sizes within the same study at Level 2, and variance between studies at Level 3 (38). The main models estimated variance components using restricted maximum likelihood (REML), with effect sizes nested within studies (42). Because study-level nesting does not by itself explicitly model the sampling covariance induced by repeated use of a shared comparator group, and because the exact sampling covariances required to specify this dependence were not consistently available from the original studies, these covariance terms were not explicitly incorporated into the sampling variance–covariance structure. Robust variance estimation (RVE), clustered at the study level with a small-sample correction, was therefore additionally used to assess the robustness of statistical inference to residual within-study dependence, including dependence arising from shared comparator groups (41). A separate shared-comparator sensitivity analysis was also conducted as described below. Relevant statistical terms and the interpretative framework for the three-level model are provided in Supplementary Method S4. For each outcome, the pooled effect estimate, 95% confidence interval (CI), and 95% prediction interval (PI) were calculated (43). To evaluate the necessity of the three-level structure, nested models were fitted using maximum likelihood and compared between two-level and three-level specifications using likelihood ratio tests, with Akaike information criterion (AIC) and Bayesian information criterion (BIC) also considered. Heterogeneity was assessed using Cochran’s Q test, estimated variance components, and the I^2^ statistic (44). Within the three-level model, total heterogeneity was further decomposed into within-study heterogeneity at Level 2 and between-study heterogeneity at Level 3. To explore potential sources of heterogeneity, prespecified subgroup analyses and meta-regression analyses were conducted.

Exercise category and age group (<12 vs. ≥12 years) were examined as categorical moderators, whereas participants’ mean age and intervention duration were evaluated as continuous moderators using meta-regression. The 12-year cutoff was prespecified as a pragmatic chronological-age boundary rather than a proxy for Tanner stage, consistent with established pediatric age classifications and prior VAT-specific evidence using the same child–adolescent distinction (23, 45). Because pubertal status was not consistently reported across the included trials, this subgroup analysis was interpreted as an age-based comparison, while mean age was additionally modeled continuously to examine potential age-related variation.

All statistical analyses were performed in R version 4.5.3, primarily using the metafor package for three-level random-effects modelling, robust variance estimation, subgroup analyses, and meta-regression (42). Statistical significance was defined as a two-sided *p* value <0.05. Interpretation of the findings considered the magnitude and uncertainty of effect estimates, including 95% CIs, 95% PIs, heterogeneity, RVE results, and sensitivity analyses, rather than statistical significance alone.

#### 2.3.3 Publication bias and small-study effects

Publication bias and small-study effects were assessed using contour-enhanced funnel plots and Egger-type regression tests (46, 47). Egger-type meta-regressions were conducted within the three-level framework using standard error and sampling variance as alternative moderators. Formal statistical tests were not interpreted when the available number of studies or effect sizes was insufficient. The trim-and-fill method was used as a supplementary analysis to estimate potentially missing effect sizes and the corresponding adjusted pooled effect estimate, and fail-safe N was calculated as an additional indicator of robustness to potentially unpublished null findings (48, 49).

#### 2.3.4 Sensitivity analyses

Sensitivity analyses included outlier analysis and leave-one-effect-out analysis within the three-level random-effects framework. Potentially influential outlying effect sizes were identified using model-based diagnostics, after which the models were refitted without the identified outliers and changes in the pooled effect estimate, 95% CI, and heterogeneity were examined (42, 50). Leave-one-effect-out analyses were conducted by sequentially omitting each effect size and refitting the model to evaluate whether the pooled results were disproportionately influenced by any individual effect size. Because exercise modality was a central focus of the review, the exercise-modality moderator analyses were additionally repeated for each outcome after removal of the outcome-specific outlying effect sizes to assess whether the between-modality conclusions were robust to outlier exclusion.

For multi-arm trials in which two or more eligible comparisons shared the same comparator group, an additional shared-comparator sensitivity analysis was performed. The comparator-group sample size was divided as evenly as possible across the relevant pairwise comparisons, while the reported comparator-group mean and standard deviation were retained. Hedges’ g values and corresponding sampling variances were then recalculated, and the three-level models were refitted. This analysis was used to assess whether repeated use of a common comparator materially influenced the pooled estimates or between-modality conclusions.

#### 2.3.5 Certainty of evidence

The certainty of evidence for each outcome was assessed using the Grading of Recommendations Assessment, Development and Evaluation (GRADE) approach. For outcomes for which the evidence base was derived predominantly from randomized controlled trials, the initial certainty was considered high, while design limitations arising from partially randomized trials were explicitly considered within the risk-of-bias domain. Certainty was subsequently downgraded, where appropriate, across five domains: risk of bias, inconsistency, indirectness, imprecision, and publication bias.

Each domain was evaluated separately at the outcome level. Risk-of-bias judgments considered the methodological limitations and relative contribution of the studies informing each pooled estimate. Inconsistency was evaluated using the magnitude and distribution of heterogeneity, including I^2^ and prediction intervals. Indirectness was assessed according to the correspondence of the included populations, interventions, comparators, and outcomes with the review question. Imprecision was evaluated primarily from the width and clinical interpretation of the confidence interval and the amount of information contributing to the estimate. Publication-bias judgments incorporated funnel-plot patterns, Egger-type regression results, trim-and-fill analyses, and other small-study-effect diagnostics where sufficient data were available. Closely related statistical signals were considered jointly to avoid inappropriate double downgrading across domains.

The final certainty of evidence was categorized as high, moderate, low, or very low. Detailed domain-specific judgments and rationales are provided in Supplementary Table S7.

## 3 Results

### 3.1 Literature selection

The study selection process, including reasons for exclusion at the full-text stage, is presented in Figure 1. A total of 894 records were identified through the six electronic databases, with an additional 12 records identified through reference-list screening. Following deduplication, screening, and eligibility assessment, 21 independent studies were included in the systematic review and three-level meta-analysis, contributing 32 effect sizes for the primary VAT outcome.

**Figure 1:**
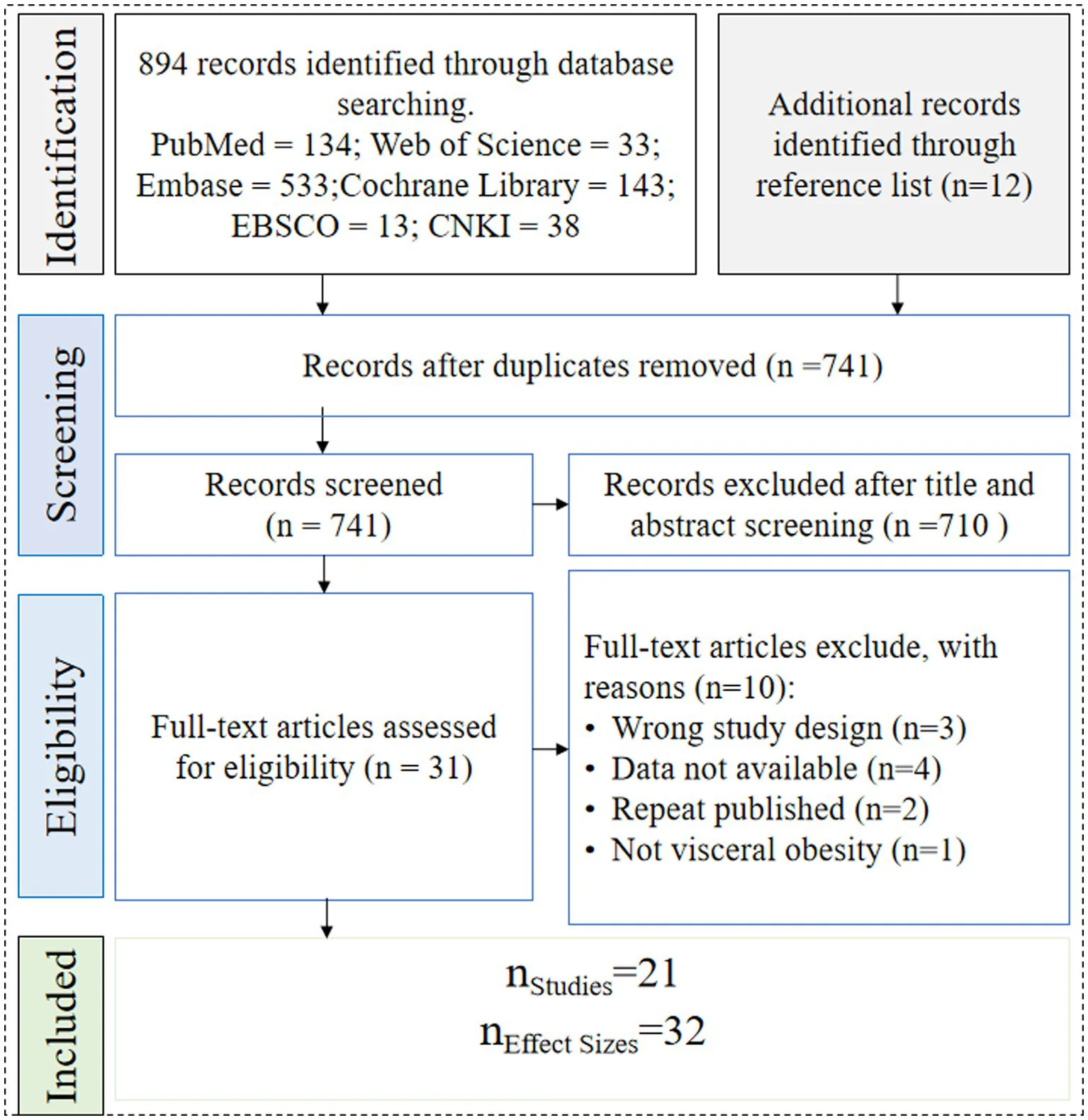
PRISMA flow diagram of the study selection process. PRISMA, Preferred Reporting Items for Systematic Reviews and Meta-Analyses; CNKI, China National Knowledge Infrastructure; VAT, visceral adipose tissue. EBSCOhost SPORTDiscus refers to the platform through which SPORTDiscus was searched. n_studies indicates the number of independent studies included in the systematic review and three-level meta-analysis; n_effect sizes indicates the number of effect sizes included in the primary VAT synthesis.

### 3.2 Characteristics of the included studies

This systematic review and three-level meta-analysis included 21 studies involving 1,754 participants. The included studies were published between 1999 and 2025 and were conducted in the United States (16, 51–58), Brazil (17, 19, 59), Canada (60), China (61–67), and a joint Australia/Norway study (18). All included studies used parallel-group controlled designs; 20 were randomized controlled trials and one was reported as a partially randomized controlled trial. Seven studies used two-arm designs (17, 51, 52, 54, 56, 59, 63), 13 used three-arm designs (16, 18, 19, 53, 55, 57, 58, 61, 62, 64–67), and one used a four-arm design (60). Thus, 14 of the 21 included studies used multi-arm designs with more than two study arms. Sample sizes of individual studies ranged from 29 to 304 participants. Detailed characteristics of the included studies are summarized in Table 1.

**Table 1:** Study characteristics of the included randomized and partially randomized controlled trials.

ID	Study (year)	Country	Design	Group	Sample (M/F)	Age	Diagnosis	Intervention category	Intervention description	Dose/frequency	Duration	Reported outcomes	Measurement
1	Owens 1999 (51)	United States (US)	RCT	Physical training	35 (12/23)	9.5 ± 0.2 SE	Obese children aged 7–11 yr.; triceps skinfold >85th percentile	AE[MV]	Machine-based aerobic exercise plus active games; no dietary intervention	5 d/wk.; 40 min/session; target HR > 150 bpm; mean HR 157 bpm	4 months	VAT; SAAT; TFM; FFM; %BF; physical activity; cardiovascular fitness	MRI: VAT/SAAT volume (cm^3^); DXA: TFM, FFM, %BF; 7-d recall: physical activity; submaximal cycling HR: fitness
1	Owens 1999 (51)	United States (US)	RCT	Control	39 (13/26)	9.4 ± 0.2 SE	Same as above	CON	Non exercising control; no dietary intervention	NR	4 months	Same as above	Same as above
2	Barbeau 2007 (52)	USA	RCT	After-school PA	118 (0/118)	9.5; SD NR	Black girls aged 8–12 yr.; entire adiposity range	AE[V]	After-school PA program including skills instruction, aerobic PA, and toning/stretching	80 min PA/day; 35 min MVPA; HR target >150 bpm; mean HR 160 bpm	10 months	BMI; waist circumference; FM; FFST; %BF; BMC; BMD; VAT; SAAT; CV fitness; PA	MRI: VAT/SAAT volume (cm^3^); DXA: FM, FFST, %BF, BMC, BMD; treadmill test: CV fitness; 7-d recall: PA
2	Barbeau 2007 (52)	USA	RCT	Control	83 (0/83)	9.5; SD NR	Same as above	CON	No intervention	NR	10 months	Same as above	Same as above
3	Davis 2011 (58)	USA	3-arm RCT	CT + MI	12 (0/12)	15.7 ± 1.2	OW/OB Latina girls aged 14–18 yr.; BMI ≥ 85th percentile	AE + RT	Circuit training plus motivational interviewing	2 d/wk.; 60–90 min/session; HR 70–85% HRmax; 4 individual + 4 group MI sessions	16 wk	VO₂max; strength; waist; FM; lean mass; VAT; SAT; hepatic fat; glucose; insulin; HOMA-IR; PA; diet	Treadmill test: VO₂max; 1RM: strength; DXA: FM/lean mass; MRI: VAT/SAT volume (L), hepatic fat; blood tests: glucose/insulin; accelerometer: PA; 3-d diet record
3	Davis 2011 (58)	USA	3-arm RCT	CT	14 (0/14)	15.7 ± 1.1	Same as above	AE + RT	Circuit training including aerobic and strength exercises	2 d/wk.; 60–90 min/session; HR 70–85% HRmax	16 wk	Same as above	Same as above
3	Davis 2011 (58)	USA	3-arm RCT	Control	12 (0/12)	15.8 ± 1.0	Same as above	CON	No intervention during study period; delayed brief CT offered after post-test	NR	16 wk	Same as above	Same as above
4	Saelens 2011 (54)	USA	Pilot RCT	STANDARD	15 (7/8)	10.1 ± 1.1	Overweight children; BMI > 85th percentile; parent BMI ≥ 25	CON	Family-based behavioral weight management mainly targeting diet; general PA advice	14 weekly visits; PA advice ≥60 min/d on most days	14 wk	BMI; BMI z-score; % overweight; whole-body fat; %BF; lean mass; waist; total abdominal fat; VAT; SAT; PA; diet	DXA: fat/lean mass; MRI: abdominal fat, VAT/SAT volume (cm^3^); accelerometer: PA; 3-d food logs: diet
4	Saelens 2011 (54)	USA	Pilot RCT	ADDED	14 (6/8)	9.8 ± 1.2	Same as above	AE[MV]	Same dietary program plus targeted MVPA increase using pedometers, goals, rewards, and PA strategies	14 weekly visits; target ≥90 min/d MVPA on ≥6 d/wk	14 wk	Same as above	Same as above
5	Davis 2012 (53)	USA	3-arm RCT	Low-dose exercise	71 (31/40)	9.3 ± 0.9	OW/OB sedentary children aged 7–11 yr.; BMI ≥ 85th percentile	AE[V]	Aerobic games, jump rope, modified basketball/soccer	20 min/d; 5 d/wk.; mean HR 166 bpm; 7.5 METs; attendance 85%	13 wk	Insulin AUC; glucose; insulin; %BF; VAT; SAT; BMI z-score; peak VO₂	OGTT: insulin/glucose; DXA: %BF; MRI: VAT/SAT volume (cm^3^); treadmill: peak VO₂
5	Davis 2012 (53)	USA	3-arm RCT	High-dose exercise	73 (33/40)	9.4 ± 1.2	Same as above	AE[V]	Same aerobic activities; two 20-min bouts/day	40 min/d; 5 d/wk.; mean HR 165 bpm; 7.5 METs; attendance 84%	13 wk	Same as above	Same as above
5	Davis 2012 (53)	USA	3-arm RCT	Control	78 (30/48)	9.4 ± 1.1	Same as above	CON	Usual physical activity; monthly lifestyle education offered	NR	13 wk	Same as above	Same as above
6	Lee 2012 (55)	USA	3-arm RCT	Aerobic exercise	16 (16/0)	15.2 ± 1.9	Obese inactive adolescent boys aged 12–18 yr.; BMI ≥ 95th percentile; Tanner III–V	AE[MV]	Treadmill, elliptical, or stationary bike exercise; no caloric restriction	3 d/wk.; 60 min/session; 60–75% VO₂peak	3 mo	WC; total adiposity; total AT; VAT; abdominal SAT; liver fat; IMCL; insulin sensitivity; insulin secretion; VO₂peak	MRI: AT, VAT mass (kg), SAT, skeletal muscle; MRS: liver fat, IMCL; DXA: FFM; clamp: insulin sensitivity/secretion; treadmill: VO₂peak
6	Lee 2012 (55)	USA	3-arm RCT	Resistance exercise	16 (16/0)	14.6 ± 1.5	Same as above	RT	Whole-body resistance training using weight-stack equipment; no caloric restriction	3 d/wk.; 60 min/session; 10 exercises; 8–12 reps; 60% RM then to fatigue	3 mo	Same as above	MRI: AT, VAT, SAT, skeletal muscle; MRS: liver fat, IMCL; DXA: FFM; clamp: insulin sensitivity/secretion; RM test: strength
6	Lee 2012 (55)	USA	3-arm RCT	Control	13 (13/0)	14.8 ± 1.4	Same as above	CON	Maintained usual leisure-time activity; no structured exercise	NR	3 mo	Same as above	Same as above
7	Lee 2013 (16)	USA	3-arm RCT	Aerobic exercise	16 (0/16)	14.6 ± 1.9	Obese inactive girls aged 12–18 yr.; BMI ≥ 95th percentile; Tanner III–V	AE[MV]	Treadmill/elliptical aerobic exercise; no caloric restriction	3 d/wk.; 60 min/session; 60–75% VO₂peak	3 mo	BMI; waist; %BF; total AT; VAT; ASAT; liver fat; IMAT; insulin sensitivity; VO₂peak; strength	MRI: AT, VAT/ASAT area (cm^2^); MRS: liver fat; CT: IMAT; clamp: insulin sensitivity; treadmill: VO₂peak; 1RM: strength
7	Lee 2013 (16)	USA	3-arm RCT	Resistance exercise	16 (0/16)	14.8 ± 1.9	Same as above	RT	Whole-body machine-based resistance training; no caloric restriction	3 d/wk.; 60 min/session; 10 exercises; 8–12 reps; 60% 1RM then to fatigue	3 mo	Same as above	Same as above
7	Lee 2013 (16)	USA	3-arm RCT	Control	12 (0/12)	15.0 ± 2.2	Same as above	CON	No structured physical activity	NR	3 mo	Same as above	Same as above
8	Dâmaso 2014 (17)	BRA	RCT	Aerobic training	55 (NR/NR)	Total: 16.89 ± 1.81	Obese post-pubertal adolescents aged 15–19 yr.; BMI ≥ 95th percentile	AE	Aerobic training plus interdisciplinary therapy	3 d/wk.; 60 min/session; 50–70% VO₂ / VT1	1 yr	BMI; body fat; lean mass; VAT; SAT; glucose; insulin; HOMA-IR; QUICKI; lipids; leptin; adiponectin	BOD POD: body composition; ultrasound: VAT/SAT thickness (cm); blood tests: metabolic and inflammatory markers
8	Dâmaso 2014 (17)	BRA	RCT	Aerobic plus resistance training	61 (NR/NR)	Total: 16.89 ± 1.81	Same as above	AE + RT	30 min aerobic plus 30 min resistance training; interdisciplinary therapy	3 d/wk.; 60 min/session; AT at VT1; RT 3 sets, 6–20 RM	1 yr	Same as above	Same as above
9	Alberga 2015 (60)	CAN	4-arm RCT	Aerobic training	75 (22/53)	15.5 ± 1.4	Inactive post-pubertal adolescents aged 14–18 yr.; OW/OB; BMI ≥ 85th percentile with risk factor or ≥95th percentile	AE[MV]	Aerobic exercise plus dietary counselling	4 d/wk.; 20–40 min/session; treadmill/cycle/elliptical; 70–85% HRmax	22 wk	BMI; waist; %BF; SAT; deep SAT; superficial SAT; VAT; ApoA-1; ApoB; ApoB/ApoA-1; HSCRP	MRI: body fat, VAT area at L4–L5 (cm^2^), SAT; blood tests: ApoA-1, ApoB, HSCRP
9	Alberga 2015 (60)	CAN	4-arm RCT	Resistance training	78 (23/55)	15.9 ± 1.5	Same as above	RT	Whole-body resistance training plus dietary counselling	4 d/wk.; 2–3 sets; 6–15 RM; resistance machines	22 wk	Same as above	Same as above
9	Alberga 2015 (60)	CAN	4-arm RCT	Combined training	75 (22/53)	15.5 ± 1.3	Same as above	AE + RT	Aerobic plus resistance training plus dietary counselling	4 d/wk.; aerobic 20–40 min + resistance 2–3 sets/session	22 wk	Same as above	Same as above
9	Alberga 2015 (60)	CAN	4-arm RCT	Control	76 (24/52)	15.6 ± 1.3	Same as above	CON	Non-exercising control plus dietary counselling	NR	22 wk	Same as above	Same as above
10	Monteiro 2015 (19)	BRA	3-arm partial RCT	Aerobic training	18 (10/8)	11.0 ± 1.0	Obese adolescents aged 11–17 yr.; BMI obesity criteria	AE[V]	Walking/running aerobic training; no dietary intervention	3 d/wk.; 50 min/session; 65–85% VO₂peak	20 wk	%BF; FM; FFM; android fat; waist; IAAT; SAT; hepatic steatosis; lipids; glucose; inflammatory markers	DXA: body composition; ultrasound: IAAT/SAT thickness (cm), hepatic steatosis; blood tests: lipids, glucose, cytokines
10	Monteiro 2015 (19)	BRA	3-arm partial RCT	Concurrent training	14 (9/5)	11.0 ± 1.3	Same as above	AE + RT	Aerobic plus resistance circuit training; no dietary intervention	3 d/wk.; 60 min/session; 30 min AE + 30 min RT; 55–75% RM	20 wk	Same as above	Same as above
10	Monteiro 2015 (19)	BRA	3-arm partial RCT	Control	16 (8/8)	11.0 ± 1.9	Same as above	CON	No exercise intervention; usual lifestyle	NR	20 wk	Same as above	Same as above
11	Staiano 2016 (56)	USA	RCT	Exergaming	22 (0/22)	15.3 ± 1.2	OW/OB post-menarcheal girls aged 14–18 yr.; BMI ≥ 85th percentile	AE[MV]	Group-based dance exergaming using Kinect/Xbox 360; self-selected game and intensity	3 d/wk.; 60 min/session; 36 h total	12 wk	BMI z-score; waist; %BF; regional fat; VAT; SAT; TAT; BMD; BP; lipids; glucose; insulin	Anthropometry: BMI/waist; DXA: body fat/BMD; MRI: VAT/SAT/TAT mass (kg); blood tests: lipids, glucose, insulin; BP measurement
11	Staiano 2016 (56)	USA	RCT	Control	19 (0/19)	16.1 ± 1.4	Same as above	CON	Self-directed care; maintain usual physical activity	NR	12 wk	Same as above	Same as above
12	da Silveira Campos 2017 (59)	BRA	RCT	Aerobic training	39 (NR/NR)	Total: 16.0 ± 1.7	Post-pubertal obese adolescents; BMI > 95th percentile; Tanner stage 5	AE[MV]	Treadmill aerobic training plus interdisciplinary therapy	3 d/wk.; 60 min/session; HR at VT1	1 yr	BMI; fat mass; lean mass; VAT; SAT; glucose; insulin; HOMA-IR; HOMA-AD; QUICKI; adiponectin	BOD POD: body composition; ultrasound: VAT/SAT thickness (cm); blood tests: glucose, insulin, adiponectin
12	da Silveira Campos 2017 (59)	BRA	RCT	Aerobic plus resistance training	63 (NR/NR)	Total: 16.0 ± 1.7	Same as above	AE + RT	30 min aerobic plus 30 min resistance training; interdisciplinary therapy	3 d/wk.; 60 min/session; RT 6–20 MR	1 yr	Same as above	Same as above
13	Dias 2017 (18)	AUS/NOR	3-arm RCT	HIIT	33 (16/17)	12.4 ± 1.9	Children with obesity aged 7–16 yr	HIIT	HIIT plus nutrition advice	3 d/wk.; 4 × 4 min at 85–95% HRmax; recovery 50–70% HRmax	12 wk	VO₂peak; VAT; SAT; TAAT; %BF; FM; FFM; BMI; lipids; glucose; HbA1c; C-peptide; HOMA-IR; PA; diet	Treadmill test: VO₂peak; MRI: VAT/SAT/TAAT volume (cm^3^); DXA/BOD POD: body composition; blood tests: biomarkers; accelerometer: PA; food records: diet
13	Dias 2017 (18)	AUS/NOR	3-arm RCT	MICT	32 (15/17)	11.9 ± 2.4	Same as above	AE[M]	Moderate-intensity continuous training plus nutrition advice	3 d/wk.; 44 min/session; 60–70% HRmax	12 wk	Same as above	Same as above
13	Dias 2017 (18)	AUS/NOR	3-arm RCT	Nutrition	34 (15/19)	11.8 ± 2.4	Same as above	CON	Nutrition advice only	NR	12 wk	Same as above	Same as above
14	Liang 2018 (66)	CHN	RCT	HIIT	9 (9/0)	NR	Male children with simple obesity; actual weight >20% above standard weight	HIIT	Treadmill HIIT with 60-s sprint and active recovery	3 d/wk.; 60 s at 100% maximal speed + 3 min at 50% speed; 3–6 bouts/session	12 wk	Weight; BMI; body fat mass; %BF; VAT area; waist; SBP; DBP; Chemerin	Body composition analyzer: body composition/VAT area (cm^2^); tape: waist; sphygmomanometer: BP; ELISA: Chemerin
14	Liang 2018 (66)	CHN	RCT	MICT	9 (9/0)	NR	Same as above	AE	Continuous treadmill aerobic training	3 d/wk.; 30–60 min/session; 80% HRpeak	12 wk	Same as above	Same as above
15	Lee 2020 (57)	USA	3-arm RCT	Aerobic exercise	38 (13/25)	14.4 ± 1.6	OW/OB inactive adolescents aged 12–17 yr.; BMI > 85th percentile; Tanner II–V	AE[M]	Treadmill/elliptical aerobic exercise; no caloric restriction	3 d/wk.; 40–60 min/session; 50–65% VO₂peak	6 mo	Weight; total/regional SAT; VAT; skeletal muscle; lipids; BP; cfPWV; cIMT	MRI: AT/SAT/VAT/SM (VAT mass, kg); blood tests: lipids; BP measurement; ultrasound: cIMT; Complior: cfPWV
15	Lee 2020 (57)	USA	3-arm RCT	Resistance exercise	40 (15/25)	14.4 ± 1.6	Same as above	RT	Whole-body machine-based resistance training; no caloric restriction	3 d/wk.; ~60 min/session; 8 exercises; 2 sets; 12–15 reps	6 mo	Same as above	Same as above
15	Lee 2020 (57)	USA	3-arm RCT	Combined exercise	40 (14/26)	14.5 ± 1.7	Same as above	AE + RT	Aerobic exercise plus resistance training; no caloric restriction	3 d/wk.; ~60 min/session; 30 min AE + 1 set RT exercises	6 mo	Same as above	Same as above
16	Cao 2022 (67)	CHN	3-arm RCT	HIIT	12 (12/0)	11.4 ± 0.8	Obese boys aged 11–13 yr.; BMI ≥ 95th percentile; Tanner I–III	HIIT	School-based running HIIT; normal PE maintained	3 d/wk.; 2 sets × 8 × 15 s at 90–100% MAS; 15 s recovery at 50% MAS	12 wk	BMI; %BF; FM; FFM; VAT; WC; VO₂peak; SBP; DBP; glucose; insulin; HOMA-IR; lipids	DXA: body composition/VAT mass (g); treadmill GXT: VO₂peak; 20-m SRT: MAS; blood tests: glucose, insulin, lipids
16	Cao 2022 (67)	CHN	3-arm RCT	MICT	11 (11/0)	11.2 ± 0.7	Same as above	AE[M]	School-based continuous running; normal PE maintained	3 d/wk.; 30 min/session; 60–70% MAS	12 wk	Same as above	Same as above
16	Cao 2022 (67)	CHN	3-arm RCT	Control	13 (13/0)	11.0 ± 0.7	Same as above	CON	No exercise training; normal behavior and PE maintained	NR	12 wk	Same as above	Same as above
17	Cao 2022 (63)	CHN	RCT	Intervention group	20 (10/10)	11.2 ± 0.7	Obese children aged 10–13 yr.; BMI ≥ 95th percentile	HIIT	School-based HIIT integrated into PE class	3 d/wk.; 3 sets × 8 × 15 s at 100% MAS; 15 s recovery at 50% MAS	12 wk	BM; BMI; %BF; FM; FFM; VAT; VO₂max; MAS; HRrest; HRmax	BIA: body composition/VAT area (cm^2^); 20-m SRT: MAS/VO₂max; Polar HR monitor: HR
17	Cao 2022 (63)	CHN	RCT	Control group	20 (10/10)	10.9 ± 0.4	Same as above	CON	Regular PE and usual school activity; no additional HIIT	NR	12 wk	Same as above	Same as above
18	Cao 2022 (62)	CHN	RCT	HIIT	20 (10/10)	11.2 ± 0.9	Obese children aged 10–13 yr.; BMI obesity criteria	HIIT	School-based running HIIT; no dietary control	3 d/wk.; 3 sets × 8 × 15 s at 100–120% MAS; 15 s recovery at 50% MAS	12 wk	BMI; %BF; FM; FFM; VAT; WC; VO₂max; BP; glucose; insulin; HOMA-IR; lipids	DXA: body composition/VAT mass (g); 20-m SRT: MAS/VO₂max; blood tests: glucose, insulin, lipids; BP/HR measurement
18	Cao 2022 (62)	CHN	RCT	MICT	20 (10/10)	10.9 ± 0.8	Same as above	AE	School-based continuous running; no dietary control	3 d/wk.; 20–40 min/session; 60–80% MAS	12 wk	Same as above	Same as above
18	Cao 2022 (62)	CHN	RCT	Control	20 (10/10)	10.9 ± 0.9	Same as above	CON	No training control	NR	12 wk	Same as above	Same as above
19	Li 2023 (61)	CHN	3-arm RCT	HIIT	16 (NR/NR)	Total: 11.0 ± 0.8	Obese children aged 10–13 yr.; Chinese school-age OW/OB criteria	HIIT	School-based running HIIT; free diet	3 d/wk.; 15 s at 100–120% MAS + 15 s recovery at 50% MAS; 3 sets × 8 bouts	12 wk	BMI; %BF; FM; FFM; WC; VAT; limb/trunk fat; VO₂max; HR; BP	DXA: body composition/VAT area (cm^2^); 20-m SRT: VO₂max/MAS; Polar: HR; BP monitor: SBP/DBP
19	Li 2023 (61)	CHN	3-arm RCT	MICT	15 (NR/NR)	Total: 11.0 ± 0.8	Same as above	AE	School-based continuous running; free diet	3 d/wk.; 20–40 min/session; 60–80% MAS	12 wk	Same as above	Same as above
19	Li 2023 (61)	CHN	3-arm RCT	Control	16 (NR/NR)	Total: 11.0 ± 0.8	Same as above	CON	No training control; usual school PE/activity maintained	NR	12 wk	Same as above	Same as above
20	Cao, 2024 (64)	CHN	3-arm RCT	HIIT	14 (7/7)	12.4 ± 0.4	Obese adolescents; Chinese school-age OW/OB criteria	HIIT	Running HIIT; affective response assessed during exercise	3 d/wk.; 3 sets × 8 × 15 s at 100% MAS; 15 s recovery at 50% MAS; 18 min/session	12 wk	Weight; BMI; %BF; VAT; VO₂max; FS; FAS; RPE; PACES	DXA: %BF/VAT area (cm^2^); GXT + K5: VO₂max/MAS; FS/FAS/RPE: affective response; PACES: enjoyment
20	Cao, 2024 (64)	CHN	3-arm RCT	MIIT	14 (7/7)	12.1 ± 0.6	Same as above	AE[MV]	Moderate-intensity interval running; affective response assessed during exercise	3 d/wk.; 3 sets × 8 × 15 s at 80% MAS; 15 s recovery at 50% MAS; 18 min/session	12 wk	Same as above	Same as above
20	Cao 2024 (64)	CHN	3-arm RCT	Control	14 (7/7)	12.4 ± 0.5	Same as above	CON	No additional exercise training beyond school PE/activity	NR	12 wk	Same as above	Same as above
21	Gao, 2025 (65)	CHN	3-arm single-blind RCT	CT1: SIT + stair climbing	10 (5/5)	Total: 10.1 ± 0.7	Obese children aged 9–13 yr.; BMI ≥ 95th percentile; Tanner I–III	HIIT	Sprint interval training plus stair-climbing aerobic exercise	2 d/wk.; 10 min SIT + 10 min stair climbing; stair climbing 60–70% HRmax	8 wk	BM; BMI; WC; %BF; FM; LBM; VAT; VO₂max; SBP; DBP; HRR	InBody770: body composition/VAT (g in Supplementary Table S2; Methods describe visceral fat area in cm^2^); tape: WC; 20-m SRT: VO₂max/MAS; BP monitor: BP; Polar: HR
21	Gao, 2025 (65)	CHN	3-arm single-blind RCT	CT2: SIT + core strength training	10 (5/5)	Total: 10.1 ± 0.7	Same as above	HIIT+RT	Sprint interval training plus core strength exercises	2 d/wk.; 10 min SIT + 10 min core strength; strength training 60–70% HRmax	8 wk	Same as above	Same as above
21	Gao, 2025 (65)	CHN	3-arm single-blind RCT	Control	10 (5/5)	Total: 10.1 ± 0.7	Same as above	CON	Regular school PE/activity only; no additional exercise	NR	8 wk	Same as above	Same as above

AE[M], moderate-intensity aerobic exercise; AE[MV], moderate-to-vigorous aerobic exercise; AE[V], vigorous-intensity aerobic exercise; AE + RT, aerobic exercise combined with resistance training; CON, control condition; HIIT, high-intensity interval training; HIIT + RT, high-intensity interval training combined with resistance training; RT, resistance training; VAT, visceral adipose tissue; SAT/SAAT/ASAT, subcutaneous abdominal adipose tissue; BMI, body mass index; %BF, body fat percentage; FM, fat mass; TFM, total fat mass; FFM/FFST, fat-free mass or fat-free soft tissue; BMC, bone mineral content; BMD, bone mineral density; AT, adipose tissue; IMCL, intramyocellular lipid; IMAT, intermuscular adipose tissue; MRI, magnetic resonance imaging; DXA, dual-energy X-ray absorptiometry; CT, computed tomography; MRS, magnetic resonance spectroscopy; OGTT, oral glucose tolerance test; HR, heart rate; HRmax, maximum heart rate; MET, metabolic equivalent; MVPA, moderate-to-vigorous physical activity; NR, not reported; PA, physical activity; RCT, randomized controlled trial. Studies are ordered chronologically by year of publication; studies published in the same year are ordered alphabetically by the first author’s surname. VAT measurement units are reported as presented in the original studies.

Participants were predominantly children and adolescents with overweight or obesity, or met adiposity-related inclusion criteria defined in the original studies. Reported participant ages were generally within the pediatric and adolescent developmental range; in a small number of samples, the reported age distribution extended marginally beyond the conventional adolescent age boundary. These studies were retained in accordance with the operational population criterion described above when the original study population remained consistent with the target adolescent or youth context. Age information was incompletely reported in some studies and was therefore handled cautiously in the age subgroup and meta-regression analyses. Criteria used to define overweight or obesity included BMI percentiles, BMI z-scores, percentage overweight, skinfold thickness, national or regional pediatric overweight and obesity standards, and other study-specific obesity criteria. Some studies focused on specific populations, including sedentary children, postpubertal adolescents, physically inactive adolescents (16, 17, 55, 57, 59, 60, 62, 63, 65, 67), single-sex samples, Black girls (52), Latina girls (58), and schoolchildren with obesity (62, 63, 65, 67).

The included exercise interventions varied substantially in exercise modality, intensity, supervision format, and intervention duration. According to the prespecified classification, interventions were categorized as AE[M], AE[MV], AE[V], RT, AE + RT, HIIT, or HIIT+RT. Six studies combined exercise with an additional non-exercise intervention (17, 18, 54, 58–60), including nutritional or dietary interventions, interdisciplinary therapy, or motivational interviewing. Aerobic interventions included treadmill-, cycling-, or running-based training and structured aerobic games (16–19, 51, 53, 55, 59, 61–64, 66, 67). Dance-based exergaming was examined in one trial (56), while after-school or school-based physical activity and interval-training programmes were implemented in several studies (52, 62, 63, 65, 67). Resistance and combined-training interventions included machine-based whole-body resistance training, circuit training, concurrent aerobic and resistance training, and core-strength exercise combined with interval training (16, 17, 19, 55, 57–60, 65).

Intervention duration ranged from 8 weeks to 1 year, with a median duration of 12 weeks. Seventeen of the 21 included studies (81.0%) implemented interventions lasting between 12 weeks and 6 months (16, 18, 19, 51, 53–58, 60–64, 66, 67), whereas one study lasted 8 weeks (65), one lasted 10 months (52), and two lasted 1 year (17, 59). Exercise frequency was generally 2 to 5 sessions per week, and session duration ranged from brief high-intensity interval protocols to 60–90 min. Comparator conditions included no additional exercise intervention, maintenance of usual physical activity, regular school physical education, nutritional advice, dietary counselling without structured exercise, self-guided care, delayed intervention, and, where applicable, another eligible structured exercise intervention. VAT was assessed using magnetic resonance imaging (16, 18, 51–58, 60), ultrasonography (17, 19, 59), dual-energy X-ray absorptiometry (61, 62, 64, 67), or bioelectrical impedance/body-composition analyzers (64–66). Across studies, VAT was reported as volume, mass, cross-sectional area, or thickness using study-specific units. In addition to VAT, WC was reported in 12 studies. Secondary outcomes included SAT, body fat percentage, BMI or BMI z-score, body weight, fat mass, and VO₂max or VO₂peak.

### 3.3 Risk of bias assessment

The risk of bias of the included studies was assessed using the Cochrane risk-of-bias tool. Using the prespecified overall judgment rule described in the Methods, 5 studies were judged to be at low risk of bias (51, 53, 55, 58, 64), 5 at unclear risk (52, 56, 57, 60, 62), and 11 at high risk (16–19, 54, 59, 61–63, 65–67). At the domain level, high-risk judgments were most frequent for incomplete outcome data and other potential sources of bias, with 7 studies rated as high risk in each of these domains. One study was rated as high risk for random sequence generation and one for allocation concealment, whereas no study was rated as high risk for blinding of outcome assessment or selective reporting. Because the overall classification was conservative, with any high-risk domain resulting in a high overall judgment, an overall high-risk rating did not imply that all domains were judged to be at high risk. The overall distribution of risk-of-bias judgments is shown in Figure 2, and detailed study-level and domain-level assessments are provided in Supplementary Table S3 and Supplementary Figure S1.

**Figure 2:**
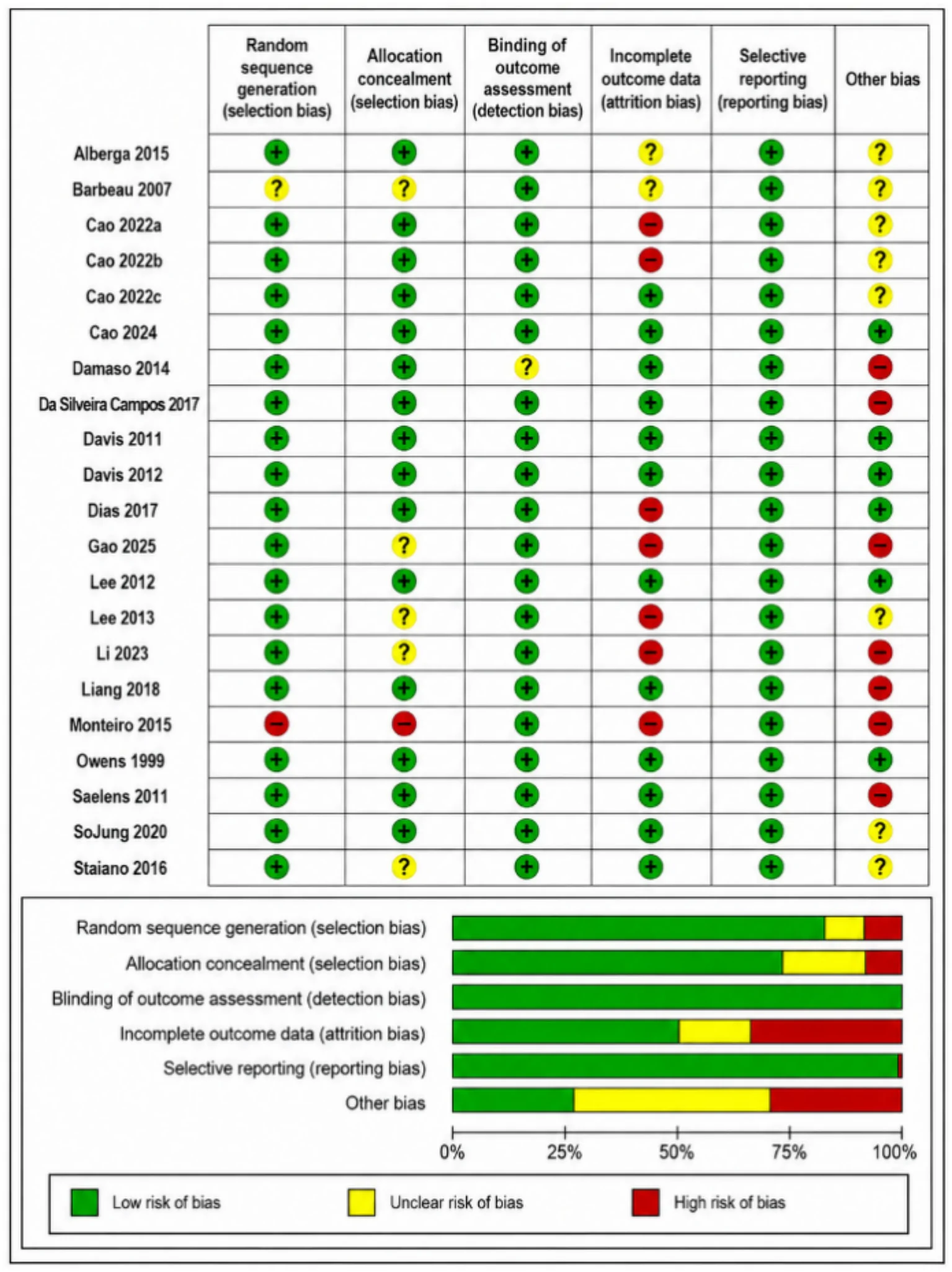
Risk-of-bias assessment of the included studies. Summary of risk-of-bias judgments across domains. Study-level risk-of-bias.

### 3.4 Overall effects of exercise interventions

*Visceral Adipose Tissue*. VAT included 21 studies contributing 32 effect sizes. Across eligible intervention–comparator contrasts, the three-level random-effects model yielded a pooled Hedges’ g of −0.725 (95% CI [−1.120, −0.330], *p* < 0.001), indicating lower VAT or a greater reduction in VAT in the intervention arm relative to the corresponding comparator arm. The 95% prediction interval (PI) [−2.399, 0.948] crossed the null, indicating substantial uncertainty in the true comparative effect across future comparable settings. Heterogeneity was substantial (I^2^ = 89.71%) and was attributable almost entirely to between-study variation. Study-level robust variance estimation yielded a consistent result (Figure 3A; Supplementary Table S4).

**Figure 3:**
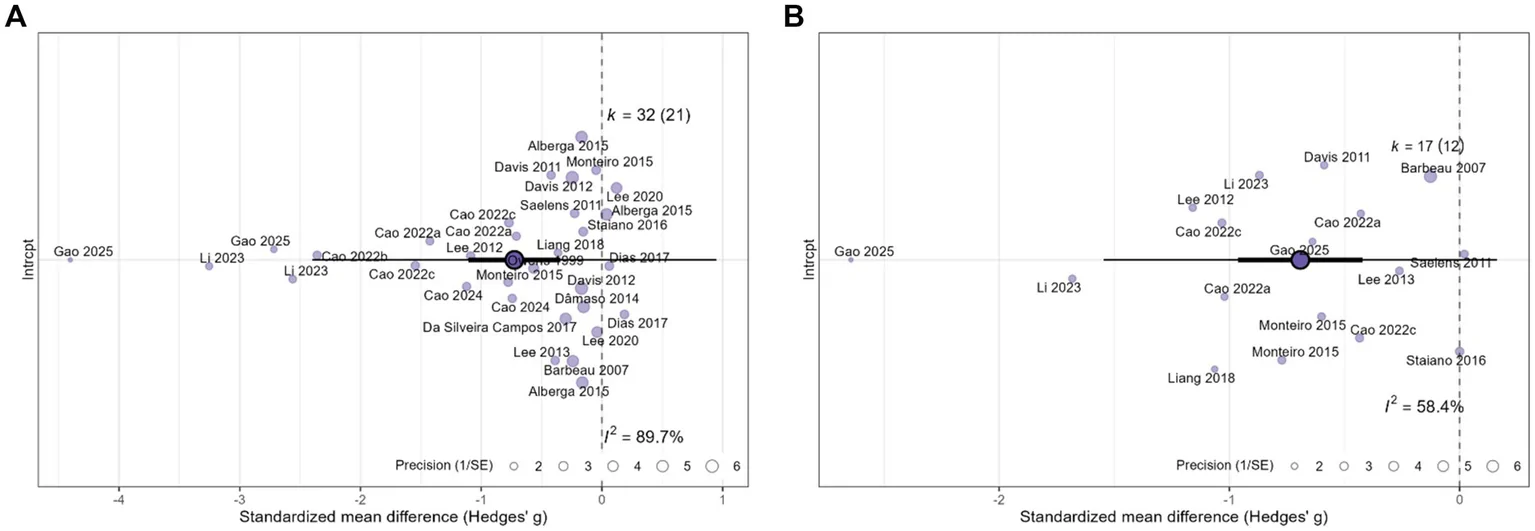
Orchard plots of the pooled effects of exercise interventions on visceral adipose tissue and waist circumference. **(A)** Visceral adipose tissue; **(B)** Waist circumference. Points represent individual effect sizes, with point size reflecting precision. Pooled estimates are shown with 95% confidence intervals and 95% prediction intervals. Negative Hedges’ *g* values favor exercise. *k* denotes the number of effect sizes, with the number of contributing studies shown in parentheses.

*Waist Circumference*. WC included 12 studies contributing 17 effect sizes. The pooled estimate likewise favored the intervention arm (Hedges’ g = −0.692, 95% CI [−0.984, −0.400], *p* < 0.001). The 95% PI [−1.547, 0.163] crossed the null, indicating uncertainty in the true comparative effect across future comparable settings. Heterogeneity was moderate (I^2^ = 58.36%) and was attributable mainly to within-study variation. Study-level robust variance estimation yielded a consistent result (Figure 3B; Supplementary Table S4).

*Other Secondary Outcomes*. Pooled estimates favored the intervention arms for SAT, body fat percentage, BMI, body weight, fat mass, and VO₂max. The corresponding 95% prediction intervals crossed the null for all six outcomes, and heterogeneity was substantial for most outcomes. Full pooled estimates, heterogeneity statistics, prediction intervals, and RVE results are provided in Supplementary Table S4 and Supplementary Figure S2.

### 3.5 Moderator analyses

#### 3.5.1 Subgroup analyses

*Exercise Modality*. For VAT, modality-specific pooled estimates were generally favorable to the intervention arms. Among subgroups represented by multiple effect sizes, HIIT showed the largest point estimate (g = −1.332, 95% CI [−1.874, −0.789], *p* < 0.001), while AE[M] and AE[MV] also showed statistically significant within-subgroup effects. However, the overall between-modality difference was not statistically significant (*p* = 0.116), providing no evidence that any single modality was superior for reducing VAT.

*For waist circumference*, estimates were likewise generally favorable, but the between-modality difference was not significant (*p* = 0.184). Across the secondary outcomes, several modality-specific subgroup effects were statistically significant, but no consistent evidence of between-modality differences was identified. Accordingly, larger or statistically significant within-subgroup estimates should not be interpreted as evidence of modality superiority. VAT and waist-circumference subgroup effects are shown in Figures 4A,B, with complete estimates for all outcomes provided in Supplementary Table S5 and Supplementary Figure S3.

**Figure 4:**
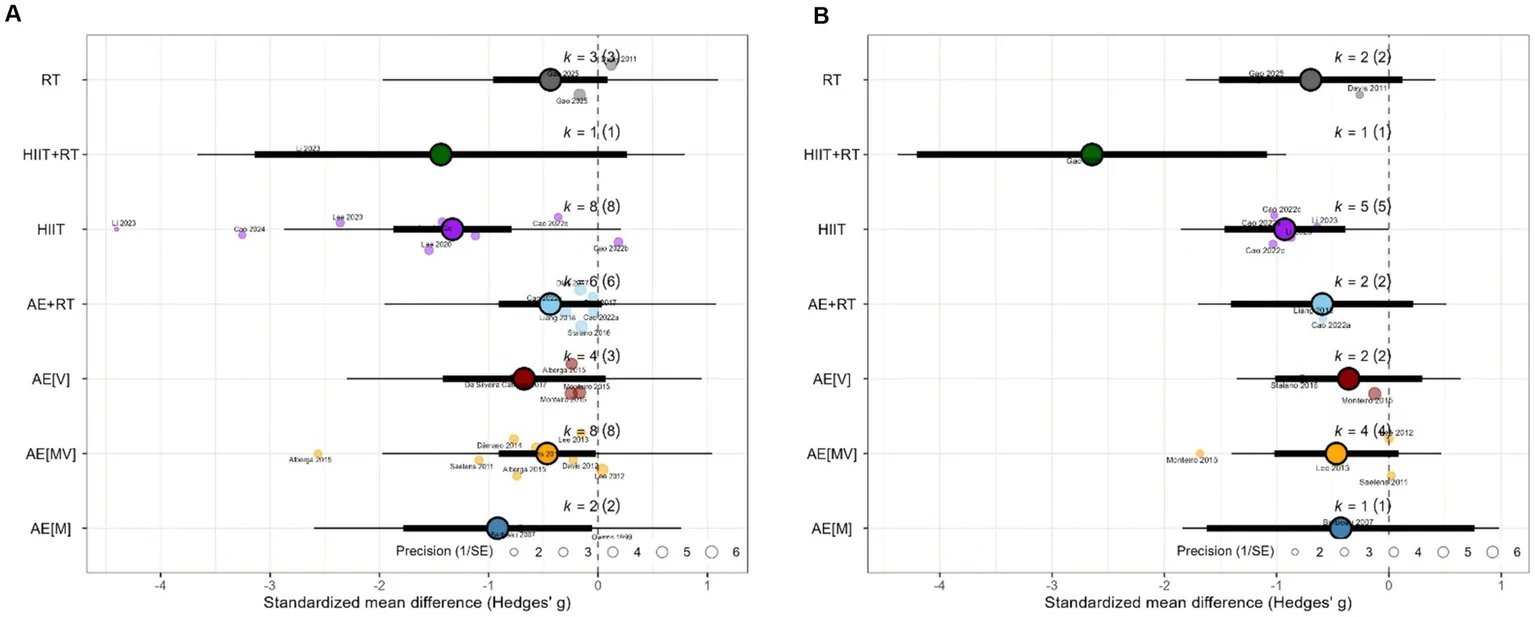
Orchard plots of exercise-modality subgroup effects on visceral adipose tissue and waist circumference. **(A)** Visceral adipose tissue; **(B)** Waist circumference. Points represent pooled subgroup estimates with corresponding 95% confidence and prediction intervals. Negative Hedges’ g values favour exercise. AE[M], moderate-intensity aerobic exercise; AE[MV], moderate-to-vigorous aerobic exercise; AE[V], vigorous-intensity aerobic exercise; AE + RT, aerobic exercise combined with resistance training; HIIT, high-intensity interval training; HIIT+RT, high-intensity interval training combined with resistance training; RT, resistance training.

*Age Group*. For VAT, the pooled effect was numerically larger among participants aged <12 years (g = −1.002, 95% CI [−1.489, −0.515]) than among those aged ≥12 years (g = −0.446, 95% CI [−0.949, 0.057]), but the between-group difference was not statistically significant (*p* = 0.082). No significant age-group difference was observed for waist circumference (*p* = 0.428), and no consistent evidence of age-group moderation was identified across the secondary outcomes. VAT and waist-circumference age-group effects are shown in Figures 5A,B with complete estimates provided in Supplementary Table S5 and Supplementary Figure S3.

**Figure 5:**
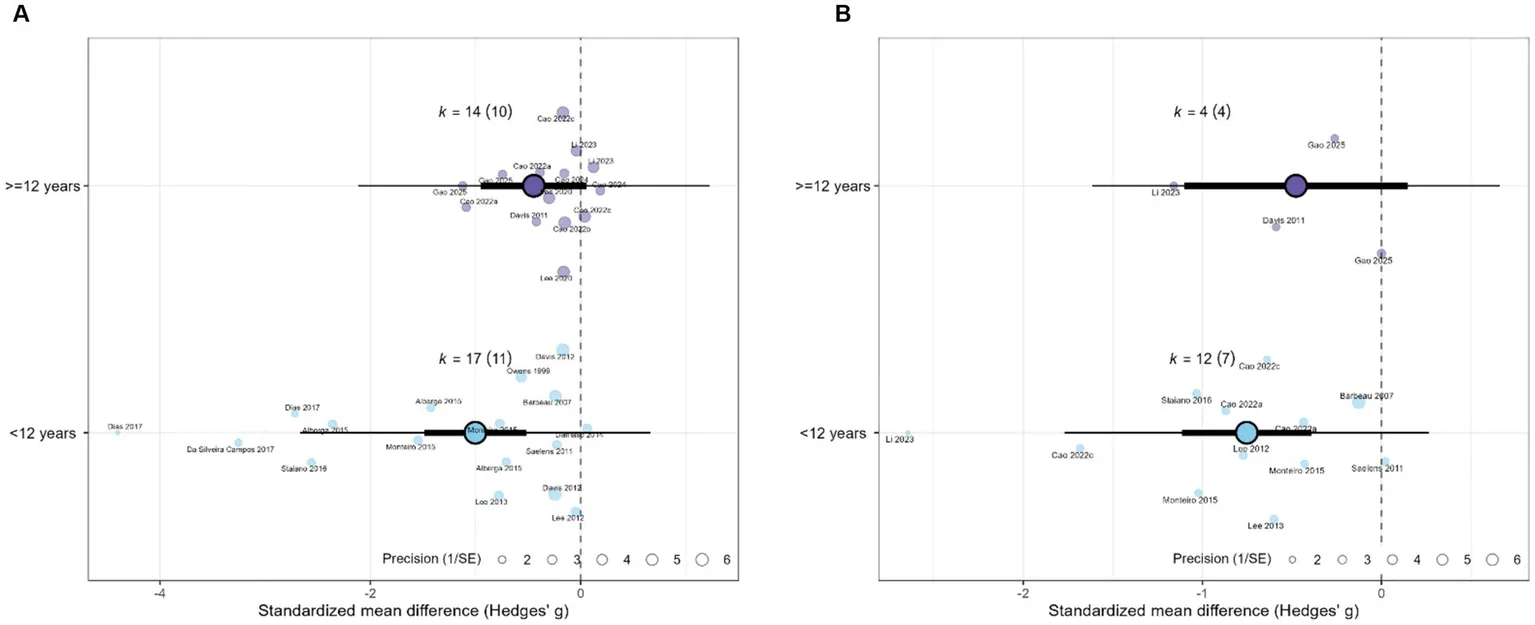
Orchard plots of age-group subgroup effects on visceral adipose tissue and waist circumference. **(A)** Visceral adipose tissue; **(B)** Waist circumference. Effects are presented for participants aged <12 years and ≥12 years. Points represent pooled subgroup estimates with corresponding 95% confidence and prediction intervals. Negative Hedges’ g values favour exercise.

*Overall Risk of Bias*. Exploratory subgroup analyses showed no significant between-category differences according to overall risk of bias for VAT (*p* = 0.372) or waist circumference (*p* = 0.281). No significant differences were identified for body fat percentage, BMI, body weight, fat mass, or VO₂max. SAT was the only outcome showing a nominal between-category difference (*p* = 0.040), with a larger comparative reduction among studies judged to be at low risk of bias. Given the exploratory nature of these analyses, the small number of studies in several risk-of-bias strata, and multiple outcome-level moderator tests, this finding should be interpreted cautiously. Overall, the results did not indicate a consistent systematic shift in pooled comparative effects according to study-level risk of bias. VAT and waist-circumference subgroup effects are shown in Figures 6A,B with complete estimates for all outcomes provided in Supplementary Table S5 and Supplementary Figure S3.

**Figure 6:**
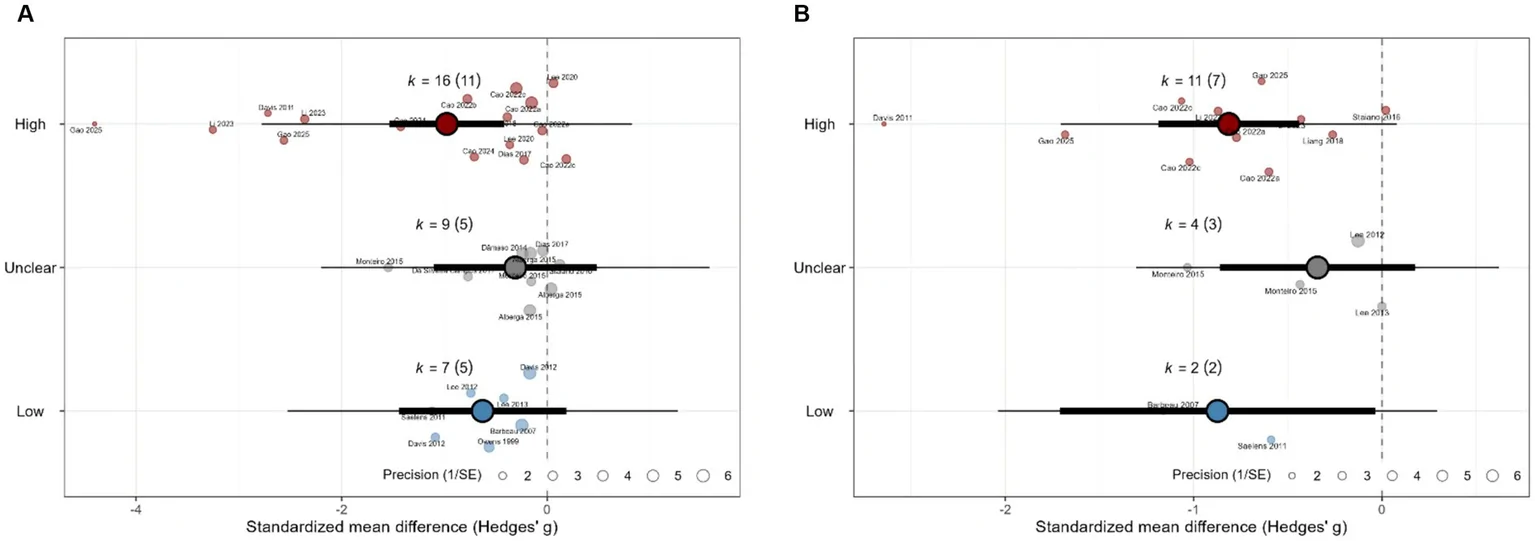
Orchard plots of subgroup effects according to overall risk of bias for visceral adipose tissue and waist circumference. **(A)** Visceral adipose tissue; **(B)** Waist circumference. Studies were classified as having low, unclear, or high overall risk of bias. Points represent pooled subgroup estimates with corresponding 95% confidence and prediction intervals. Negative Hedges’ *g* values favour exercise.

#### 3.5.2 Meta-regression analysis

Continuous-variable meta-regression analyses were conducted to examine whether intervention duration and participants’ mean age explained variation in comparative effect sizes across outcomes.

*Intervention Duration*. Intervention duration was not significantly associated with the comparative effect for VAT (β = 0.025, 95% CI [−0.004, 0.054], *p* = 0.086) or waist circumference (β = 0.026, 95% CI [−0.005, 0.056], *p* = 0.094). No significant associations were identified for any of the secondary outcomes.

*Mean Age*. Mean age was likewise not significantly associated with the comparative effect for VAT (β = 0.105, 95% CI [−0.051, 0.260], *p* = 0.179) or waist circumference (β = 0.037, 95% CI [−0.121, 0.195], *p* = 0.620), and no significant associations were observed across the secondary outcomes.

Overall, neither intervention duration nor mean age was identified as a statistically significant linear moderator for any of the eight outcomes examined. Given the limited number of studies contributing to several outcome-specific models, the restricted range of some moderator values, and substantial residual heterogeneity, these meta-regression findings should be regarded as exploratory. Meta-regression plots for VAT and waist circumference are presented in Figure 7, with complete regression coefficients, standard errors, 95% confidence intervals, and *p* values for all outcomes provided in Supplementary Table S6 and corresponding plots in Supplementary Figure S4.

**Figure 7:**
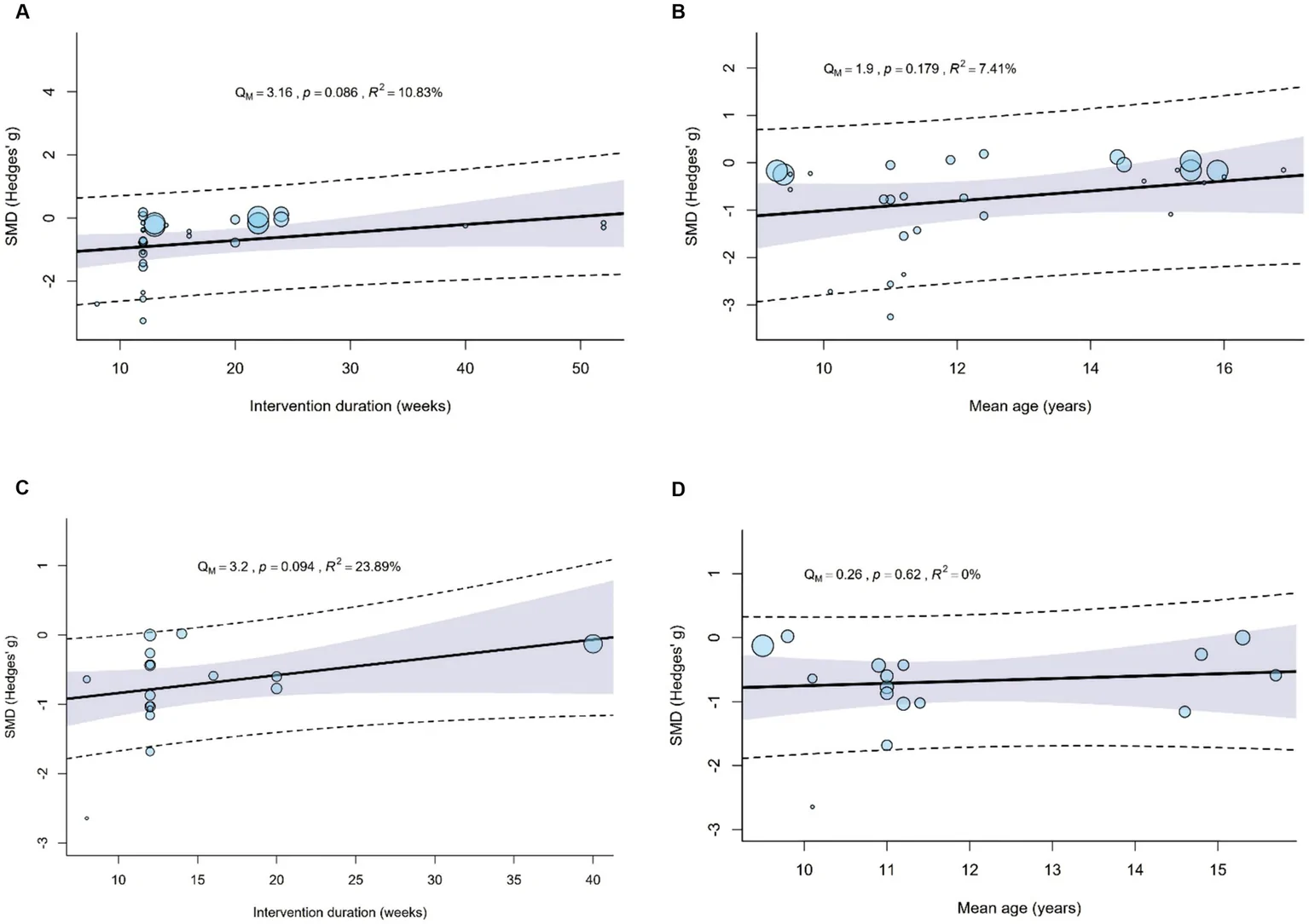
Meta-regression analyses for visceral adipose tissue and waist circumference. **(A)** VAT by intervention duration; **(B)** VAT by mean age; **(C)** Waist circumference by intervention duration; **(D)** Waist circumference by mean age. Solid lines indicate fitted meta-regression relationships, shaded areas represent 95% confidence intervals, and dashed lines represent 95% prediction intervals. Negative Hedges’ *g* values favour exercise.

### 3.6 Publication bias and small-study effects

*Visceral Adipose Tissue*. For VAT, the contour-enhanced funnel plot showed asymmetry, and both three-level Egger-type analyses indicated evidence of small-study effects (both *p* < 0.001), with the conventional Egger test yielding a consistent result. However, trim-and-fill did not impute any potentially missing effect sizes, and the adjusted pooled estimate remained similar to the primary estimate (g = −0.728, 95% CI [−1.043, −0.413], *p* < 0.001). Thus, although small-study effects could not be excluded, the supplementary analyses did not materially alter the direction or statistical significance of the pooled VAT effect.

*Waist Circumference*. Waist circumference also showed evidence of small-study effects in the Egger-based analyses. However, trim-and-fill did not impute any missing effect sizes, and the adjusted estimate remained essentially unchanged, indicating that these signals did not materially alter the favorable direction of the pooled effect.

*Other Secondary Outcomes*. Evidence of small-study effects was mixed across secondary outcomes. SAT showed no clear evidence of small-study effects, whereas body fat percentage, BMI, body weight, fat mass, and VO₂max showed varying signals across the diagnostic analyses. Trim-and-fill imputed potentially missing effect sizes for body fat percentage, BMI, and VO₂max, but the adjusted estimates remained statistically significant and in the same direction as the primary analyses; no missing effect sizes were imputed for SAT, body weight, or fat mass.

Overall, funnel-plot asymmetry and statistical signals of small-study effects were observed for several outcomes but should not be interpreted as direct evidence of publication bias, because such patterns may also arise from heterogeneity and differences in study or measurement characteristics. Trim-and-fill estimates and fail-safe N values are provided in Supplementary Table S8, with corresponding contour-enhanced and trim-and-fill funnel plots presented in Supplementary Figures S5, S6, respectively.

### 3.7 Sensitivity analyses

*Visceral Adipose Tissue*. Seven potential outlying effect sizes were identified for VAT. After their exclusion, the pooled effect was attenuated from g = −0.725 to g = −0.384 (95% CI [−0.558, −0.209], *p* < 0.001), but remained statistically significant. Leave-one-effect-out analyses likewise showed that the pooled effect remained negative and statistically significant regardless of which individual effect size was omitted. Thus, the direction of the VAT effect was stable, although the marked attenuation after outlier removal indicates that several relatively large effects contributed to the magnitude of the primary estimate (Supplementary Tables S9, S10; Supplementary Figures S7, S8).

*Waist Circumference*. Two potential outlying effect sizes were identified for waist circumference. After their removal, the pooled effect remained statistically significant (g = −0.657, 95% CI [−0.926, −0.388], *p* < 0.001), with a marked reduction in heterogeneity. Leave-one-effect-out analyses did not materially alter the direction or statistical significance of the pooled estimate, indicating comparatively good stability (Supplementary Tables S9, S10; Supplementary Figures S7, S8).

*Other Secondary Outcomes*. Sensitivity analyses generally preserved the direction and statistical significance of the pooled effects for the secondary outcomes. Body weight showed greater attenuation after outlier removal, from g = −0.783 to g = −0.316 (95% CI [−0.542, −0.091], *p* = 0.010), while SAT showed weaker stability in leave-one-effect-out analyses, with confidence intervals approaching or crossing the null in some models. Complete outcome-specific results are provided in Supplementary Tables S9, S10 and Supplementary Figures S7, S8.

*Shared-Comparator Sensitivity Analysis*. A split-group sensitivity analysis was conducted to assess the influence of repeated use of shared comparator groups in multi-arm trials. The pooled VAT estimate changed only minimally from g = −0.725 to g = −0.716 (95% CI [−1.105, −0.328], *p* < 0.001), while the waist-circumference estimate changed from g = −0.692 to g = −0.664 (95% CI [−0.964, −0.364], *p* < 0.001). No primary or secondary outcome changed direction or statistical significance after adjustment. Between-modality tests also remained non-significant for VAT (*p* = 0.116 before adjustment; *p* = 0.056 after adjustment) and waist circumference (*p* = 0.184; *p* = 0.267). Thus, repeated use of shared comparator groups did not materially alter the principal pooled estimates or the conclusions regarding between-modality differences (Supplementary Table S11).

*Exercise-Modality Sensitivity Analysis*. Modality moderator analyses were repeated after outcome-specific outlier removal. For VAT, removal of seven outlying effect sizes attenuated the HIIT estimate from g = −1.332 to g = −0.833, but the overall between-modality test remained non-significant (*p* = 0.116). The HIIT+RT subgroup could not be re-estimated because its sole effect size was identified as an outlier. For waist circumference, removal of two outlying effect sizes likewise left the between-modality test non-significant (*p* = 0.817, versus *p* = 0.184 in the primary analysis). Between-modality tests also remained non-significant for all secondary outcomes. Thus, although outlier removal altered the magnitude of some modality-specific estimates, it did not change the conclusion that statistically significant differences between exercise modalities were not demonstrated (Supplementary Table S12).

### 3.8 Certainty of evidence

The certainty of evidence was assessed using the GRADE framework, with outcome-specific judgments and reasons for downgrading provided in Supplementary Table S7.

*Visceral Adipose Tissue*. The certainty of evidence for VAT was rated as moderate and downgraded for serious inconsistency, reflecting substantial heterogeneity (I^2^ = 89.71%) and a 95% prediction interval crossing the null. The direction of the pooled effect remained stable across RVE and sensitivity analyses, although its magnitude was attenuated after outlier removal. Possible small-study effects were identified, but trim-and-fill did not impute missing effect sizes or materially alter the pooled estimate; therefore, no additional downgrade for publication bias was applied.

*Waist Circumference*. The certainty of evidence for waist circumference was also rated as moderate and downgraded primarily for inconsistency. Heterogeneity was moderate (I^2^ = 58.36%), and the 95% prediction interval crossed the null, indicating uncertainty in the magnitude of the comparative effect across future study settings. However, the pooled effect remained stable across RVE, outlier-removal, leave-one-effect-out, and shared-comparator sensitivity analyses, supporting the robustness of the overall direction of effect.

*Secondary Outcomes*. The certainty of evidence for body fat percentage, fat mass, and VO₂max was rated as moderate, primarily because of inconsistency arising from substantial heterogeneity and prediction intervals indicating variability across study settings. Certainty was rated as low for SAT, BMI, and body weight. SAT was downgraded for inconsistency and imprecision, BMI for inconsistency and publication-bias/small-study-effect concerns, and body weight for inconsistency and imprecision, including marked attenuation after outlier removal.

Overall, the evidence was of moderate certainty for both primary outcomes, VAT and waist circumference, as well as for body fat percentage, fat mass, and VO₂max, whereas certainty was low for SAT, BMI, and body weight. Complete domain-specific judgments and rationales are provided in Supplementary Table S7.

## 4 Discussion

This systematic review and three-level meta-analysis examined comparative effects on VAT and waist circumference among exercise-based interventions in children and adolescents with overweight or obesity, while also evaluating whether effect estimates differed across exercise modalities. Across the eligible intervention–comparator contrasts, pooled estimates indicated lower VAT and waist circumference, or greater reductions in these outcomes, in the intervention arms relative to their corresponding comparator arms. However, the magnitude of the comparative effects varied substantially across studies, and the current evidence did not establish the superiority of any single exercise modality. The absence of statistically significant between-modality differences should not be interpreted as physiological equivalence; rather, the optimal training strategy for reducing visceral and central adiposity remains unresolved.

The clinical importance of reducing VAT lies in the fact that obesity-related risk is determined not only by the amount of adipose tissue, but also by its anatomical distribution. Longitudinal imaging studies in adults have shown that greater visceral adiposity is associated with incident cardiovascular disease independently of generalized adiposity, while a higher visceral-to-subcutaneous adipose tissue ratio predicts mortality and cardiac events, indicating that visceral fat distribution carries prognostic information beyond that provided by total adiposity or SAT alone (68, 69). Although such hard cardiovascular endpoints cannot be directly extrapolated to children and adolescents, excess VAT is already associated during youth with an adverse cardiometabolic profile, including dyslipidaemia, elevated blood pressure, and disturbances in glucose metabolism (9, 70, 71). Against this background, the present findings are particularly relevant: the pooled comparative estimate for VAT indicated lower VAT or a greater reduction in the intervention arms relative to their comparators, whereas the corresponding pooled effect for SAT was smaller (Hedges’ g = −0.256) and supported by lower-certainty evidence. This pattern reinforces the importance of considering adipose tissue depots separately rather than assuming a uniform response to exercise. Importantly, improvements in visceral adiposity may occur without substantial weight loss (72, 73). Previous meta-analytic evidence has shown that exercise can reduce VAT in the absence of measurable weight loss, and pediatric intervention studies have similarly reported reductions in visceral fat despite little or no change in body weight (16, 74, 75). Accordingly, exercise-related changes in pediatric obesity may extend beyond what is visible on the weighing scale: modest changes in body weight or BMI may coexist with favorable changes in visceral adiposity that carry clinically relevant information not captured by body size alone.

Although current evidence does not establish the superiority of any single exercise modality for reducing VAT, exercise prescription should consider outcomes beyond adiposity. Different training modalities may differ in their effects on muscular fitness, body composition, and cardiorespiratory capacity, as shown in comparative exercise trials involving adolescents (76, 77). Importantly, the present meta-analysis found a positive pooled comparative effect for VO₂max, indicating greater improvement in the intervention arms relative to their corresponding comparators. Unlike VAT, waist circumference, or other adiposity measures, VO₂max reflects cardiorespiratory capacity and therefore represents a distinct dimension of the health response to exercise. This finding is clinically relevant because cardiorespiratory fitness is an important marker of cardiovascular and overall health in youth (20). However, the between-modality differences in VO₂max were not statistically significant. Given the limited number of studies within several exercise categories, the available evidence does not identify a superior exercise modality for improving cardiorespiratory fitness.

Exercise prescription should therefore consider the combination of outcomes a programme is intended to improve, including visceral adiposity, muscular development, cardiorespiratory fitness, and long-term participation (78, 79).

These findings also have implications for translating exercise efficacy into public health practice. Direct imaging of VAT remains valuable for establishing physiological efficacy, but it is unlikely to be feasible for routine monitoring in schools, community programmes, or primary-care settings (80). The concurrent reduction in waist circumference therefore has practical relevance. Although waist circumference should not be regarded as a surrogate for directly measured VAT, it provides a simple, low-cost, and repeatable indicator of central adiposity that may support intervention monitoring at scale (81, 82). The choice among exercise modalities should likewise consider not only their expected effects on VAT, but also their time requirements, supervision needs, resource demands, acceptability, and capacity for sustained delivery. From this perspective, the relatively large modality-specific point estimate observed for HIIT is noteworthy because interval-based exercise may offer a time-efficient option; however, the non-significant between-modality test means that this finding should not be interpreted as evidence that HIIT is superior to other exercise modalities in either effectiveness or implementation performance (83, 84). Comparative research should therefore evaluate both efficacy and deliverability to determine which exercise strategies can achieve meaningful improvements in central adiposity under real-world pediatric conditions.

### 4.1 Limitations and future directions

Several limitations should be considered when interpreting the comparative findings. First, classifying heterogeneous exercise programmes into seven prespecified categories necessarily simplified differences in intensity, interval structure, resistance content, progression, and training dose. Although explicit decision rules improved reproducibility, most subgroup and meta-regression analyses did not identify statistically significant moderators, and substantial residual heterogeneity remained across several outcomes. This variability may reflect differences in exercise dose, adherence, maturation, baseline adiposity, measurement methods, co-interventions, or other incompletely reported study characteristics. Moreover, modality, intensity, and dose could not be fully disentangled because a comparable measure of exercise volume could not be derived across training modes. Second, several studies had methodological limitations, with 11 studies classified as having high overall risk of bias, although risk-of-bias subgroup analyses did not indicate a consistent systematic shift in pooled effects. Third, although three-level modelling and study-level robust variance estimation were used to address dependent effect sizes, exact sampling covariances for shared-comparator comparisons were not consistently available. A split-group shared-comparator sensitivity analysis did not materially alter the pooled VAT or waist-circumference estimates or the conclusions of the between-modality analyses, but this approximate approach cannot fully substitute for analyses based on exact covariance information. Fourth, variation in VAT assessment and incomplete reporting of participant and intervention characteristics limited further exploration of response heterogeneity, and the restriction to Chinese- and English-language publications may have introduced language bias and reduced the comprehensiveness of the evidence base. Finally, cardiometabolic outcomes such as insulin resistance and dyslipidaemia, as well as changes in lean mass, were not included. Future studies should therefore combine standardized VAT assessment with more precise reporting of exercise dose, adherence, metabolic outcomes, and body-composition changes, while conducting adequately powered head-to-head comparisons of exercise modalities.

## 5 Conclusion

This systematic review and three-level meta-analysis found that, across eligible intervention–comparator contrasts, exercise-based interventions were associated with lower VAT and waist circumference, or greater reductions in these outcomes, among children and adolescents with overweight or obesity. These findings support visceral adiposity as an important and exercise-responsive target in pediatric obesity management, while the accompanying changes in waist circumference indicate potential benefits for central adiposity beyond changes in body weight alone. However, substantial heterogeneity and prediction intervals crossing the null indicate that the magnitude of these comparative effects may vary considerably across study settings. Current evidence also does not establish the consistent superiority of any single exercise modality. Exercise prescription should therefore prioritize developmentally appropriate, feasible, and sustainable programmes while considering broader physiological goals. Future adequately powered head-to-head trials using standardized VAT assessment and more precise characterization of training intensity, dose, adherence, and co-interventions are needed to clarify which exercise strategies produce the most reliable and practically applicable improvements in visceral adiposity.
