# Supplementary material for: Comparative effects of exercise modalities on visceral adipose tissue in children and adolescents with overweight or obesity: a systematic review and three-level meta-analysis
Source: Front Public Health. 2026 Oct 22;14:1922842. doi: 10.3389/fpubh.2026.1922842 (PMC13762740; doi:10.3389/fpubh.2026.1922842)
Supplement: Supplementary file 1 [file Data_Sheet_1.pdf]

SUPPLEMENTARY MATERIAL

Table of Contents

Supplementary Methods

Supplementary Method S1. Search strategy of PubMed, Embase, Cochrane Library, Web of Science, EBSCOhost SPORTDiscus, and CNKI.
Supplementary Method S2. Definitions and decision rules for exercise intervention categories and comparator conditions
Supplementary Method S3. Effect-size computation and estimation of missing standard deviations
Supplementary Method S4. Key statistical terms and interpretative framework used in the three-level meta-analysis

Supplementary Tables

Supplementary Table S1. Study characteristics of the included randomized and partially randomized controlled trials
Supplementary Table S2. Definitions and measurement rules for primary and secondary outcomes
Supplementary Table S3. Risk-of-bias assessment of the included studies using the Cochrane risk-of-bias tool
Supplementary Table S4. Main effects, heterogeneity, prediction intervals, and robust variance estimation results for primary and secondary outcomes
Supplementary Table S5. Subgroup analyses were conducted according to exercise modality, age group, and overall risk of bias.
Supplementary Table S6. Meta-regression analyses of intervention duration and participants' mean age for primary and secondary outcomes
Supplementary Table S7. GRADE summary of findings and certainty of evidence for primary and secondary outcomes
Supplementary Table S8. Trim-and-fill adjusted estimates and fail-safe N values for primary and secondary outcomes
Supplementary Table S9. Comparison of original and outlier-removed pooled effects for primary and secondary outcomes
Supplementary Table S10. Leave-one-effect-out sensitivity analyses for primary and secondary outcomes
Supplementary Table S11. Shared-control-adjusted sensitivity analyses for primary and secondary outcomes
Supplementary Table S12. Exercise-modality analyses before and after outlier removal

Supplementary Figures

Supplementary Figure S1. Risk-of-bias summary plot
Supplementary Figure S2. Orchard plots of the main pooled effects
Supplementary Figure S3. Orchard plots for subgroup analyses
Supplementary Figure S4. Meta-regression plots
Supplementary Figure S5. Contour-enhanced funnel plots
Supplementary Figure S6. Trim-and-fill adjusted funnel plots
Supplementary Figure S7. Outlier-identification forest plots
Supplementary Figure S8. Leave-one-effect-out sensitivity plots

Supplementary Methods S1

Supplementary Methods S1. Search strategy of PubMed, Embase, Cochrane Library, Web of Science, EBSCOhost SPORTDiscus, and CNKI.				
**Database**	**Search Terms**			
	**Exercise**	**Population and obesity**	**Visceral adipose tissue**	**Randomized controlled trial**
PubMed [MeSH Terms / Title/Abstract]	("Exercise"[MeSH Terms] OR Exercises[Title/Abstract] OR Exercise, Physical[Title/Abstract] OR Exercises, Physical[Title/Abstract] OR Physical Exercise[Title/Abstract] OR Physical Exercises[Title/Abstract] OR Exercise, Isometric[Title/Abstract] OR Exercises, Isometric[Title/Abstract] OR Isometric Exercises[Title/Abstract] OR Isometric Exercise[Title/Abstract] OR Exercise, Aerobic[Title/Abstract] OR Aerobic Exercise[Title/Abstract] OR Aerobic Exercises[Title/Abstract] OR Exercises, Aerobic[Title/Abstract] OR Exercise Training[Title/Abstract] OR Exercise Trainings[Title/Abstract] OR Training, Exercise[Title/Abstract] OR Trainings, Exercise[Title/Abstract] OR Physical Activity[Title/Abstract] OR Activities, Physical[Title/Abstract] OR Activity, Physical[Title/Abstract] OR Physical Activities[Title/Abstract] OR Active Breaks[Title/Abstract] OR Activity Breaks[Title/Abstract] OR Acute Exercise[Title/Abstract] OR Acute Exercises[Title/Abstract] OR Exercise, Acute[Title/Abstract] OR Exercises, Acute[Title/Abstract])	("Child"[MeSH Terms] OR children[Title/Abstract] OR "Adolescent"[MeSH Terms] OR Adolescents[Title/Abstract] OR Adolescence[Title/Abstract] OR Youth[Title/Abstract] OR Youths[Title/Abstract] OR Teens[Title/Abstract] OR Teen[Title/Abstract] OR Teenagers[Title/Abstract] OR Teenager[Title/Abstract] OR "Pediatric Obesity"[MeSH Terms] OR Obesity, Pediatric[Title/Abstract] OR Childhood Obesity[Title/Abstract] OR Obesity, Childhood[Title/Abstract] OR Obesity in Childhood[Title/Abstract] OR Child Obesity[Title/Abstract] OR Obesity, Child[Title/Abstract] OR Childhood Onset Obesity[Title/Abstract] OR Obesity, Childhood Onset[Title/Abstract] OR Adolescent Obesity[Title/Abstract] OR Obesity, Adolescent[Title/Abstract] OR Obesity in Adolescence[Title/Abstract] OR Childhood Overweight[Title/Abstract] OR Overweight, Childhood[Title/Abstract] OR Adolescent Overweight[Title/Abstract] OR Overweight, Adolescent[Title/Abstract])	("Intra-Abdominal Fat"[MeSH Terms] OR Fats, Intra-Abdominal[Title/Abstract] OR Intra Abdominal Fat[Title/Abstract] OR Intra-Abdominal Fats[Title/Abstract] OR Fat, Intra-Abdominal[Title/Abstract] OR Fat, Intra Abdominal[Title/Abstract] OR Intra-Abdominal Adipose Tissue[Title/Abstract] OR Adipose Tissue, Intra-Abdominal[Title/Abstract] OR Intra Abdominal Adipose Tissue[Title/Abstract] OR Visceral Fat[Title/Abstract] OR Fats, Visceral[Title/Abstract] OR Fat, Visceral[Title/Abstract] OR Visceral Fats[Title/Abstract] OR Visceral Adipose Tissue[Title/Abstract] OR Adipose Tissue, Visceral[Title/Abstract] OR Abdominal Visceral Fat[Title/Abstract] OR Abdominal Visceral Fats[Title/Abstract] OR Fat, Abdominal Visceral[Title/Abstract] OR Fats, Abdominal Visceral[Title/Abstract] OR Retroperitoneal Fat[Title/Abstract] OR Fat, Retroperitoneal[Title/Abstract] OR Fats, Retroperitoneal[Title/Abstract] OR Retroperitoneal Fats[Title/Abstract] OR Retroperitoneal Adipose Tissue[Title/Abstract] OR Adipose Tissue, Retroperitoneal[Title/Abstract])	(randomized controlled trial[Title/Abstract] OR randomized[Title/Abstract] OR placebo[Title/Abstract])

Embase [Title/Abstract]	'exercise':ab,ti OR 'aerobic exercise':ab,ti OR 'anaerobic exercise':ab,ti OR 'aquatic exercise':ab,ti OR 'arm exercise':ab,ti OR 'athletic performance':ab,ti OR 'breathing exercise':ab,ti OR 'calisthenics':ab,ti OR 'circuit training':ab,ti OR 'closed kinetic chain exercise':ab,ti OR 'continuous training':ab,ti OR 'core stability training':ab,ti OR 'cross training (sport)':ab,ti OR 'dynamic exercise':ab,ti OR 'endurance training':ab,ti OR 'exercise intensity':ab,ti OR 'exergaming':ab,ti OR 'gymnastics':ab,ti OR 'high intensity exercise':ab,ti OR 'interval training':ab,ti OR 'isokinetic exercise':ab,ti OR 'leg exercise':ab,ti OR 'low intensity exercise':ab,ti OR 'moderate intensity exercise':ab,ti OR 'muscle exercise':ab,ti OR 'open kinetic chain exercise':ab,ti OR 'pilates':ab,ti OR 'plyometrics':ab,ti OR 'power training':ab,ti OR 'resistance training':ab,ti OR 'squatting (exercise)':ab,ti OR 'static exercise':ab,ti	'adolescent obesity':ab,ti OR 'body weight disorder':ab,ti OR 'obesity':ab,ti OR 'childhood obesity':ab,ti OR 'Adolescent Obesity':ab,ti OR 'Adolescent Overweight':ab,ti OR 'Child Obesity':ab,ti OR 'Childhood Obesity':ab,ti OR 'Childhood Onset Obesity':ab,ti OR 'Obesity, Adolescent':ab,ti OR 'Obesity, Child':ab,ti OR 'Obesity, Childhood':ab,ti OR 'Obesity, Childhood Onset':ab,ti OR 'Obesity, Pediatric':ab,ti OR 'Obesity in Adolescence':ab,ti OR 'Obesity in Childhood':ab,ti OR 'Overweight, Adolescent':ab,ti OR 'child':ab,ti OR 'children':ab,ti OR 'adolescent':ab,ti OR 'Adolescents':ab,ti OR 'Adolescence':ab,ti OR 'Youth':ab,ti OR 'Youths':ab,ti OR 'Teens':ab,ti OR 'Teenagers':ab,ti OR 'Teen':ab,ti OR 'Teenager':ab,ti	'abdominal fat':ab,ti OR 'intraabdominal fat':ab,ti OR 'intra-abdominal fat':ab,ti OR 'intra-abdominal white adipose tissue':ab,ti OR 'intraperitoneal fat':ab,ti OR 'retroperitoneal fat':ab,ti OR 'retroperitoneal adipose tissue':ab,ti OR 'visceral fat':ab,ti OR 'visceral adipose tissue':ab,ti OR 'abdominal visceral fat':ab,ti OR 'fats visceral':ab,ti OR 'fat visceral':ab,ti OR 'visceral fats':ab,ti OR 'adipose tissue visceral':ab,ti OR 'abdominal visceral fats':ab,ti OR 'fat abdominal visceral':ab,ti OR 'fats abdominal visceral':ab,ti OR 'fat retroperitoneal':ab,ti OR 'fats retroperitoneal':ab,ti OR 'retroperitoneal fats':ab,ti OR 'adipose tissue retroperitoneal':ab,ti	'randomized controlled trial':ab,ti OR 'randomized':ab,ti OR 'placebo':ab,ti
Cochrane Library [Title/Abstract/Keywords]	(Exercises):ti,ab,kw OR (Exercise, Physical):ti,ab,kw OR (Exercises, Physical):ti,ab,kw OR (Physical Exercise):ti,ab,kw OR (Physical Exercises):ti,ab,kw OR (Exercise, Isometric):ti,ab,kw OR (Exercises, Isometric):ti,ab,kw OR (Isometric Exercises):ti,ab,kw OR (Isometric Exercise):ti,ab,kw OR (Exercise, Aerobic):ti,ab,kw OR (Aerobic Exercise):ti,ab,kw OR (Aerobic Exercises):ti,ab,kw OR (Exercises, Aerobic):ti,ab,kw OR (Exercise Training):ti,ab,kw OR (Exercise Trainings):ti,ab,kw OR (Training, Exercise):ti,ab,kw OR (Trainings, Exercise):ti,ab,kw OR (Physical Activity):ti,ab,kw OR (Activities, Physical):ti,ab,kw OR (Activity, Physical):ti,ab,kw OR (Physical Activities):ti,ab,kw OR (Active Breaks):ti,ab,kw OR (Activity Breaks):ti,ab,kw OR	(Adolescent Obesity):ti,ab,kw OR (Adolescent Overweight):ti,ab,kw OR (Child Obesity):ti,ab,kw OR (Childhood Obesity):ti,ab,kw OR (Childhood Onset Obesity):ti,ab,kw OR (Obesity, Adolescent):ti,ab,kw OR (Obesity, Child):ti,ab,kw OR (Obesity, Childhood):ti,ab,kw OR (Obesity, Childhood Onset):ti,ab,kw OR (Obesity, Pediatric):ti,ab,kw OR (Obesity in Adolescence):ti,ab,kw OR (Obesity in Childhood):ti,ab,kw OR (Overweight, Adolescent):ti,ab,kw OR (child):ti,ab,kw OR (children):ti,ab,kw OR (adolescent):ti,ab,kw OR (Adolescents):ti,ab,kw OR (Adolescence):ti,ab,kw OR (Youth):ti,ab,kw OR (Youths):ti,ab,kw OR (Teens):ti,ab,kw OR (Teen):ti,ab,kw OR (Teenagers):ti,ab,kw OR (Teenager):ti,ab,kw	(Fats, Intra-Abdominal):ti,ab,kw OR (Intra Abdominal Fat):ti,ab,kw OR (Intra-Abdominal Fats):ti,ab,kw OR (Fat, Intra-Abdominal):ti,ab,kw OR (Fat, Intra Abdominal):ti,ab,kw OR (Intra-Abdominal Adipose Tissue):ti,ab,kw OR (Adipose Tissue, Intra-Abdominal):ti,ab,kw OR (Intra Abdominal Adipose Tissue):ti,ab,kw OR (Visceral Fat):ti,ab,kw OR (Fats, Visceral):ti,ab,kw OR (Fat, Visceral):ti,ab,kw OR (Visceral Fats):ti,ab,kw OR (Visceral Adipose Tissue):ti,ab,kw OR (Adipose Tissue, Visceral):ti,ab,kw OR (Abdominal Visceral Fat):ti,ab,kw OR (Abdominal Visceral Fats):ti,ab,kw OR (Fat, Abdominal Visceral):ti,ab,kw OR (Fats, Abdominal Visceral):ti,ab,kw OR (Retroperitoneal Fat):ti,ab,kw OR (Fat, Retroperitoneal):ti,ab,kw OR (Fats, Retroperitoneal):ti,ab,kw OR (Retroperitoneal Fats):ti,ab,kw OR (Retroperitoneal	—

	(Acute Exercise):ti,ab,kw OR (Acute Exercises):ti,ab,kw OR (Exercise, Acute):ti,ab,kw OR (Exercises, Acute):ti,ab,kw		Adipose Tissue):ti,ab,kw OR (Adipose Tissue, Retroperitoneal):ti,ab,kw	
Web of Science [Topic]	TS=("Exercises" OR "Exercise, Physical" OR "Exercises, Physical" OR "Physical Exercise" OR "Physical Exercises" OR "Exercise, Isometric" OR "Exercises, Isometric" OR "Isometric Exercises" OR "Isometric Exercise" OR "Exercise, Aerobic" OR "Aerobic Exercise" OR "Aerobic Exercises" OR "Exercises, Aerobic" OR "Exercise Training" OR "Exercise Trainings" OR "Training, Exercise" OR "Trainings, Exercise" OR "Physical Activity" OR "Activities, Physical" OR "Activity, Physical" OR "Physical Activities" OR "Active Breaks" OR "Activity Breaks" OR "Acute Exercise" OR "Acute Exercises" OR "Exercise, Acute" OR "Exercises, Acute")	TS=("Pediatric Obesity" OR "Obesity, Pediatric" OR "Childhood Obesity" OR "Obesity, Childhood" OR "Obesity in Childhood" OR "Child Obesity" OR "Obesity,Child" OR "Childhood Onset Obesity" OR "Obesity, Childhood Onset" OR "Adolescent Obesity" OR "Obesity, Adolescent" OR "Obesity in Adolescence" OR "Childhood Overweight" OR "Overweight, Childhood" OR "Adolescent Overweight" OR "Overweight,Adolescent" OR "child" OR "children" OR "adolescent" OR "Adolescents" OR "Adolescence" OR "Youth" OR "Youths" OR "Teens" OR "Teen" OR "Teenagers" OR "Teenager")	TS=("Fats, Intra-Abdominal" OR "Intra Abdominal Fat" OR "Intra-Abdominal Fats" OR "Fat, Intra-Abdominal" OR "Fat, Intra Abdominal" OR "Intra-Abdominal Adipose Tissue" OR "Adipose Tissue, Intra-Abdominal" OR "Intra Abdominal Adipose Tissue" OR "Visceral Fat" OR "Fats, Visceral" OR "Fat, Visceral" OR "Visceral Fats" OR "Visceral Adipose Tissue" OR "Adipose Tissue, Visceral" OR "Abdominal Visceral Fat" OR "Abdominal Visceral Fats" OR "Fat, Abdominal Visceral" OR "Fats, Abdominal Visceral" OR "Retroperitoneal Fat" OR "Fat, Retroperitoneal" OR "Fats, Retroperitoneal" OR "Retroperitoneal Fats" OR "Retroperitoneal Adipose Tissue" OR "Adipose Tissue, Retroperitoneal")	TS=("randomized controlled trial" OR "randomized" OR "placebo")
EBSCOhost [Abstract]	AB (Exercises) OR AB (Exercise, Physical) OR AB (Exercise Training) OR AB (Physical Exercise) OR AB (Physical Exercises) OR AB (Exercise, Isometric) OR AB (Exercises, Isometric) OR AB (Isometric Exercises) OR AB (Training, Exercise) OR AB (Exercise, Aerobic) OR AB (Aerobic Exercise) OR AB (Exercises, Acute)	AB (Pediatric Obesity) OR AB (Obesity, Pediatric) OR AB (Childhood Obesity) OR AB (Obesity, Childhood) OR AB (Child Obesity) OR AB (Obesity, Child) OR AB (Adolescent Obesity) OR AB (Obesity, Adolescent) OR AB (Overweight, Adolescent) OR AB (Obesity, Childhood Onset) OR AB (child) OR AB (Youth)	AB (Fats, Intra-Abdominal) OR AB (Intra Abdominal Fat) OR AB (Fat, Intra-Abdominal) OR AB (Obesity, Childhood) OR AB (Adipose Tissue, Intra-Abdominal) OR AB (Fats, Visceral) OR AB (Fat, Abdominal Visceral) OR AB (Fat, Retroperitoneal) OR AB (Retroperitoneal Adipose Tissue) OR AB (Adipose Tissue, Retroperitoneal)	AB (randomized controlled trial) OR AB (randomized) OR AB (placebo)
**CNKI [Topic / Title / Abstract / Keywords]**	(运动 OR 体育活动 OR 身体活动 OR 有氧运动 OR 运动训练 OR 抗阻训练 OR 阻力训练 OR 力量训练 OR 间歇训练 OR 高强度间歇训练 OR HIIT OR 联合训练 OR 综合训练)	(儿童 OR 青少年 OR 少年 OR 学生 OR 肥胖 OR 超重 OR 儿童肥胖 OR 青少年肥胖 OR 单纯性肥胖 OR 肥胖儿童 OR 肥胖青少年)	(内脏脂肪 OR 内脏脂肪组织 OR 腹内脂肪 OR 腹腔脂肪 OR 腹部内脏脂肪 OR 内脏脂肪面积 OR 内脏脂肪含量 OR VAT OR visceral fat OR visceral adipose tissue)	(随机 OR 随机对照 OR 随机对照试验 OR 随机分组 OR 对照试验 OR RCT)

Supplementary Method S2.

Supplementary Method S2. Definitions and decision rules for exercise intervention categories and comparator conditions		
**Category**	**Full name**	**Definition and decision rules**
**CON**	Control condition	Comparator conditions without additional structured exercise training. Eligible control conditions included no exercise, wait-list control, usual care, habitual lifestyle, routine school physical education, health education, nutritional advice, dietary counselling without structured exercise, light stretching, delayed intervention, or other minimal-exercise conditions. When dietary, educational, behavioural, or other lifestyle components were present, the comparison was retained when it permitted a meaningful evaluation of the exercise modality, intensity, or training structure under investigation. When another eligible structured exercise intervention served as the comparator, that arm retained its corresponding exercise-category classification rather than being coded as CON.
**AE[M]**	Moderate-intensity aerobic exercise	Aerobic exercise performed predominantly at moderate intensity. This category included continuous or intermittent aerobic activities such as treadmill exercise, cycling, running, walking, aerobic games, active play, exergaming, or school-based aerobic activities when the prescribed or reported intensity was approximately 45%–65% VO_2max, 50%–65% heart rate reserve, or 65%–75% maximum heart rate. When intensity was described qualitatively as moderate and no contradictory physiological indicator was reported, the intervention was classified as AE[M].
**AE[MV]**	Moderate-to-vigorous aerobic exercise	Aerobic exercise involving a moderate-to-vigorous intensity range, or interventions in which the prescribed intensity crossed both moderate and vigorous zones but could not be separated into distinct effect sizes. This category was used when the program combined moderate and vigorous aerobic activities, when intensity was reported as moderate-to-vigorous physical activity, or when the available information indicated a broad aerobic training range rather than a single intensity level.
**AE[V]**	Vigorous-intensity aerobic exercise	Aerobic exercise performed predominantly at vigorous intensity. This category included continuous or repeated aerobic exercise such as running, treadmill training, cycling, aerobic games, active play, or school-based aerobic exercise when the prescribed or reported intensity exceeded 65% VO_2max, exceeded 65% heart rate reserve, or exceeded 75% maximum heart rate. When the original study reported vigorous aerobic training or comparable high-intensity aerobic activity without an interval-training structure, the intervention was classified as AE[V] rather than HIIT.
**RT**	Resistance training	Structured resistance exercise designed to improve muscular strength, muscular endurance, or neuromuscular function. Eligible interventions included machine-based resistance training, free weights, weight machines, elastic resistance, body-weight resistance exercise, circuit-based resistance programs, core-strength training, or progressive strength training. When intensity was reported, programs at approximately ≥50% one-repetition maximum or with progressive overload were classified as RT.
**AE+RT**	Aerobic exercise combined with resistance training	Multicomponent exercise programs combining aerobic exercise with resistance training within the same intervention arm. This category was used when both aerobic and resistance components were planned as core components of the intervention and the aerobic component was not primarily organized as high-intensity interval training. If aerobic and resistance elements were delivered together but the high-intensity interval component was the dominant aerobic stimulus, the intervention was classified as HIIT+RT.

**HIIT**	High-intensity interval training	Exercise programs characterized by repeated bouts of high-intensity exercise interspersed with recovery periods. This category included high-intensity interval running, treadmill intervals, cycling intervals, sprint interval training, or repeated short-to-long high-intensity bouts when the active intervals were prescribed at vigorous or near-maximal intensity, commonly exceeding 65% VO_2max, exceeding 65% heart rate reserve, exceeding 75% maximum heart rate, or based on maximal aerobic speed, peak power, or all-out sprint efforts. Sprint interval training was classified as HIIT because it shared the defining interval structure and high-intensity physiological stimulus.
**HIIT+RT**	High-intensity interval training combined with resistance training	Combined programs in which HIIT or sprint interval training was delivered together with resistance training, strength training, or core-strength exercise. This category was used when both the interval-training component and the resistance or strength component were substantive parts of the intervention. Programs were classified as HIIT+RT rather than AE+RT when the aerobic component was explicitly interval-based and performed at high intensity.

Note. AE[M], moderate-intensity aerobic exercise; AE[MV], moderate-to-vigorous aerobic exercise; AE[V], vigorous-intensity aerobic exercise; AE+RT, aerobic exercise combined with resistance training; CON, control condition; HIIT, high-intensity interval training; HIIT+RT, high-intensity interval training combined with resistance training; RT, resistance training; HRR, heart rate reserve; MHR, maximum heart rate; VO_2max, maximal oxygen uptake. Exercise categories and intensity thresholds were adapted from previously published exercise-classification frameworks, with operational decision rules refined for the present review(1–3).

Supplementary Method S3. Effect-size computation and estimation of missing standard deviations

Effect sizes were calculated to quantify comparative effects across eligible intervention–comparator contrasts on visceral adipose tissue (VAT) and related secondary outcomes among children and adolescents with overweight or obesity. The small-sample-corrected standardized mean difference, Hedges' g, was used as the primary effect-size metric because the included studies reported outcomes using different units, measurement techniques, and reporting formats. The primary outcomes were VAT or an equivalent visceral fat indicator and WC. Secondary outcomes included SAT, body fat percentage, BMI or BMI z-score, body weight, total fat mass, and cardiorespiratory fitness assessed using VO_2max, VO_2peak, or comparable aerobic fitness indicators.

For each eligible comparison, group means, standard deviations (SDs), and sample sizes were extracted for the intervention and comparator groups whenever they were directly available from the original reports. When both change scores and postintervention values were reported, change scores from baseline to the end of the intervention were preferentially used because they better reflected the intervention-related change over time. When change scores were not reported directly but sufficient baseline and post-intervention data were available, change scores were derived from the reported data. Post-intervention values alone were not combined with change-score effect sizes in the primary quantitative synthesis.

The direction of effect sizes was coded according to the clinical meaning of each outcome. For VAT, SAT, body fat percentage, waist circumference, BMI, body weight, and fat mass, negative values indicated lower values or greater reductions in the intervention group relative to the comparator group. For VO_2max or related cardiorespiratory fitness outcomes, positive values indicated greater improvement in the intervention group relative to the comparator group. Before analysis, all extracted

outcomes were checked to ensure that the direction of scoring and interpretation was consistent with these coding rules.

Hedges' g was calculated as the standardized difference between the intervention and comparator groups, corrected for small-sample bias. For continuous outcomes, the standardized mean difference was calculated using the pooled SD and then multiplied by the small-sample correction factor. Sampling variances and standard errors were calculated for each effect size and used as the known Level 1 sampling error in the three-level meta-analytic models.

When SDs were not directly reported, they were estimated from other available summary statistics using established methods recommended in the Cochrane Handbook and related methodological studies. When a standard error (SE) was reported, the SD was calculated as:

$SD = SE \times \sqrt{n}$

where n denotes the sample size of the corresponding group.

When a 95% confidence interval (CI) around a group mean was reported, the SD was estimated from the CI width using:

$SD = \sqrt{n} \times (\text{Upper limit} - \text{Lower limit}) / 3.92$

For studies with small sample sizes, t-based critical values were considered where appropriate:

$SD = \sqrt{n} \times (\text{Upper limit} - \text{Lower limit}) / (2 \times t_{0.975,df})$

where $t_{0.975,df}$ denotes the two-sided 97.5th percentile of the t distribution with the relevant degrees of freedom.

When dispersion was reported as an interquartile range (IQR), the SD was approximated as:

$SD = IQR / 1.35$

When only medians with IQRs or ranges were reported, means and SDs were estimated using established conversion methods, including those proposed by Wan et al. and Luo et al., provided that the data distribution was not clearly inappropriate for such conversion. When only minimum and maximum values were available, range-based conversion methods were applied with consideration of the sample size to reduce potential overestimation or underestimation of the SD.

When outcomes were reported as change scores without corresponding change-score SDs, change-score SDs were estimated using the standard pre-post correlation formula:

$SD_{change} = \sqrt{(SD_{baseline}^2 + SD_{post}^2 - 2r \times SD_{baseline} \times SD_{post})}$

where r denotes the assumed correlation between baseline and postintervention values. When the original study did not provide sufficient information to derive the correlation empirically, a conservative correlation coefficient of 0.50 was assumed. This assumption was applied consistently across eligible outcomes when change-score SDs had to be derived from baseline and postintervention SDs.

For studies reporting multiple eligible outcomes, multiple exercise arms, multiple intervention intensities, or more than one eligible measurement index, separate effect

sizes were calculated and retained. For multiarm randomized trials, each eligible exercise-control comparison was extracted separately when the exercise arms represented distinct intervention categories or clearly different exercise doses. When a shared control group was used across multiple exercise arms, the dependency among effect sizes derived from the same study was handled within the three-level random-effects meta-analytic framework, with additional use of study-level robust variance estimation to examine the stability of statistical inference.

When multiple publications reported data from the same trial, they were treated as one study. Data were extracted from the most complete report and supplemented by companion publications when necessary. If essential information required for effect-size calculation could not be obtained from the main article, supplementary materials, figures, trial registry records, or other available statistical information, the corresponding authors were contacted where possible. Outcomes for which the necessary data remained unavailable were excluded from the quantitative synthesis but retained in the qualitative description when relevant.

All effect sizes were calculated as Hedges' g with corresponding sampling variances and standard errors. Effect-size computation and meta-analytic procedures were performed in R using the metafor package. The final effect-size dataset included study identifiers, effect-size identifiers, exercise categories, intervention and comparator arms, sample sizes, means or change scores, SDs, Hedges' g, standard errors, sampling variances, outcome domains, intervention duration, and participant age information used for subgroup analyses and meta-regression.

Supplementary Method S4.

Supplementary Method S4. Key statistical terms and interpretative framework used in the three-level meta-analysis

Term	Explanation
**Standardized mean difference (Hedges' g)**	The standardized mean difference was used to quantify the effects of exercise interventions across studies that reported the primary and secondary outcomes using different units, measurement methods, or reporting formats. Hedges' g was used because it applies a small-sample correction and allows continuous outcomes such as VAT, SAT, body fat percentage, waist circumference, BMI, body weight, fat mass, and VO_2max to be synthesized on a common metric.
**Direction of effect**	Effect sizes were coded according to the clinical meaning of each outcome. For adiposity-related outcomes, including VAT, SAT, body fat percentage, waist circumference, BMI, body weight, and fat mass, negative values indicated lower values or greater reductions in the intervention group relative to the comparator group. For VO_2max or comparable cardiorespiratory fitness outcomes, positive values indicated greater improvement in the intervention group relative to the comparator group.
**95% confidence interval (CI)**	The 95% confidence interval indicates the precision of the pooled mean effect. When the 95% CI did not cross zero, the pooled effect was considered statistically significant at the conventional two-sided 0.05 level. Wider confidence intervals indicate greater statistical uncertainty around the estimated mean effect.
**p value and statistical significance**	Statistical significance was evaluated using two-sided p values and 95% confidence intervals. A p value below 0.05 was considered statistically significant. However, p values were interpreted together with effect-size magnitude, confidence intervals, prediction intervals, heterogeneity, robust variance estimation, sensitivity analyses, and certainty of evidence rather than as the sole indicator of importance.

**Three-level meta-analysis**	A three-level meta-analytic model was used because several studies contributed more than one effect size, for example by reporting multiple outcomes, multiple exercise arms, different intervention doses, or multiple eligible comparisons. The model accounts for the nested data structure by separating sampling error, within-study variability among multiple effect sizes, and between-study variability across independent studies.
**Level 1 variance**	Level 1 represents sampling error within each observed effect size. This component reflects the uncertainty associated with estimating an intervention effect from a finite sample and is represented by the known sampling variance of each Hedges' g estimate.
**Level 2 variance**	Level 2 represents within-study heterogeneity, that is, variability among multiple effect sizes derived from the same study. This may occur when a study reports several outcomes, multiple exercise arms, more than one eligible measurement index, or distinct intervention-control comparisons.
**Level 3 variance**	Level 3 represents between-study heterogeneity, that is, variability in true effects across independent studies. This may reflect differences in participant characteristics, exercise modality, intervention intensity or duration, supervision, comparator conditions, outcome measurement, and study quality.
**Heterogeneity**	Heterogeneity refers to variation in effect sizes beyond what would be expected from sampling error alone. In this review, heterogeneity was assessed using the Q statistic, I^2, and variance components from the three-level model.
**Q statistic**	The Q statistic tests whether the observed variation in effect sizes is greater than expected by chance. A statistically significant Q value suggests the presence of heterogeneity, but Q is sensitive to the number of effect sizes and should be interpreted together with I^2 and model-based variance components.
**I^2 statistic**	I^2 describes the proportion of total variability in observed effects that is attributable to heterogeneity rather than sampling error. Values of approximately 25%, 50%, and 75% are commonly interpreted as low, moderate, and substantial heterogeneity, respectively, although interpretation should consider the research context and number of studies.
**I^2 in the three-level model**	In the three-level model, total heterogeneity can be decomposed into Level 2 and Level 3 components. Level 2 I^2 reflects the proportion of variability attributable to differences among effect sizes within the same study, whereas Level 3 I^2 reflects variability between studies. This decomposition provides a more informative description of dependency and heterogeneity than a conventional two-level model.
**Prediction interval**	The prediction interval estimates the range in which the true effect of a future comparable study may fall. Unlike the 95% CI, which reflects uncertainty around the pooled mean effect, the prediction interval incorporates between-study heterogeneity and provides information about the expected dispersion of effects across future settings.
**Restricted maximum likelihood (REML)**	Restricted maximum likelihood was used to estimate variance components in the random-effects models. REML is commonly used in meta-analysis because it generally provides less biased estimates of heterogeneity than some alternative estimation methods, particularly when the number of studies is limited.

**Robust variance estimation (RVE)**	Robust variance estimation was used to obtain standard errors, confidence intervals, and p values that are less sensitive to misspecification of the within-study covariance structure. This is particularly relevant when multiple dependent effect sizes are extracted from the same study and the exact correlations among them are unknown.
**Subgroup analysis**	Subgroup analyses were used to explore whether exercise effects differed across categorical moderators, including exercise modality and age group. These analyses were considered exploratory and were interpreted cautiously, especially when the number of studies or effect sizes within a subgroup was small.
**Meta-regression**	Meta-regression was used to examine whether continuous study-level or intervention-level characteristics were associated with variations in effect sizes. In this review, intervention duration and participants' mean age were examined as continuous moderators. Regression coefficients indicate the estimated change in effect size associated with a one-unit increase in the moderator.
**Moderator analysis**	Moderator analysis refers to statistical analyses used to examine whether study-level or intervention-level characteristics explain differences in effect sizes. Subgroup analyses and meta-regression are both forms of moderator analysis. Because these analyses are observational at the study level, they should not be interpreted as causal evidence.
**Publication bias**	Publication bias refers to the possibility that studies with statistically significant or favorable findings are more likely to be published than studies with null or unfavorable findings. Publication bias and small-study effects were assessed using contour-enhanced funnel plots and Egger-type regression tests when the number of effect sizes was sufficient.
**Contour-enhanced funnel plot**	A contour-enhanced funnel plot displays effect sizes against their precision and adds regions corresponding to conventional levels of statistical significance. This helps distinguish asymmetry that may be related to publication bias from asymmetry caused by heterogeneity, small-study effects, outcome measurement differences, or other methodological factors.
**Egger-type regression test**	Egger-type regression tests examine the association between effect-size estimates and their standard errors or sampling variances. A statistically significant association may indicate small-study effects or funnel plot asymmetry. In this review, Egger-type tests were implemented within the three-level meta-analytic framework where applicable.
**Trim-and-fill method**	The trim-and-fill method is a supplementary approach used to estimate the potential influence of missing studies under assumptions of funnel plot asymmetry. Because the method relies on strong assumptions and may be unreliable in the presence of substantial heterogeneity, it was interpreted as an exploratory sensitivity analysis rather than definitive evidence of publication bias.
**Fail-safe N**	Fail-safe N estimates the number of unpublished null-effect studies that would be required to reduce an observed pooled effect to nonsignificance. Larger fail-safe N values suggest greater robustness, but this statistic should be interpreted cautiously and alongside other publication-bias diagnostics and certainty-of-evidence assessments.
**Sensitivity analysis**	Sensitivity analyses were conducted to examine whether the main findings were robust to influential data points and analytic decisions. These analyses included outlier diagnostics, refitting models after excluding outliers, and leave-one-effect-out procedures.

**Leave-one-effect-out analysis**	Leave-one-effect-out analysis involves removing one effect size at a time and refitting the three-level meta-analytic model to evaluate whether the pooled estimate is disproportionately influenced by any single data point. Stable pooled estimates across these analyses indicate greater robustness.
**Risk of bias**	Risk of bias refers to the extent to which the design, conduct, analysis, or reporting of an included randomized trial may systematically distort the estimated intervention effect. In this review, risk of bias was assessed using the Cochrane risk-of-bias tool and considered when interpreting the robustness and certainty of the evidence.
**GRADE certainty of evidence**	The GRADE approach was used to evaluate the certainty of evidence for each outcome. Certainty was judged across domains including risk of bias, inconsistency, indirectness, imprecision, and publication bias. Evidence certainty was rated as high, moderate, low, or very low.
**Orchard plot**	Orchard plots were used to visualize meta-analytic results in this review. Compared with conventional forest plots, orchard plots provide a compact visual summary of pooled effects, confidence intervals, prediction intervals, and heterogeneity, making them suitable for presenting multiple outcomes or subgroup results in three-level meta-analytic models.

Supplementary Tables
Supplementary Table S1.

Supplementary Table S1. Study characteristics of the included randomized and partially randomized controlled trials

ID	Study (year)	Country	Design	Group	Sample (M/F)	Age	Diagnosis	Intervention category	Intervention description	Dose / frequency	Duration	Reported outcomes	Measurement
1	Owens(51) 1999	United States (US)	RCT	Physical training	35 (12/23)	9.5 ± 0.2 SE	Obese children aged 7–11 yr; triceps skinfold >85th percentile	AE[MV]	Machine-based aerobic exercise plus active games; no dietary intervention	5 d/wk; 40 min/session; target HR >150 bpm; mean HR 157 bpm	4 months	VAT; SAAT; TFM; FFM; %BF; physical activity; cardiovascular fitness	MRI: VAT/SAAT volume (cm³); DXA: TFM, FFM, %BF; 7-d recall: physical activity; submaximal cycling HR: fitness
1	Owens 1999	United States (US)	RCT	Control	39 (13/26)	9.4 ± 0.2 SE	Same as above	CON	Non exercising control; no dietary intervention	NR	4 months	Same as above	Same as above
2	Barbeau(52) 2007	USA	RCT	After-school PA	118 (0/118)	9.5; SD NR	Black girls aged 8–12 yr; entire adiposity range	AE[V]	After-school PA program including skills instruction, aerobic PA, and toning/stretching	80 min PA/day; 35 min MVPA; HR target >150 bpm; mean HR 160 bpm	10 months	BMI; waist circumference; FM; FFST; %BF; BMC; BMD; VAT; SAAT; CV fitness; PA	MRI: VAT/SAAT volume (cm³); DXA: FM, FFST, %BF, BMC, BMD; treadmill test: CV fitness; 7-d recall: PA
2	Barbeau 2007	USA	RCT	Control	83 (0/83)	9.5; SD NR	Same as above	CON	No intervention	NR	10 months	Same as above	Same as above
3	Davis 2011(58)	USA	3-arm RCT	CT + MI	12 (0/12)	15.7 ± 1.2	OW/OB Latina girls aged 14–18 yr; BMI ≥85th percentile	AE+RT	Circuit training plus motivational interviewing	2 d/wk; 60–90 min/session; HR 70–85% HRmax; 4 individual + 4 group MI sessions	16 wk	VO_2max; strength; waist; FM; lean mass; VAT; SAT; hepatic fat; glucose;	Treadmill test: VO_2max; 1RM: strength; DXA: FM/lean mass; MRI: VAT/SAT volume (L), hepatic fat; blood

												insulin; HOMA-IR; PA; diet	tests: glucose/insulin; accelerometer: PA; 3-d diet record
3	Davis 2011	USA	3-arm RCT	CT	14 (0/14)	15.7 ± 1.1	Same as above	AE+RT	Circuit training including aerobic and strength exercises	2 d/wk; 60–90 min/session; HR 70–85% HRmax	16 wk	Same as above	Same as above
3	Davis 2011	USA	3-arm RCT	Control	12 (0/12)	15.8 ± 1.0	Same as above	CON	No intervention during study period; delayed brief CT offered after post-test	NR	16 wk	Same as above	Same as above
4	Saelens(54) 2011	USA	Pilot RCT	STANDARD	15 (7/8)	10.1 ± 1.1	Overweight children; BMI >85th percentile; parent BMI ≥25	CON	Family-based behavioral weight management mainly targeting diet; general PA advice	14 weekly visits; PA advice ≥60 min/d on most days	14 wk	BMI; BMI z-score; % overweight; whole-body fat; %BF; lean mass; waist; total abdominal fat; VAT; SAT; PA; diet	DXA: fat/lean mass; MRI: abdominal fat, VAT/SAT volume (cm³); accelerometer: PA; 3-d food logs: diet
4	Saelens 2011	USA	Pilot RCT	ADDED	14 (6/8)	9.8 ± 1.2	Same as above	AE[MV]	Same dietary program plus targeted MVPA increase using pedometers, goals, rewards, and PA strategies	14 weekly visits; target ≥90 min/d MVPA on ≥6 d/wk	14 wk	Same as above	Same as above
5	Davis(53) 2012	USA	3-arm RCT	Low-dose exercise	71 (31/40)	9.3 ± 0.9	OW/OB sedentary children aged 7–11 yr; BMI	AE[V]	Aerobic games, jump rope, modified basketball/soccer	20 min/d; 5 d/wk; mean HR 166 bpm; 7.5 METs; attendance 85%	13 wk	Insulin AUC; glucose; insulin; %BF; VAT; SAT;	OGTT: insulin/glucose; DXA: %BF; MRI: VAT/SAT volume

							≥85th percentile					BMI z-score; peak VO_2	(cm³); treadmill: peak VO_2
5	Davis 2012	USA	3-arm RCT	High-dose exercise	73 (33/40)	9.4 ± 1.2	Same as above	AE[V]	Same aerobic activities; two 20-min bouts/day	40 min/d; 5 d/wk; mean HR 165 bpm; 7.5 METs; attendance 84%	13 wk	Same as above	Same as above
5	Davis 2012	USA	3-arm RCT	Control	78 (30/48)	9.4 ± 1.1	Same as above	CON	Usual physical activity; monthly lifestyle education offered	NR	13 wk	Same as above	Same as above
6	Lee 2012(55)	USA	3-arm RCT	Aerobic exercise	16 (16/0)	15.2 ± 1.9	Obese inactive adolescent boys aged 12–18 yr; BMI ≥95th percentile; Tanner III–V	AE[MV]	Treadmill, elliptical, or stationary bike exercise; no caloric restriction	3 d/wk; 60 min/session; 60–75% VO_2peak	3 mo	WC; total adiposity; total AT; VAT; abdominal SAT; liver fat; IMCL; insulin sensitivity; insulin secretion; VO_2peak	MRI: AT, VAT mass (kg), SAT, skeletal muscle; MRS: liver fat, IMCL; DXA: FFM; clamp: insulin sensitivity/secretion; treadmill: VO_2peak
6	Lee 2012	USA	3-arm RCT	Resistance exercise	16 (16/0)	14.6 ± 1.5	Same as above	RT	Whole-body resistance training using weight-stack equipment; no caloric restriction	3 d/wk; 60 min/session; 10 exercises; 8–12 reps; 60% RM then to fatigue	3 mo	Same as above	MRI: AT, VAT, SAT, skeletal muscle; MRS: liver fat, IMCL; DXA: FFM; clamp: insulin sensitivity/secretion; RM test: strength
6	Lee 2012	USA	3-arm RCT	Control	13 (13/0)	14.8 ± 1.4	Same as above	CON	Maintained usual leisure-time activity; no structured exercise	NR	3 mo	Same as above	Same as above

7	Lee 2013a(16)	USA	3-arm RCT	Aerobic exercise	16 (0/16)	14.6 ± 1.9	Obese inactive girls aged 12–18 yr; BMI ≥95th percentile; Tanner III–V	AE[MV]	Treadmill/elliptical aerobic exercise; no caloric restriction	3 d/wk; 60 min/session; 60–75% VO_2peak	3 mo	BMI; waist; %BF; total AT; VAT; ASAT; liver fat; IMAT; insulin sensitivity; VO_2peak; strength	MRI: AT, VAT/ASAT area (cm²); MRS: liver fat; CT: IMAT; clamp: insulin sensitivity; treadmill: VO_2peak; 1RM: strength
7	Lee 2013a	USA	3-arm RCT	Resistance exercise	16 (0/16)	14.8 ± 1.9	Same as above	RT	Whole-body machine-based resistance training; no caloric restriction	3 d/wk; 60 min/session; 10 exercises; 8–12 reps; 60% 1RM then to fatigue	3 mo	Same as above	Same as above
7	Lee 2013a	USA	3-arm RCT	Control	12 (0/12)	15.0 ± 2.2	Same as above	CON	No structured physical activity	NR	3 mo	Same as above	Same as above
8	Dâmaso(17) 2014	BRA	RCT	Aerobic training	55 (NR/NR)	Total: 16.89 ± 1.81	Obese post-pubertal adolescents aged 15–19 yr; BMI ≥95th percentile	AE	Aerobic training plus interdisciplinary therapy	3 d/wk; 60 min/session; 50–70% VO_2 / VT1	1 yr	BMI; body fat; lean mass; VAT; SAT; glucose; insulin; HOMA-IR; QUICKI; lipids; leptin; adiponectin	BOD POD: body composition; ultrasound: VAT/SAT thickness (cm); blood tests: metabolic and inflammatory markers
8	Dâmaso 2014	BRA	RCT	Aerobic plus resistance training	61 (NR/NR)	Total: 16.89 ± 1.81	Same as above	AE+RT	30 min aerobic plus 30 min resistance training; interdisciplinary therapy	3 d/wk; 60 min/session; AT at VT1; RT 3 sets, 6–20 RM	1 yr	Same as above	Same as above
9	Alberga(60) 2015	CAN	4-arm RCT	Aerobic training	75 (22/53)	15.5 ± 1.4	Inactive post-pubertal adolescents	AE[MV]	Aerobic exercise plus dietary counselling	4 d/wk; 20–40 min/session;	22 wk	BMI; waist; %BF; SAT; deep SAT;	MRI: body fat, VAT area at L4–L5 (cm²), SAT; blood tests:

							aged 14–18 yr; OW/OB; BMI ≥85th percentile with risk factor or ≥95th percentile			treadmill/cycle/elliptical; 70–85% HRmax		superficial SAT; VAT; ApoA-1; ApoB; ApoB/ApoA-1; HSCRP	ApoA-1, ApoB, HSCRP
9	Alberga 2015	CAN	4-arm RCT	Resistance training	78 (23/55)	15.9 ± 1.5	Same as above	RT	Whole-body resistance training plus dietary counselling	4 d/wk; 2–3 sets; 6–15 RM; resistance machines	22 wk	Same as above	Same as above
9	Alberga 2015	CAN	4-arm RCT	Combined training	75 (22/53)	15.5 ± 1.3	Same as above	AE+RT	Aerobic plus resistance training plus dietary counselling	4 d/wk; aerobic 20–40 min + resistance 2–3 sets/session	22 wk	Same as above	Same as above
9	Alberga 2015	CAN	4-arm RCT	Control	76 (24/52)	15.6 ± 1.3	Same as above	CON	Non-exercising control plus dietary counselling	NR	22 wk	Same as above	Same as above
10	Monteiro 2015(19)	BRA	3-arm partial RCT	Aerobic training	18 (10/8)	11.0 ± 1.0	Obese adolescents aged 11–17 yr; BMI obesity criteria	AE[V]	Walking/running aerobic training; no dietary intervention	3 d/wk; 50 min/session; 65–85% VO_2peak	20 wk	%BF; FM; FFM; android fat; waist; IAAT; SAT; hepatic steatosis; lipids; glucose; inflammatory markers	DXA: body composition; ultrasound: IAAT/SAT thickness (cm), hepatic steatosis; blood tests: lipids, glucose, cytokines
10	Monteiro 2015	BRA	3-arm partial RCT	Concurrent training	14 (9/5)	11.0 ± 1.3	Same as above	AE+RT	Aerobic plus resistance circuit training; no dietary intervention	3 d/wk; 60 min/session; 30 min AE + 30 min RT; 55–75% RM	20 wk	Same as above	Same as above

10	Monteiro 2015	BRA	3-arm partial RCT	Control	16 (8/8)	11.0 ± 1.9	Same as above	CON	No exercise intervention; usual lifestyle	NR	20 wk	Same as above	Same as above
11	Staiano(56) 2016	USA	RCT	Exergaming	22 (0/22)	15.3 ± 1.2	OW/OB post-menarcheal girls aged 14–18 yr; BMI ≥85th percentile	AE[MV]	Group-based dance exergaming using Kinect/Xbox 360; self-selected game and intensity	3 d/wk; 60 min/session; 36 h total	12 wk	BMI z-score; waist; %BF; regional fat; VAT; SAT; TAT; BMD; BP; lipids; glucose; insulin	Anthropometry: BMI/waist; DXA: body fat/BMD; MRI: VAT/SAT/TAT mass (kg); blood tests: lipids, glucose, insulin; BP measurement
11	Staiano 2016	USA	RCT	Control	19 (0/19)	16.1 ± 1.4	Same as above	CON	Self-directed care; maintain usual physical activity	NR	12 wk	Same as above	Same as above
12	da Silveira Campos(59) 2017	BRA	RCT	Aerobic training	39 (NR/NR)	Total: 16.0 ± 1.7	Post-pubertal obese adolescents; BMI >95th percentile; Tanner stage 5	AE[MV]	Treadmill aerobic training plus interdisciplinary therapy	3 d/wk; 60 min/session; HR at VT1	1 yr	BMI; fat mass; lean mass; VAT; SAT; glucose; insulin; HOMA-IR; HOMA-AD; QUICKI; adiponectin	BOD POD: body composition; ultrasound: VAT/SAT thickness (cm); blood tests: glucose, insulin, adiponectin
12	da Silveira Campos 2017	BRA	RCT	Aerobic plus resistance training	63 (NR/NR)	Total: 16.0 ± 1.7	Same as above	AE+RT	30 min aerobic plus 30 min resistance training; interdisciplinary therapy	3 d/wk; 60 min/session; RT 6–20 MR	1 yr	Same as above	Same as above
13	Dias 2017(18)	AUS/NOR	3-arm RCT	HIIT	33 (16/17)	12.4 ± 1.9	Children with obesity aged 7–16 yr	HIIT	HIIT plus nutrition advice	3 d/wk; 4 × 4 min at 85–95% HRmax; recovery 50–70% HRmax	12 wk	VO_2peak; VAT; SAT; TAAT; %BF; FM; FFM;	Treadmill test: VO_2peak; MRI: VAT/SAT/TAAT volume (cm^3);

											BMI; lipids; glucose; HbA1c; C-peptide; HOMA-IR; PA; diet	DXA/BOD POD: body composition; blood tests: biomarkers; accelerometer: PA; food records: diet
13	Dias 2017	AUS/NOR	3-arm RCT	MICT	32 (15/17)	11.9 ± 2.4	Same as above	AE[M]	Moderate-intensity continuous training plus nutrition advice	3 d/wk; 44 min/session; 60–70% HRmax	12 wk	Same as above	Same as above
13	Dias 2017	AUS/NOR	3-arm RCT	Nutrition	34 (15/19)	11.8 ± 2.4	Same as above	CON	Nutrition advice only	NR	12 wk	Same as above	Same as above
14	Liang 2018(66)	CHN	RCT	HIIT	9 (9/0)	NR	Male children with simple obesity; actual weight >20% above standard weight	HIIT	Treadmill HIIT with 60-s sprint and active recovery	3 d/wk; 60 s at 100% maximal speed + 3 min at 50% speed; 3–6 bouts/session	12 wk	Weight; BMI; body fat mass; %BF; VAT area; waist; SBP; DBP; Chemerin	Body composition analyzer: body composition/VAT area (cm²); tape: waist; sphygmomanometer: BP; ELISA: Chemerin
14	Liang 2018	CHN	RCT	MICT	9 (9/0)	NR	Same as above	AE	Continuous treadmill aerobic training	3 d/wk; 30–60 min/session; 80% HRpeak	12 wk	Same as above	Same as above
15	Lee 2020(57)	USA	3-arm RCT	Aerobic exercise	38 (13/25)	14.4 ± 1.6	OW/OB inactive adolescents aged 12–17 yr; BMI >85th percentile; Tanner II–V	AE[M]	Treadmill/elliptical aerobic exercise; no caloric restriction	3 d/wk; 40–60 min/session; 50–65% VO_2peak	6 mo	Weight; total/regional SAT; VAT; skeletal muscle; lipids; BP; cfPWV; cIMT	MRI: AT/SAT/VAT/SM (VAT mass, kg); blood tests: lipids; BP measurement; ultrasound: cIMT; Complior: cfPWV

15	Lee 2020	USA	3-arm RCT	Resistance exercise	40 (15/25)	14.4 ± 1.6	Same as above	RT	Whole-body machine-based resistance training; no caloric restriction	3 d/wk; ~60 min/session; 8 exercises; 2 sets; 12–15 reps	6 mo	Same as above	Same as above
15	Lee 2020	USA	3-arm RCT	Combined exercise	40 (14/26)	14.5 ± 1.7	Same as above	AE+RT	Aerobic exercise plus resistance training; no caloric restriction	3 d/wk; ~60 min/session; 30 min AE + 1 set RT exercises	6 mo	Same as above	Same as above
16	Cao 2022a(67)	CHN	3-arm RCT	HIIT	12 (12/0)	11.4 ± 0.8	Obese boys aged 11–13 yr; BMI ≥95th percentile; Tanner I–III	HIIT	School-based running HIIT; normal PE maintained	3 d/wk; 2 sets × 8 × 15 s at 90–100% MAS; 15 s recovery at 50% MAS	12 wk	BMI; %BF; FM; FFM; VAT; WC; VO_2peak; SBP; DBP; glucose; insulin; HOMA-IR; lipids	DXA: body composition/VAT mass (g); treadmill GXT: VO_2peak; 20-m SRT: MAS; blood tests: glucose, insulin, lipids
16	Cao 2022a	CHN	3-arm RCT	MICT	11 (11/0)	11.2 ± 0.7	Same as above	AE[M]	School-based continuous running; normal PE maintained	3 d/wk; 30 min/session; 60–70% MAS	12 wk	Same as above	Same as above
16	Cao 2022a	CHN	3-arm RCT	Control	13 (13/0)	11.0 ± 0.7	Same as above	CON	No exercise training; normal behavior and PE maintained	NR	12 wk	Same as above	Same as above
17	Cao 2022b(63)	CHN	RCT	Intervention group	20 (10/10)	11.2 ± 0.7	Obese children aged 10–13 yr; BMI ≥95th percentile	HIIT	School-based HIIT integrated into PE class	3 d/wk; 3 sets × 8 × 15 s at 100% MAS; 15 s recovery at 50% MAS	12 wk	BM; BMI; %BF; FM; FFM; VAT; VO_2max; MAS; HRrest; HRmax	BIA: body composition/VAT area (cm²); 20-m SRT: MAS/VO_2max; Polar HR monitor: HR

17	Cao 2022b	CHN	RCT	Control group	20 (10/10)	10.9 ± 0.4	Same as above	CON	Regular PE and usual school activity; no additional HIIT	NR	12 wk	Same as above	Same as above
18	Cao 2022c(62)	CHN	RCT	HIIT	20 (10/10)	11.2 ± 0.9	Obese children aged 10–13 yr; BMI obesity criteria	HIIT	School-based running HIIT; no dietary control	3 d/wk; 3 sets × 8 × 15 s at 100–120% MAS; 15 s recovery at 50% MAS	12 wk	BMI; %BF; FM; FFM; VAT; WC; VO_2max; BP; glucose; insulin; HOMA-IR; lipids	DXA: body composition/VAT mass (g); 20-m SRT: MAS/VO_2max; blood tests: glucose, insulin, lipids; BP/HR measurement
18	Cao 2022c	CHN	RCT	MICT	20 (10/10)	10.9 ± 0.8	Same as above	AE	School-based continuous running; no dietary control	3 d/wk; 20–40 min/session; 60–80% MAS	12 wk	Same as above	Same as above
18	Cao 2022c	CHN	RCT	Control	20 (10/10)	10.9 ± 0.9	Same as above	CON	No training control	NR	12 wk	Same as above	Same as above
19	Li 2023(61)	CHN	3-arm RCT	HIIT	16 (NR/NR)	Total: 11.0 ± 0.8	Obese children aged 10–13 yr; Chinese school-age OW/OB criteria	HIIT	School-based running HIIT; free diet	3 d/wk; 15 s at 100–120% MAS + 15 s recovery at 50% MAS; 3 sets × 8 bouts	12 wk	BMI; %BF; FM; FFM; WC; VAT; limb/trunk fat; VO_2max; HR; BP	DXA: body composition/VAT area (cm^2); 20-m SRT: VO_2max/MAS; Polar: HR; BP monitor: SBP/DBP
19	Li 2023	CHN	3-arm RCT	MICT	15 (NR/NR)	Total: 11.0 ± 0.8	Same as above	AE	School-based continuous running; free diet	3 d/wk; 20–40 min/session; 60–80% MAS	12 wk	Same as above	Same as above
19	Li 2023	CHN	3-arm RCT	Control	16 (NR/NR)	Total: 11.0 ± 0.8	Same as above	CON	No training control; usual school PE/activity maintained	NR	12 wk	Same as above	Same as above

20	Cao 2024(64)	CHN	3-arm RCT	HIIT	14 (7/7)	12.4 ± 0.4	Obese adolescents; Chinese school-age OW/OB criteria	HIIT	Running HIIT; affective response assessed during exercise	3 d/wk; 3 sets × 8 × 15 s at 100% MAS; 15 s recovery at 50% MAS; 18 min/session	12 wk	Weight; BMI; %BF; VAT; VO_2max; FS; FAS; RPE; PACES	DXA: %BF/VAT area (cm^2); GXT + K5: VO_2max/MAS; FS/FAS/RPE: affective response; PACES: enjoyment
20	Cao 2024	CHN	3-arm RCT	MIIT	14 (7/7)	12.1 ± 0.6	Same as above	AE[MV]	Moderate-intensity interval running; affective response assessed during exercise	3 d/wk; 3 sets × 8 × 15 s at 80% MAS; 15 s recovery at 50% MAS; 18 min/session	12 wk	Same as above	Same as above
20	Cao 2024	CHN	3-arm RCT	Control	14 (7/7)	12.4 ± 0.5	Same as above	CON	No additional exercise training beyond school PE/activity	NR	12 wk	Same as above	Same as above
21	Gao 2025(65)	CHN	3-arm single-blind RCT	CT1: SIT + stair climbing	10 (5/5)	Total: 10.1 ± 0.7	Obese children aged 9–13 yr; BMI ≥95th percentile; Tanner I–III	HIIT	Sprint interval training plus stair-climbing aerobic exercise	2 d/wk; 10 min SIT + 10 min stair climbing; stair climbing 60–70% HRmax	8 wk	BM; BMI; WC; %BF; FM; LBM; VAT; VO_2max; SBP; DBP; HRR	InBody770: body composition/VAT (g in Table 2; Methods describe visceral fat area in cm^2); tape: WC; 20-m SRT: VO_2max/MAS; BP monitor: BP; Polar: HR
21	Gao 2025	CHN	3-arm single-blind RCT	CT2: SIT + core strength training	10 (5/5)	Total: 10.1 ± 0.7	Same as above	HIIT+RT	Sprint interval training plus core strength exercises	2 d/wk; 10 min SIT + 10 min core strength; strength training 60–70% HRmax	8 wk	Same as above	Same as above
21	Gao 2025	CHN	3-arm single-blind RCT	Control	10 (5/5)	Total: 10.1 ± 0.7	Same as above	CON	Regular school PE/activity only; no additional exercise	NR	8 wk	Same as above	Same as above

Note. AE[M], moderate-intensity aerobic exercise; AE[MV], moderate-to-vigorous aerobic exercise; AE[V], vigorous-intensity aerobic exercise; AE+RT, aerobic exercise combined with resistance training; CON, control condition; HIIT, high-intensity interval training; HIIT+RT, high-intensity interval training combined with resistance training; RT, resistance training; VAT, visceral adipose tissue; SAT/SAAT/ASAT, subcutaneous abdominal adipose tissue; BMI, body mass index; %BF, body fat percentage; FM, fat mass; TFM, total fat mass; FFM/FFST, fat-free mass or fat-free soft tissue; BMC, bone mineral content; BMD, bone mineral density; AT, adipose tissue; IMCL, intramyocellular lipid; IMAT, intermuscular adipose tissue; MRI, magnetic resonance imaging; DXA, dual-energy X-ray absorptiometry; CT, computed tomography; MRS, magnetic resonance spectroscopy; OGTT, oral glucose tolerance test; HR, heart rate; HRmax, maximum heart rate; MET, metabolic equivalent; MVPA, moderate-to-vigorous physical activity; NR, not reported; PA, physical activity; RCT, randomized controlled trial. Studies are ordered chronologically by year of publication; studies published in the same year are ordered alphabetically by the first author's surname. VAT measurement units are reported as presented in the original studies.

1. Buchheit M, Laursen PB. High-intensity interval training, solutions to the programming puzzle: Part I: cardiopulmonary emphasis. *Sports Med* (2013) 43:313–338. doi: 10.1007/s40279-013-0029-x

2. Wang S, Zhou H, Zhao C, He H. Effect of exercise training on body composition and inflammatory cytokine levels in overweight and obese individuals: A systematic review and network meta-analysis. *Front Immunol* (2022) 13:921085. doi: 10.3389/fimmu.2022.921085

3. O'Donoghue G, Blake C, Cunningham C, Lennon O, Perrotta C. What exercise prescription is optimal to improve body composition and cardiorespiratory fitness in adults living with obesity? A network meta-analysis. *Obes Rev* (2021) 22:e13137. doi: 10.1111/obr.13137

4. Owens S, Gutin B, Allison J, Riggs S, Ferguson M, Litaker M, Thompson W. Effect of physical training on total and visceral fat in obese children. *Med Sci Sports Exercise* (1999) 31:143–148. doi: 10.1097/00005768-199901000-00022

5. Barbeau P, Johnson MH, Howe CA, Allison J, Davis CL, Gutin B, Lemmon CR. Ten months of exercise improves general and visceral adiposity, bone, and fitness in black girls. *Obesity (Silver Spring)* (2007) 15:2077–2085. doi: 10.1038/oby.2007.247

6. DAVIS JN, GYLLENHAMMER LE, VANNI AA, MEIJA M, TUNG A, SCHROEDER ET, SPRUIJT-METZ D, GORAN MI. Startup Circuit Training Program Reduces Metabolic Risk in Latino Adolescents. *Med Sci Sports Exercise* (2011) 43:2195–2203. doi: 10.1249/MSS.0b013e31821f5d4e

7. Saelens BE, Grow HM, Stark LJ, Seeley RJ, Roehrig H. Efficacy of increasing physical activity to reduce children's visceral fat: a pilot randomized controlled trial. *Int J Pediatr Obes* (2011) 6:102–112. doi: 10.3109/17477166.2010.482157

8. Davis CL, Pollock NK, Waller JL, Allison JD, Dennis BA, Bassali R, Meléndez A, Boyle CA, Gower BA. Exercise Dose and Diabetes Risk in Overweight and Obese Children: A Randomized Controlled Trial. *JAMA* (2012) 308:1103–1112. doi: 10.1001/2012.jama.10762

9. Lee S, Bacha F, Hannon T, Kuk JL, Boesch C, Arslanian S. Effects of Aerobic Versus Resistance Exercise Without Caloric Restriction on Abdominal Fat, Intrahepatic Lipid, and Insulin Sensitivity in Obese Adolescent Boys: A Randomized, Controlled Trial. *Diabetes* (2012) 61:2787–2795. doi: 10.2337/db12-0214

10. Lee S, Deldin AR, White D, Kim Y, Libman I, Rivera-Vega M, Kuk JL, Sandoval S, Boesch C, Arslanian S. Aerobic exercise but not resistance exercise reduces intrahepatic lipid content and visceral fat and improves insulin sensitivity in obese adolescent girls: a randomized controlled trial. *American Journal of Physiology-Endocrinology and Metabolism* (2013) 305:E1222–E1229. doi: 10.1152/ajpendo.00285.2013

11. Dâmaso AR, da Silveira Campos RM, Caranti DA, de Piano A, Fisberg M, Foschini D, de Lima Sanches P, Tock L, Lederman HM, Tufik S, et al. Aerobic plus resistance training was more effective in improving the visceral adiposity, metabolic profile and inflammatory markers than aerobic training in obese adolescents. *J Sports Sci* (2014) 32:1435–1445. doi: 10.1080/02640414.2014.900692

12. Alberga AS, Prud'homme D, Kenny GP, Goldfield GS, Hadjiyannakis S, Gougeon R, Phillips P, Malcolm J, Wells G, Doucette S, et al. Effects of aerobic and resistance training on abdominal fat, apolipoproteins and high-sensitivity C-reactive protein in adolescents with obesity: the HEARTY randomized clinical trial. *Int J Obes* (2015) 39:1494–1500. doi: 10.1038/ijo.2015.133

13. Monteiro PA, Chen KY, Lira FS, Saraiva BTC, Antunes BMM, Campos EZ, Freitas IF. Concurrent and aerobic exercise training promote similar benefits in body composition and metabolic profiles in obese adolescents. *Lipids Health Dis* (2015) 14:153. doi: 10.1186/s12944-015-0152-9

14. Staiano AE, Marker AM, Beyl RA, Hsia DS, Katzmarzyk PT, Newton RL. A randomized controlled trial of dance exergaming for exercise training in overweight and obese adolescent girls. *Pediatr Obes* (2017) 12:121–128. doi: 10.1111/ijpo.12117

15. da Silveira Campos RM, Landi Masquio DC, Campos Corgosinho F, de Lima Sanches P, de Piano A, Carnier J, Leão da Silva P, Grotti Clemente AP, de Castro Ferreira Vicente SE, Oyama LM, et al. Homeostasis model assessment-adiponectin: the role of different types of physical exercise in obese adolescents. *J Sports Med Phys Fitness* (2017) 57:831–838. doi: 10.23736/s0022-4707.16.06235-6

16. Dias KA, Ingul CB, Tjønna AE, Keating SE, Gomersall SR, Follestad T, Hosseini MS, Hollekim-Strand SM, Ro TB, Haram M, et al. Effect of High-Intensity Interval Training on Fitness, Fat Mass and Cardiometabolic Biomarkers in Children with Obesity: A Randomised Controlled Trial. *Sports Med* (2018) 48:733–746. doi: 10.1007/s40279-017-0777-0

17. Liang J, Hao L. Effects of high-intensity interval exercise on body composition, blood pressure, and serum chemerin in obese children. *Chin J Sch HEALTH* (2018) 39:1729–1732. doi: 10.16835/j.cnki.1000-9817.2018.11.039

18. Lee S, Libman I, Hughan KS, Kuk JL, Barinas-Mitchell E, Chung H, Arslanian S. Effects of exercise modality on body composition and cardiovascular disease risk factors in adolescents with obesity: a randomized clinical trial. *Appl Physiol Nutr Metab* (2020) 45:1377–1386. doi: 10.1139/apnm-2019-0993

19. Meng C, Yucheng T, Shu L, Yu Z. Effects of school-based high-intensity interval training on body composition, cardiorespiratory fitness and cardiometabolic markers in adolescent boys with obesity: a randomized controlled trial. *BMC Pediatr* (2022) 22:112. doi: 10.1186/s12887-021-03079-z

20. Cao M, Tang Y, Zou Y. Integrating High-Intensity Interval Training into a School Setting Improve Body Composition, Cardiorespiratory Fitness and Physical Activity in Children with Obesity: A Randomized Controlled Trial. *J Clin Med* (2022) 11:5436. doi: 10.3390/jcm11185436

21. Cao M, Tang Y, Li S, Zou Y. Effects of high-intensity interval training on body composition, cardiorespiratory fitness, and blood indicators in children with obesity. *Chin J Sports Med* (2022) 41:109–117. doi: 10.16038/j.1000-6710.2022.02.007

22. Li S, Cao M, Zou Y, Tang Y. Effects of high-intensity interval training on visceral fat and cardiorespiratory fitness in children with obesity. *J Phys Educ* (2023) 30:138–144. doi: 10.16237/j.cnki.cn44-1404/g8.20230324.002

23. Cao M, Yu F, Zou Y, Tang Y. Affective responses during interval training in adolescents with obesity: A randomized controlled trial. *Chin J Sports Med* (2024) 43:527–536. doi: 10.16038/j.1000-6710.2024.07.006

24. Gao Y, Wu M, Chen J, Feng Z, Wang X, Cao M. Effects of school-based concurrent training on body composition and cardiorespiratory fitness in obese children in a randomized controlled trial. *Sci Rep* (2025) 15:33300. doi: 10.1038/s41598-025-19041-3

Supplementary Table S2.

Supplementary Table S2. Definitions and measurement rules for primary and secondary outcomes

Outcome domain	Outcome indicator	Operational definition and eligible indices	Eligible measurement methods	Direction of effect	Decision rules for extraction
**Primary outcome**	**Visceral adipose tissue (VAT)**	VAT referred to fat located within the intra-abdominal or visceral compartment. Eligible indices included VAT area, VAT volume, visceral fat mass, intra-abdominal adipose tissue, abdominal visceral fat, or equivalent visceral fat indicators reported by the original studies.	Magnetic resonance imaging, computed tomography, dual-energy X-ray absorptiometry-derived regional estimates, or other validated body-composition methods explicitly used to quantify visceral or intra-abdominal fat.	Negative values favour exercise.	VAT or an equivalent visceral fat indicator was required for study inclusion. When multiple VAT indices were reported, the measure most directly representing visceral or intra-abdominal fat was prioritized. Immediate postintervention data were used for the primary synthesis.
**Secondary outcome**	**Subcutaneous adipose tissue (SAT)**	SAT referred to subcutaneous abdominal adipose tissue or regional subcutaneous fat measured outside the abdominal cavity. Eligible indices included SAT, SAAT, ASAT, abdominal subcutaneous fat, or equivalent subcutaneous abdominal adipose tissue indicators.	Magnetic resonance imaging, computed tomography, dual-energy X-ray absorptiometry-derived regional estimates, or other validated imaging or body-composition methods.	Negative values favour exercise.	SAT was extracted only from studies that met the mandatory VAT inclusion criterion. When both total and regional subcutaneous fat were reported, abdominal or regional SAT measures were prioritized.
**Secondary outcome**	**Body fat percentage**	Body fat percentage represented total body fat as a percentage of body mass. Eligible indices included %BF, percentage body fat, total body fat percentage, or equivalent whole-body adiposity percentage indicators.	Dual-energy X-ray absorptiometry, bioelectrical impedance analysis, skinfold-based estimation, air-displacement plethysmography, or other validated methods reported by the original studies.	Negative values favour exercise.	Whole-body percentage fat was prioritized. If several estimates were available, the primary or most valid method reported by the original study was used.
**Secondary outcome**	**Waist circumference**	Waist circumference represented an anthropometric proxy of abdominal adiposity measured around the waist or abdomen according to the protocol used in the original study.	Standard anthropometry using a measuring tape at a reported anatomical landmark, such as the umbilicus, midpoint between the lowest rib and iliac crest, or another study-specified waist site.	Negative values favour exercise.	Waist circumference was extracted as reported. When several waist measures were available, the prespecified primary waist measure or the measure most commonly used in the study was selected.
**Secondary outcome**	**Body mass index (BMI) or BMI z-score**	BMI-related outcomes included BMI, BMI z-score, BMI standard deviation score, or BMI percentile when used as a continuous adiposity indicator in children and adolescents.	Calculated from measured or reported body weight and height, or directly reported by the original study.	Negative values favour exercise.	BMI was prioritized when both BMI and BMI z-score were available for the same comparison. BMI z-score or equivalent age- and sex-standardized BMI measures were used when they were the main reported BMI-related outcome.
**Secondary outcome**	**Body weight**	Body weight represented total body mass measured in kilograms or another convertible weight unit.	Standard body-weight measurement using calibrated scales or equivalent procedures described by the original study.	Negative values favour exercise.	Postintervention or change-from-baseline values were extracted according to the effect-size computation rules. Units were harmonized where necessary before effect-size calculation.

**Secondary outcome**	**Fat mass**	Fat mass represented total or whole-body fat mass. Eligible indices included fat mass, total fat mass, total body fat mass, or equivalent absolute fat-mass indicators.	Dual-energy X-ray absorptiometry, bioelectrical impedance analysis, air-displacement plethysmography, or other validated body-composition methods.	Negative values favour exercise.	Total or whole-body fat mass was prioritized. Regional fat measures were not used for this domain unless they represented the study's total fat-mass outcome.
**Secondary outcome**	**Cardiorespiratory fitness**	Cardiorespiratory fitness referred to maximal or peak aerobic capacity. Eligible indices included VO_2max, VO_2peak, peak oxygen uptake, maximal oxygen consumption, or comparable aerobic fitness indicators.	Maximal or graded exercise tests on treadmill, cycle ergometer, shuttle-run protocols, or validated cardiopulmonary or field-based fitness assessments reported by the original studies.	Positive values favour exercise.	VO_2max was prioritized when available. VO_2peak or comparable peak aerobic fitness indicators were used when VO_2max was not reported.

Note. VAT, visceral adipose tissue; SAT, subcutaneous adipose tissue; SAAT, subcutaneous abdominal adipose tissue; ASAT, abdominal subcutaneous adipose tissue; BMI, body mass index; %BF, body fat percentage; VO_2max, maximal oxygen uptake; VO_2peak, peak oxygen uptake. Negative values favour exercise for adiposity-related outcomes, whereas positive values favour exercise for cardiorespiratory fitness outcomes. Outcomes other than VAT were extracted only from studies that met the mandatory VAT or equivalent visceral fat inclusion criterion.

Supplementary Table S3.

Supplementary Table S3. Risk-of-bias assessment of the included studies using the Cochrane risk-of-bias tool

ID	Study	D1	D2	D3	D4	D5	D6	Overall

1	Alberga 2015	Low	Low	Low	Unclear	Low	Unclear	**Unclear**
2	Barbeau 2007	Unclear	Unclear	Low	Unclear	Low	Unclear	**Unclear**
3	Cao 2022a	Low	Low	Low	High	Low	Unclear	**High**
4	Cao 2022b	Low	Low	Low	High	Low	Unclear	**High**
5	Cao 2022c	Low	Low	Low	Low	Low	Unclear	**Unclear**
6	Cao 2024	Low	Low	Low	Low	Low	Low	**Low**
7	Damaso 2014	Low	Low	Unclear	Low	Low	High	**High**
8	Da Silveira Campos 2017	Low	Low	Low	Low	Low	High	**High**
9	Davis 2011	Low	Low	Low	Low	Low	Low	**Low**
10	Davis 2012	Low	Low	Low	Low	Low	Low	**Low**
11	Dias 2017	Low	Low	Low	High	Low	Low	**High**
12	Gao 2025	Low	Unclear	Low	High	Low	High	**High**
13	Lee 2012	Low	Low	Low	Low	Low	Low	**Low**
14	Lee 2013	Low	Unclear	Low	High	Low	Unclear	**High**
15	Li 2023	Low	Unclear	Low	High	Low	High	**High**
16	Liang 2018	Low	Low	Low	Low	Low	High	**High**
17	Monteiro 2015	High	High	Low	High	Low	High	**High**
18	Owens 1999	Low	Low	Low	Low	Low	Low	**Low**
19	Saelens 2011	Low	Low	Low	Low	Low	High	**High**
20	SoJung 2020	Low	Low	Low	Low	Low	Unclear	**Unclear**
21	Staiano 2016	Low	Unclear	Low	Low	Low	Unclear	**Unclear**

Risk-of-bias summary

Domain	Low	Unclear	High	Total
D1 Random sequence generation (selection bias)	19	1	1	21
D2 Allocation concealment (selection bias)	15	5	1	21
D3 Blinding of outcome assessment (detection bias)	20	1	0	21
D4 Incomplete outcome data (attrition bias)	12	2	7	21
D5 Selective reporting (reporting bias)	21	0	0	21
D6 Other bias	6	8	7	21
Overall risk of bias	5	5	11	21

Note. D1, random sequence generation; D2, allocation concealment; D3, blinding of outcome assessment; D4, incomplete outcome data; D5, selective reporting; D6, other bias; Overall, overall risk of bias. Risk of bias was assessed using the Cochrane risk-of-bias tool and classified as Low, Unclear, or High. The overall judgement was classified as High when at least one domain was rated as high risk, Low when all domains were rated as low risk, and Unclear otherwise.

Supplementary Table S4.

Supplementary Table S4. Main effects, heterogeneity, prediction intervals, and robust variance estimation results for primary and secondary outcomes

Outcome	Studies	k	Participants	Hedges' g	95% CI	p value	95% PI	Q(df)	Q p value	Total I^2 (%)	Level 2 I^2 (%)	Level 3 I^2 (%)	RVE estimate	RVE 95% CI	RVE p value
**VAT**	21	32	1441	-0.725	[-1.120, -0.330]	<0.001	[-2.399, 0.948]	159.013 (31)	<0.001	89.71	0.00	89.71	-0.725	[-1.129, -0.322]	0.001
**SAT**	14	20	1171	-0.256	[-0.504, -0.007]	0.044	[-1.000, 0.489]	56.776 (19)	<0.001	68.61	0.00	68.61	-0.256	[-0.512, 0.0003]	0.050
**BF%**	18	26	1206	-0.926	[-1.262, -0.590]	<0.001	[-2.188, 0.336]	100.276 (25)	<0.001	81.60	0.00	81.60	-0.926	[-1.271, -0.582]	<0.001
**WC**	12	17	581	-0.692	[-0.984, -0.400]	<0.001	[-1.547, 0.163]	41.172 (16)	<0.001	58.36	56.21	2.15	-0.692	[-0.994, -0.390]	<0.001
**BMI**	17	25	1151	-1.010	[-1.580, -0.440]	0.001	[-3.232, 1.212]	202.403 (24)	<0.001	93.12	6.14	86.97	-1.010	[-1.595, -0.425]	0.002
**BW**	14	20	790	-0.783	[-1.432, -0.135]	0.021	[-3.047, 1.480]	160.610 (19)	<0.001	92.39	0.00	92.39	-0.783	[-1.453, -0.114]	0.025
**FM**	16	23	1027	-1.205	[-1.905, -0.506]	0.002	[-3.850, 1.440]	232.675 (22)	<0.001	94.54	0.00	94.54	-1.205	[-1.924, -0.486]	0.003
**VO_2max**	13	20	898	1.316	[0.667, 1.965]	<0.001	[-0.893, 3.526]	182.512 (19)	<0.001	92.63	9.60	83.03	1.316	[0.640, 1.992]	0.001

Note. VAT, visceral adipose tissue; SAT, subcutaneous adipose tissue; BF%, body fat percentage; WC, waist circumference; BMI, body mass index; BW, body weight; FM, fat mass; VO_2max, maximal oxygen uptake; CI, confidence interval; PI, prediction interval; RVE, robust variance estimation; k, number of effect sizes. Participants indicate the approximate number of participants contributing to each outcome. Negative values favour exercise for adiposity-related outcomes, whereas positive values favour exercise for VO_2max. Level 2 I^2 represents within-study heterogeneity, and Level 3 I^2 represents between-study heterogeneity in the three-level model.

Subcutaneous Adipose Tissue. The SAT outcome included 14 studies contributing 20 effect sizes. The three-level random-effects model showed that exercise interventions significantly reduced SAT in children and adolescents with overweight or obesity compared with control conditions, although the effect size was small (Hedges' g = −0.256, 95% CI [−0.504, −0.007], p = 0.044). The 95% prediction interval was [−1.000, 0.489], crossing the null and indicating uncertainty in the true effect that might be observed in future comparable studies. Heterogeneity was statistically significant and moderate-to-high, $Q(19) = 56.776$, $p < 0.001$, $I^2 = 68.61\%$, and was mainly attributable to between-study variation (Level 3 $I^2 = 68.61\%$; Level 2 $I^2 \approx 0$). Robust variance estimation clustered at the study level was consistent in direction with the main model, but the statistical significance was borderline (g = −0.256, 95% CI [−0.512, 0.0003], p = 0.050). Overall, exercise interventions may have a small beneficial effect on SAT, but this finding should be interpreted cautiously in light of the prediction interval and the degree of heterogeneity (Table 2; Supplementary Table S4; Supplementary Figure S2).

Body Fat Percentage. The body fat percentage outcome included 18 studies contributing 26 effect sizes. The three-level random-effects model showed that exercise interventions significantly reduced body fat percentage in children and adolescents with overweight or obesity compared with control conditions, with a large effect size (Hedges' $g = -0.926$, 95% CI [−1.262, −0.590], $p < 0.001$). The 95% prediction interval was [−2.188, 0.336], crossing the null and suggesting that the true effect in future comparable studies may vary according to study context. Heterogeneity was statistically significant and substantial, $Q(25) = 100.276$, $p < 0.001$, $I^2 = 81.60\%$, and was mainly attributable to between-study variation (Level 3 $I^2 = 81.60\%$; Level 2 $I^2 \approx 0$). Robust variance estimation clustered at the study level was consistent with the main model, with the pooled effect remaining statistically significant ($g = -0.926$, 95% CI [−1.271, −0.582], $p < 0.001$), supporting the robustness of the main finding for body fat percentage. Overall, exercise interventions showed a clear average reduction in body fat percentage, although the effect magnitude varied across studies (Table 2; Supplementary Table S4; Supplementary Figure S2).

Waist Circumference. The waist circumference outcome included 12 studies contributing 17 effect sizes. The three-level random-effects model showed that exercise interventions significantly reduced waist circumference in children and adolescents with overweight or obesity compared with control conditions, with a moderate effect size (Hedges' $g = -0.692$, 95% CI [−0.984, −0.400], $p < 0.001$). The 95% prediction interval was [−1.547, 0.163], crossing the null and indicating that the true effect in future comparable studies may still vary. Heterogeneity was statistically significant, $Q(16) = 41.172$, $p < 0.001$, $I^2 = 58.36\%$, and was mainly attributable to within-study variation among effect sizes (Level 2 $I^2 = 56.21\%$; Level 3 $I^2 = 2.15\%$). Robust variance estimation clustered at the study level was consistent with the main model, with the pooled effect remaining statistically significant ($g = -0.692$, 95% CI [−0.994, −0.390], $p < 0.001$), supporting the robustness of the main finding for waist circumference. Overall, exercise interventions reduced waist circumference on average, and the RVE results supported the robustness of this effect (Table 2; Supplementary Table S4; Supplementary Figure S2).

Body Mass Index. The BMI outcome included 17 studies contributing 25 effect sizes. The three-level random-effects model showed that exercise interventions significantly reduced BMI in children and adolescents with overweight or obesity compared with control conditions, with a large effect size (Hedges' $g = -1.010$, 95% CI [−1.580, −0.440], $p = 0.001$). The 95% prediction interval was [−3.232, 1.212], crossing the null and suggesting considerable uncertainty in the true effect that might be observed in future comparable studies. Heterogeneity was statistically significant and substantial, $Q(24) = 202.403$, $p < 0.001$, $I^2 = 93.12\%$, and was mainly attributable to between-study variation (Level 3 $I^2 = 86.97\%$; Level 2 $I^2 = 6.14\%$). Robust variance estimation clustered at the study level was consistent with the main model, with the pooled effect remaining statistically significant ($g = -1.010$, 95% CI [−1.595, −0.425], $p = 0.002$), supporting the robustness of the main finding for BMI. Overall, exercise interventions reduced BMI on average in children and adolescents with overweight or obesity, but the wide prediction interval and high heterogeneity indicate that the magnitude of the effect may differ across study contexts, intervention approaches, and participant characteristics (Table 2; Supplementary Table S4; Supplementary Figure S2).

Body Weight. The body weight outcome included 14 studies contributing 20 effect sizes. The three-level random-effects model showed that exercise interventions significantly reduced body weight in children and adolescents with overweight or obesity compared with control conditions, with a moderate effect size (Hedges' $g = -0.783$, 95% CI [−1.432, −0.135], $p = 0.021$). The 95% prediction interval was [−3.047, 1.480], crossing the null and indicating substantial uncertainty in the true effect that might be observed in future comparable studies. Heterogeneity was statistically significant and substantial, $Q(19) = 160.610$, $p < 0.001$, $I^2 = 92.39\%$, and was mainly attributable to between-study variation (Level 3 $I^2 = 92.39\%$; Level 2 $I^2 \approx 0$). Robust variance estimation clustered at the study level was consistent in direction with the main model, and the pooled effect remained statistically significant ($g = -0.783$, 95% CI

[−1.453, −0.114], $p = 0.025$), supporting the robustness of the main finding for body weight. Overall, exercise interventions reduced body weight on average in children and adolescents with overweight or obesity, but the wide prediction interval and high heterogeneity suggest that the magnitude of the effect may differ substantially across study contexts, intervention approaches, and participant characteristics (Table 2; Supplementary Table S4; Supplementary Figure S2).

Fat Mass. The fat mass outcome included 16 studies contributing 23 effect sizes. The three-level random-effects model showed that exercise interventions significantly reduced fat mass in children and adolescents with overweight or obesity compared with control conditions, with a large effect size (Hedges' $g = -1.205$, 95% CI [−1.905, −0.506], $p = 0.002$). The 95% prediction interval was [−3.850, 1.440], crossing the null and suggesting substantial variability in the true effect that might be observed in future comparable studies. Heterogeneity was statistically significant and substantial, $Q(22) = 232.675$, $p < 0.001$, $I^2 = 94.54\%$, and was mainly attributable to between-study variation (Level 3 $I^2 = 94.54\%$; Level 2 $I^2 \approx 0$). Robust variance estimation clustered at the study level was consistent with the main model, with the pooled effect remaining statistically significant ($g = -1.205$, 95% CI [−1.924, −0.486], $p = 0.003$), supporting the robustness of the main finding for fat mass. Overall, exercise interventions showed a significant average reduction in fat mass, and this effect remained stable after RVE correction, although the wide prediction interval and substantial heterogeneity should be considered when interpreting its generalizability (Table 2; Supplementary Table S4; Supplementary Figure S2).

Cardiorespiratory Fitness: VO_2max. The VO_2max outcome included 13 studies contributing 20 effect sizes. The three-level random-effects model showed that exercise interventions significantly improved VO_2max in children and adolescents with overweight or obesity compared with control conditions, with a large effect size (Hedges' $g = 1.316$, 95% CI [0.667, 1.965], $p < 0.001$). Positive values indicated effects favouring exercise for cardiorespiratory fitness. The 95% prediction interval was [−0.893, 3.526], crossing the null and suggesting that the true effect in future comparable studies may still vary substantially. Heterogeneity was statistically significant and substantial, $Q(19) = 182.512$, $p < 0.001$, $I^2 = 92.63\%$, and was mainly attributable to between-study variation (Level 3 $I^2 = 83.03\%$; Level 2 $I^2 = 9.60\%$). Robust variance estimation clustered at the study level was consistent with the main model, with the pooled effect remaining statistically significant ($g = 1.316$, 95% CI [0.640, 1.992], $p = 0.001$), supporting the robustness of the main finding for VO_2max. Overall, exercise interventions showed a clear average improvement in cardiorespiratory fitness, although the prediction interval and high heterogeneity indicate that the magnitude of this improvement may differ across study contexts (Table 2; Supplementary Table S4; Supplementary Figure S2).

Supplementary Table S5.

**Supplementary Table S5. Subgroup analyses by exercise modality, age group, and overall risk of bias for primary and secondary outcomes**

Outcome	Moderator	Subgroup	Studies	k	Hedges' g	95% CI	P value	95% PI	I² (%)	P subgroup
**VAT**	**Exercise modality**	**AE[M]**	2	2	-0.918	[-1.781, -0.054]	0.038	[-2.597, 0.762]	47.89	0.116
		AE[MV]	8	8	-0.466	[-0.910, -0.022]	0.040	[-1.974, 1.042]	80.29	
		AE[V]	3	4	-0.676	[-1.422, 0.070]	0.074	[-2.298, 0.946]	NA	
		AE+RT	6	6	-0.438	[-0.910, 0.033]	0.067	[-1.954, 1.078]	0.00	
		HIIT	8	8	-1.332	[-1.874, -0.789]	<0.001	[-2.871, 0.208]	89.59	
		HIIT+RT	1	1	-1.437	[-3.138, 0.264]	0.094	[-3.666, 0.792]	NA	
		RT	3	3	-0.436	[-0.961, 0.088]	0.099	[-1.970, 1.097]	0.00	
	Age group	<12 years	11	17	-1.002	[-1.489, -0.515]	<0.001	[-2.667, 0.663]	92.50	0.082
		>=12 years	10	14	-0.446	[-0.949, 0.057]	0.080	[-2.116, 1.224]	41.67	
	Overall risk of bias	Low	5	7	-0.629	[-1.444, 0.187]	0.126	[-2.526, 1.269]	53.69	0.372
		Unclear	5	9	-0.310	[-1.104, 0.484]	0.431	[-2.199, 1.578]	72.99	
		High	11	16	-0.977	[-1.535, -0.418]	0.001	[-2.779, 0.825]	90.91	
**SAT**	**Exercise modality**	**AE[M]**	1	1	-0.030	[-1.219, 1.158]	0.957	[-1.486, 1.426]	NA	0.972
		AE[MV]	4	4	-0.224	[-0.674, 0.225]	0.302	[-1.178, 0.729]	72.43	
		AE[V]	4	5	-0.406	[-0.878, 0.067]	0.087	[-1.370, 0.559]	68.15	
		AE+RT	6	6	-0.206	[-0.576, 0.164]	0.253	[-1.125, 0.713]	71.87	
		HIIT	1	1	-0.035	[-1.252, 1.181]	0.951	[-1.514, 1.443]	NA	
		RT	3	3	-0.213	[-0.665, 0.238]	0.328	[-1.168, 0.741]	77.58	
	Age group	<12 years	6	8	-0.186	[-0.577, 0.205]	0.332	[-1.077, 0.705]	71.13	0.622

		>=12 years	9	12	-0.304	[-0.634, 0.025]	0.068	[-1.171, 0.562]	69.43	
	Overall risk of bias	Low	4	5	-0.736	[-1.157, -0.315]	0.002	[-1.471, -0.001]	7.78	0.040
		Unclear	4	7	-0.086	[-0.453, 0.282]	0.629	[-0.792, 0.621]	76.89	
		High	6	8	-0.088	[-0.427, 0.252]	0.594	[-0.780, 0.605]	36.45	
**BF%**	**Exercise modality**	**AE[M]**	2	2	-1.244	[-2.119, -0.368]	0.008	[-2.897, 0.409]	92.06	0.638
		AE[MV]	6	6	-1.267	[-1.877, -0.658]	<0.001	[-2.796, 0.262]	87.69	
		AE[V]	4	5	-0.704	[-1.395, -0.014]	0.046	[-2.267, 0.858]	0.00	
		AE+RT	3	3	-0.536	[-1.316, 0.244]	0.166	[-2.140, 1.068]	41.56	
		HIIT	8	8	-0.980	[-1.529, -0.431]	0.001	[-2.486, 0.526]	63.62	
		HIIT+RT	1	1	-1.641	[-3.182, -0.099]	0.038	[-3.725, 0.443]	NA	
		RT	1	1	-0.600	[-2.223, 1.023]	0.448	[-2.744, 1.544]	NA	
	Age group	<12 years	11	17	-1.035	[-1.450, -0.620]	<0.001	[-2.405, 0.336]	87.24	0.359
		>=12 years	7	8	-0.765	[-1.274, -0.256]	0.005	[-2.167, 0.637]	60.32	
	Overall risk of bias	Low	4	6	-0.903	[-1.654, -0.151]	0.021	[-2.485, 0.679]	67.54	0.902
		Unclear	3	4	-0.774	[-1.633, 0.086]	0.075	[-2.410, 0.862]	81.65	
		High	11	16	-0.985	[-1.452, -0.518]	<0.001	[-2.453, 0.483]	82.76	
**WC**	**Exercise modality**	**AE[M]**	1	1	-0.429	[-1.621, 0.762]	0.441	[-1.839, 0.980]	NA	0.184
		AE[MV]	4	4	-0.468	[-1.021, 0.086]	0.089	[-1.402, 0.467]	77.61	
		AE[V]	2	2	-0.358	[-1.014, 0.297]	0.251	[-1.357, 0.640]	64.63	
		AE+RT	2	2	-0.594	[-1.405, 0.216]	0.133	[-1.701, 0.512]	0.00	

		HIIT	5	5	-0.928	[-1.464, -0.391]	0.003	[-1.852, -0.003]	0.00	
		HIIT+RT	1	1	-2.644	[-4.202, -1.086]	0.004	[-4.375, -0.913]	NA	
		RT	2	2	-0.697	[-1.515, 0.120]	0.086	[-1.809, 0.414]	61.61	
	Age group	<12 years	7	12	-0.752	[-1.114, -0.391]	<0.001	[-1.770, 0.265]	65.79	0.428
		>=12 years	4	4	-0.477	[-1.102, 0.147]	0.123	[-1.615, 0.660]	44.29	
	Overall risk of bias	Low	2	2	-0.873	[-1.711, -0.036]	0.042	[-2.040, 0.293]	0.65	0.281
		Unclear	3	4	-0.342	[-0.861, 0.177]	0.179	[-1.305, 0.621]	51.57	
		High	7	11	-0.813	[-1.186, -0.440]	<0.001	[-1.706, 0.080]	54.13	
**BMI**	**Exercise modality**	**AE[M]**	2	2	-1.297	[-2.807, 0.213]	0.088	[-4.121, 1.527]	93.57	0.714
		AE[MV]	7	7	-1.263	[-2.185, -0.341]	0.010	[-3.822, 1.296]	95.69	
		AE[V]	3	4	-0.465	[-1.759, 0.829]	0.461	[-3.180, 2.250]	66.15	
		AE+RT	3	3	-0.298	[-1.632, 1.036]	0.646	[-3.032, 2.436]	28.06	
		HIIT	8	8	-1.456	[-2.327, -0.585]	0.002	[-3.997, 1.085]	84.66	
		HIIT+RT	1	1	-1.591	[-3.893, 0.711]	0.164	[-4.907, 1.725]	NA	
	Age group	<12 years	10	16	-1.315	[-2.005, -0.625]	<0.001	[-3.675, 1.046]	95.06	0.177
		>=12 years	7	8	-0.683	[-1.489, 0.123]	0.093	[-3.080, 1.714]	74.89	
	Overall risk of bias	Low	3	5	-0.902	[-2.340, 0.535]	0.206	[-3.737, 1.932]	86.84	0.880
		Unclear	3	4	-0.747	[-2.186, 0.691]	0.293	[-3.582, 2.088]	93.05	
		High	11	16	-1.120	[-1.883, -0.357]	0.006	[-3.679, 1.440]	92.45	
**BW**	**Exercise modality**	**AE[M]**	1	1	-1.027	[-2.783, 0.728]	0.228	[-4.004, 1.950]	NA	0.813

		AE[MV]	5	5	-1.088	[-2.147, -0.029]	0.045	[-3.715, 1.539]	95.53	
		AE[V]	1	1	-0.181	[-2.073, 1.712]	0.840	[-3.240, 2.879]	NA	
		AE+RT	4	4	-0.093	[-1.266, 1.080]	0.867	[-2.768, 2.582]	77.53	
		HIIT	6	6	-1.197	[-2.190, -0.204]	0.022	[-3.798, 1.404]	89.32	
		HIIT+RT	1	1	-1.598	[-3.877, 0.681]	0.154	[-4.910, 1.714]	NA	
		RT	2	2	-0.355	[-1.827, 1.117]	0.611	[-3.174, 2.463]	88.92	
	Age group	<12 years	7	10	-1.010	[-1.765, -0.254]	0.012	[-3.420, 1.400]	93.34	0.271
		>=12 years	7	9	-0.621	[-1.371, 0.129]	0.099	[-3.029, 1.787]	74.68	
	Overall risk of bias	Low	3	4	-0.751	[-2.187, 0.685]	0.285	[-3.549, 2.047]	NA	0.477
		Unclear	1	2	0.564	[-1.863, 2.991]	0.630	[-2.850, 3.978]	NA	
		High	10	14	-0.939	[-1.733, -0.146]	0.023	[-3.468, 1.590]	93.36	
**FM**	**Exercise modality**	**AE[M]**	2	2	-1.712	[-2.868, -0.556]	0.006	[-4.458, 1.034]	90.89	0.416
		AE[MV]	6	6	-1.442	[-2.381, -0.504]	0.005	[-4.104, 1.219]	94.02	
		AE[V]	2	2	-0.367	[-1.697, 0.963]	0.567	[-3.191, 2.457]	37.77	
		AE+RT	4	4	-0.234	[-1.407, 0.939]	0.678	[-2.987, 2.519]	81.75	
		HIIT	7	7	-1.932	[-2.847, -1.017]	<0.001	[-4.586, 0.721]	91.31	
		HIIT+RT	1	1	-1.237	[-3.282, 0.808]	0.218	[-4.460, 1.985]	NA	
		RT	1	1	-0.251	[-1.575, 1.074]	0.694	[-3.071, 2.570]	NA	
	Age group	<12 years	9	14	-1.430	[-2.214, -0.647]	0.001	[-4.158, 1.297]	94.83	0.250
		>=12 years	7	8	-0.964	[-1.800, -0.129]	0.026	[-3.707, 1.778]	77.24	

	Overall risk of bias	Low	2	2	-0.970	[-2.986, 1.046]	0.328	[-4.392, 2.452]	33.19	0.434
		Unclear	4	6	-0.508	[-1.914, 0.898]	0.460	[-3.610, 2.594]	94.42	
		High	10	15	-1.545	[-2.453, -0.638]	0.002	[-4.456, 1.365]	94.07	
**VO_2max**	**Exercise modality**	**AE[M]**	2	2	1.468	[0.440, 2.497]	0.010	[-0.701, 3.638]	81.16	0.088
		AE[MV]	2	2	1.467	[0.330, 2.603]	0.016	[-0.756, 3.689]	87.33	
		AE[V]	1	2	0.368	[-1.560, 2.295]	0.680	[-2.346, 3.082]	0.00	
		AE+RT	1	1	0.541	[-1.567, 2.649]	0.580	[-2.304, 3.386]	NA	
		HIIT	7	7	2.351	[1.522, 3.180]	<0.001	[0.269, 4.434]	75.60	
		HIIT+RT	1	1	1.840	[-0.044, 3.724]	0.055	[-0.843, 4.523]	NA	
		RT	2	2	1.219	[-0.287, 2.725]	0.101	[-1.213, 3.651]	84.20	
	Age group	<12 years	9	14	1.221	[0.473, 1.968]	0.003	[-1.243, 3.684]	95.09	0.550
		>=12 years	5	6	1.506	[0.567, 2.446]	0.003	[-1.022, 4.034]	77.44	
	Overall risk of bias	Low	5	7	0.923	[-0.146, 1.991]	0.086	[-1.624, 3.470]	94.20	0.428
		Unclear	2	3	0.959	[-0.691, 2.608]	0.237	[-1.882, 3.799]	93.70	
		High	6	10	1.774	[0.794, 2.753]	0.001	[-0.737, 4.285]	86.89	

Note. VAT, visceral adipose tissue; SAT, subcutaneous adipose tissue; BF%, body fat percentage; WC, waist circumference; BMI, body mass index; BW, body weight; FM, fat mass; VO_2max, maximal oxygen uptake; AE[M], moderate-intensity aerobic exercise; AE[MV], moderate-to-vigorous aerobic exercise; AE[V], vigorous-intensity aerobic exercise; AE+RT, aerobic exercise combined with resistance training; HIIT, high-intensity interval training; HIIT+RT, high-intensity interval training combined with resistance training; RT, resistance training; CI, confidence interval; PI, prediction interval; k, number of effect sizes. Negative values favour exercise for adiposity-related outcomes, whereas positive values favour exercise for VO_2max. I^2 indicates total heterogeneity within each subgroup. P subgroup indicates the between-subgroup test for each moderator within each outcome. NA indicates that I^2 was not estimable, usually because the subgroup contained a single effect size or model variance components were not available.

Subcutaneous Adipose Tissue. The SAT outcome included 14 studies contributing 20 effect sizes. The three-level random-effects model showed that exercise interventions significantly reduced SAT in children and adolescents with overweight or obesity compared with control conditions, although the effect size was

small (Hedges' $g = −0.256$, 95% CI [−0.504, −0.007], $p = 0.044$) (Supplementary Table S4) . The 95% prediction interval was [−1.000, 0.489], crossing the null and indicating uncertainty in the true effect that might be observed in future comparable studies. Heterogeneity was statistically significant and moderate-to-high, $Q(19) = 56.776$, $p < 0.001$, $I^2 = 68.61\%$, and was mainly attributable to between-study variation (Level 3 $I^2 = 68.61\%$; Level 2 $I^2 \approx 0$) (Supplementary Table S4) . Robust variance estimation clustered at the study level was consistent in direction with the main model, but the statistical significance was borderline ($g = −0.256$, 95% CI [−0.512, 0.0003], $p = 0.050$). Overall, exercise interventions may have a small beneficial effect on SAT, but this finding should be interpreted cautiously in light of the prediction interval and the degree of heterogeneity (Supplementary Figure S2) .

Body Fat Percentage. The body fat percentage outcome included 18 studies contributing 26 effect sizes. The three-level random-effects model showed that exercise interventions significantly reduced body fat percentage in children and adolescents with overweight or obesity compared with control conditions, with a large effect size (Hedges' $g = −0.926$, 95% CI [−1.262, −0.590], $p < 0.001$) (Supplementary Table S4) . The 95% prediction interval was [−2.188, 0.336], crossing the null and suggesting that the true effect in future comparable studies may vary according to study context. Heterogeneity was statistically significant and substantial, $Q(25) = 100.276$, $p < 0.001$, $I^2 = 81.60\%$, and was mainly attributable to between-study variation (Level 3 $I^2 = 81.60\%$; Level 2 $I^2 \approx 0$) (Supplementary Table S4) . Robust variance estimation clustered at the study level was consistent with the main model, with the pooled effect remaining statistically significant ($g = −0.926$, 95% CI [−1.271, −0.582], $p < 0.001$), supporting the robustness of the main finding for body fat percentage. Overall, exercise interventions showed a clear average reduction in body fat percentage, although the effect magnitude varied across studies (Supplementary Figure S2) .

Waist Circumference. The waist circumference outcome included 12 studies contributing 17 effect sizes. The three-level random-effects model showed that exercise interventions significantly reduced waist circumference in children and adolescents with overweight or obesity compared with control conditions, with a moderate effect size (Hedges' $g = −0.692$, 95% CI [−0.984, −0.400], $p < 0.001$) (Supplementary Table S4) . The 95% prediction interval was [−1.547, 0.163], crossing the null and indicating that the true effect in future comparable studies may still vary. Heterogeneity was statistically significant, $Q(16) = 41.172$, $p < 0.001$, $I^2 = 58.36\%$, and was mainly attributable to within-study variation among effect sizes (Level 2 $I^2 = 56.21\%$; Level 3 $I^2 = 2.15\%$) (Supplementary Table S4) . Robust variance estimation clustered at the study level was consistent with the main model, with the pooled effect remaining statistically significant ($g = −0.692$, 95% CI [−0.994, −0.390], $p < 0.001$), supporting the robustness of the main finding for waist circumference. Overall, exercise interventions reduced waist circumference on average, and the RVE results supported the robustness of this effect (Supplementary Figure S2) .

Body Mass Index. The BMI outcome included 17 studies contributing 25 effect sizes. The three-level random-effects model showed that exercise interventions significantly reduced BMI in children and adolescents with overweight or obesity compared with control conditions, with a large effect size (Hedges' g =

−1.010, 95% CI [−1.580, −0.440], $p = 0.001$) (Supplementary Table S4) . The 95% prediction interval was [−3.232, 1.212], crossing the null and suggesting considerable uncertainty in the true effect that might be observed in future comparable studies. Heterogeneity was statistically significant and substantial, $Q(24) = 202.403$, $p < 0.001$, $I^2 = 93.12\%$, and was mainly attributable to between-study variation (Level 3 $I^2 = 86.97\%$; Level 2 $I^2 = 6.14\%$) (Supplementary Table S4) . Robust variance estimation clustered at the study level was consistent with the main model, with the pooled effect remaining statistically significant (g = −1.010, 95% CI [−1.595, −0.425], $p = 0.002$), supporting the robustness of the main finding for BMI. Overall, exercise interventions reduced BMI on average in children and adolescents with overweight or obesity, but the wide prediction interval and high heterogeneity indicate that the magnitude of the effect may differ across study contexts, intervention approaches, and participant characteristics (Supplementary Figure S2) .

Body Weight. The body weight outcome included 14 studies contributing 20 effect sizes. The three-level random-effects model showed that exercise interventions significantly reduced body weight in children and adolescents with overweight or obesity compared with control conditions, with a moderate effect size (Hedges' g = −0.783, 95% CI [−1.432, −0.135], $p = 0.021$) (Supplementary Table S4) . The 95% prediction interval was [−3.047, 1.480], crossing the null and indicating substantial uncertainty in the true effect that might be observed in future comparable studies. Heterogeneity was statistically significant and substantial, $Q(19) = 160.610$, $p < 0.001$, $I^2 = 92.39\%$, and was mainly attributable to between-study variation (Level 3 $I^2 = 92.39\%$; Level 2 $I^2 \approx 0$) (Supplementary Table S4) . Robust variance estimation clustered at the study level was consistent in direction with the main model, and the pooled effect remained statistically significant (g = −0.783, 95% CI [−1.453, −0.114], $p = 0.025$), supporting the robustness of the main finding for body weight. Overall, exercise interventions reduced body weight on average in children and adolescents with overweight or obesity, but the wide prediction interval and high heterogeneity suggest that the magnitude of the effect may differ substantially across study contexts, intervention approaches, and participant characteristics (Supplementary Figure S2) .

Fat Mass. The fat mass outcome included 16 studies contributing 23 effect sizes. The three-level random-effects model showed that exercise interventions significantly reduced fat mass in children and adolescents with overweight or obesity compared with control conditions, with a large effect size (Hedges' g = −1.205, 95% CI [−1.905, −0.506], $p = 0.002$) (Supplementary Table S4) . The 95% prediction interval was [−3.850, 1.440], crossing the null and suggesting substantial variability in the true effect that might be observed in future comparable studies. Heterogeneity was statistically significant and substantial, $Q(22) = 232.675$, $p < 0.001$, $I^2 = 94.54\%$, and was mainly attributable to between-study variation (Level 3 $I^2 = 94.54\%$; Level 2 $I^2 \approx 0$) (Supplementary Table S4) . Robust variance estimation clustered at the study level was consistent with the main model, with the pooled effect remaining statistically significant (g = −1.205, 95% CI [−1.924, −0.486], $p = 0.003$), supporting the robustness of the main finding for fat mass. Overall, exercise interventions showed a significant

average reduction in fat mass, and this effect remained stable after RVE correction, although the wide prediction interval and substantial heterogeneity should be considered when interpreting its generalizability (Supplementary Figure S2).

Cardiorespiratory Fitness: VO_2max. The VO_2max outcome included 13 studies contributing 20 effect sizes. The three-level random-effects model showed that exercise interventions significantly improved VO_2max in children and adolescents with overweight or obesity compared with control conditions, with a large effect size (Hedges' g = 1.316, 95% CI [0.667, 1.965], $p < 0.001$) (Supplementary Table S4) . Positive values indicated effects favouring exercise for cardiorespiratory fitness. The 95% prediction interval was [−0.893, 3.526], crossing the null and suggesting that the true effect in future comparable studies may still vary substantially. Heterogeneity was statistically significant and substantial, Q(19) = 182.512, $p < 0.001$, I^2 = 92.63%, and was mainly attributable to between-study variation (Level 3 I^2 = 83.03%; Level 2 I^2 = 9.60%) (Supplementary Table S4) . Robust variance estimation clustered at the study level was consistent with the main model, with the pooled effect remaining statistically significant (g = 1.316, 95% CI [0.640, 1.992], p = 0.001), supporting the robustness of the main finding for VO_2max. Overall, exercise interventions showed a clear average improvement in cardiorespiratory fitness, although the prediction interval and high heterogeneity indicate that the magnitude of this improvement may differ across study contexts (Supplementary Figure S2) .

Supplementary Table S6.

Supplementary Table S6. Meta-regression analyses of intervention duration and participants' mean age for VAT, SAT, BF%, WC, BMI, BW, FM, and VO_2max

Outcome	Predictor	Estimate (β)	95% CI	SE	P value
**VAT**	**Intercept**	-1.213	[-1.893, -0.534]	0.333	<0.001
	Duration (weeks)	0.025	[-0.004, 0.054]	0.014	0.086
	Intercept	-2.061	[-4.064, -0.058]	0.979	0.044
	Mean age (years)	0.105	[-0.051, 0.260]	0.076	0.179
**SAT**	**Intercept**	-0.298	[-0.805, 0.209]	0.241	0.233
	Duration (weeks)	0.002	[-0.016, 0.020]	0.009	0.840
	Intercept	0.133	[-1.113, 1.379]	0.593	0.825
	Mean age (years)	-0.030	[-0.122, 0.063]	0.044	0.511
**BF%**	**Intercept**	-1.197	[-1.765, -0.629]	0.275	<0.001
	Duration (weeks)	0.014	[-0.010, 0.037]	0.011	0.235
	Intercept	-1.663	[-3.465, 0.139]	0.871	0.069
	Mean age (years)	0.060	[-0.086, 0.206]	0.070	0.402
**WC**	**Intercept**	-1.094	[-1.656, -0.533]	0.263	<0.001
	Duration (weeks)	0.026	[-0.005, 0.056]	0.014	0.094
	Intercept	-1.125	[-3.012, 0.762]	0.880	0.222
	Mean age (years)	0.037	[-0.121, 0.195]	0.074	0.620
**BMI**	**Intercept**	-1.551	[-2.475, -0.627]	0.447	0.002
	Duration (weeks)	0.028	[-0.011, 0.067]	0.019	0.145
	Intercept	-2.831	[-5.894, 0.232]	1.477	0.068

	Mean age (years)	0.145	[-0.099, 0.390]	0.118	0.231
**BW**	**Intercept**	-1.345	[-2.433, -0.257]	0.518	0.018
	Duration (weeks)	0.029	[-0.016, 0.073]	0.021	0.197
	Intercept	-3.141	[-6.742, 0.459]	1.707	0.083
	Mean age (years)	0.184	[-0.096, 0.465]	0.133	0.183
**FM**	**Intercept**	-1.949	[-3.107, -0.790]	0.557	0.002
	Duration (weeks)	0.037	[-0.010, 0.083]	0.022	0.116
	Intercept	-3.754	[-7.488, -0.019]	1.790	0.049
	Mean age (years)	0.200	[-0.090, 0.490]	0.139	0.166
**VO_2max**	**Intercept**	2.255	[0.955, 3.555]	0.619	0.002
	Duration (weeks)	-0.064	[-0.143, 0.014]	0.037	0.102
	Intercept	0.460	[-3.499, 4.420]	1.885	0.810
	Mean age (years)	0.073	[-0.261, 0.408]	0.159	0.650

Note. VAT, visceral adipose tissue; SAT, subcutaneous adipose tissue; BF%, body fat percentage; WC, waist circumference; BMI, body mass index; BW, body weight; FM, fat mass; VO_2max, maximal oxygen uptake; β, meta-regression coefficient; CI, confidence interval; SE, standard error. Intervention duration and participants' mean age were examined in separate three-level meta-regression models. A positive β indicates that the effect size increased as the predictor increased, whereas a negative β indicates that the effect size decreased as the predictor increased. Negative values favour exercise for adiposity-related outcomes, whereas positive values favour exercise for VO_2max.

Supplementary Table S7.

Supplementary Table S7. GRADE summary of findings and domain-level certainty assessment for primary and secondary outcomes						
**Outcome**	**Studies / effect sizes**	**Pooled effect (Hedges' g, 95% CI)**	**95% prediction interval**	**Heterogeneity (I^2)**	**Overall certainty**	**Interpretation**
**VAT**	21 / 32	−0.725 [−1.120, −0.330]	−2.399 to 0.948	89.71%	**Moderate**	The pooled comparative estimate indicated lower VAT or a greater reduction in the intervention arms relative to the corresponding comparator arms. Confidence in the average effect is moderate because substantial between-study inconsistency remains.
**SAT**	14 / 20	−0.256 [−0.504, −0.007]	−1.000 to 0.489	68.61%	**Low**	The pooled estimate suggested a small comparative reduction in SAT, but the effect was close to the null and statistical robustness was limited.
**BF%**	18 / 26	−0.926 [−1.262, −0.590]	−2.188 to 0.336	81.60%	**Moderate**	The pooled estimate indicated lower body fat percentage in the intervention arms, although the magnitude of the comparative effect varied substantially across studies.
**WC**	12 / 17	−0.692 [−0.984, −0.400]	−1.547 to 0.163	58.36%	**Moderate**	The pooled estimate indicated lower waist circumference or a greater reduction in the intervention arms, with moderate residual uncertainty across study settings.
**BMI**	17 / 25	−1.010 [−1.580, −0.440]	−3.232 to 1.212	93.12%	**Low**	The pooled estimate indicated lower BMI in the intervention arms, but confidence in the magnitude of the effect is limited by substantial inconsistency and small-study/publication-bias concerns.
**BW**	14 / 20	−0.783 [−1.432, −0.135]	−3.047 to 1.480	92.39%	**Low**	The pooled estimate suggested lower body weight in the intervention arms, but the magnitude was imprecise and sensitive to influential effects.
**FM**	16 / 23	−1.205 [−1.905, −0.506]	−3.850 to 1.440	94.54%	**Moderate**	The pooled estimate indicated lower fat mass in the intervention arms, although substantial heterogeneity limits confidence in the magnitude expected in individual settings.
**VO_2max**	13 / 20	1.316 [0.667, 1.965]	−0.893 to 3.526	92.63%	**Moderate**	The pooled estimate indicated greater improvement in cardiorespiratory fitness in the intervention arms, but the magnitude of improvement varied considerably across studies.

Note. GRADE, Grading of Recommendations Assessment, Development and Evaluation; VAT, visceral adipose tissue; SAT, subcutaneous adipose tissue; BF%, body fat percentage; WC, waist circumference; BMI, body mass index; BW, body weight; FM, fat mass; VO_2max, maximal oxygen uptake; CI, confidence interval; I^2, inconsistency statistic. Negative Hedges' g values indicate lower adiposity-related values or greater reductions in the intervention arm relative to the comparator arm, whereas positive values for VO_2max indicate greater improvement in the intervention arm relative to the comparator arm.

Panel B. Domain-level GRADE judgments						
**Outcome**	**Risk of bias**	**Inconsistency**	**Indirectness**	**Imprecision**	**Publication bias**	**Overall certainty**
**VAT**	Not serious (0)	Serious (−1)	Not serious (0)	Not serious (0)	Concern considered; no downgrade (0)	**Moderate**
**SAT**	Not serious (0)	Serious (−1)	Not serious (0)	Serious (−1)	Not strongly suspected (0)	**Low**
**BF%**	Not serious (0)	Serious (−1)	Not serious (0)	Not serious (0)	Concern considered; no downgrade (0)	**Moderate**
**WC**	Not serious (0)	Serious (−1)	Not serious (0)	Not serious (0)	Concern considered; no downgrade (0)	**Moderate**
**BMI**	Not serious (0)	Serious (−1)	Not serious (0)	Not serious (0)	Serious concern (−1)	**Low**
**BW**	Not serious (0)	Serious (−1)	Not serious (0)	Serious (−1)	Concern considered; no downgrade (0)	**Low**
**FM**	Not serious (0)	Serious (−1)	Not serious (0)	Not serious (0)	Concern considered; no downgrade (0)	**Moderate**
**VO_2max**	Not serious (0)	Serious (−1)	Not serious (0)	Not serious (0)	Concern considered; no downgrade (0)	**Moderate**

Domain judgments are expressed as no downgrade (0) or one-level downgrade (−1). For publication bias, signals of small-study effects were considered alongside funnel-plot asymmetry, Egger-type tests, trim-and-fill analyses, fail-safe N, heterogeneity, and sensitivity analyses. Closely related statistical signals were evaluated jointly to avoid double downgrading for the same underlying source of uncertainty.

Panel C. Detailed rationale for domain judgments and downgrading decisions		
**Outcome / domain**	**GRADE decision**	**Rationale**
**Risk of bias – all outcomes**	No downgrade	Although several contributing studies were judged to have high overall risk of bias, outcome-level subgroup analyses generally did not show a consistent systematic shift in pooled comparative effects according to risk-of-bias category. VAT, WC, BF%, BMI, BW, FM, and VO_2max showed no statistically significant between-category differences. SAT showed a nominal subgroup difference, but this arose from an exploratory analysis with small numbers of studies in several strata and was not consistent with the pattern across the other outcomes.
**Indirectness – all outcomes**	No downgrade	The populations, exercise-based intervention/comparator contrasts, and outcomes were directly relevant to the prespecified review question concerning children and adolescents with overweight or obesity. Variation in exercise modality and comparator type was part of the intended comparative framework rather than evidence of indirectness.
**VAT – inconsistency**	Downgrade 1 level	Substantial heterogeneity (I^2 = 89.71%) and a 95% prediction interval crossing the null (−2.399 to 0.948) indicated important between-study variability in the magnitude and potentially the direction of effects.

**VAT – publication bias**	No additional downgrade	Contour-enhanced funnel asymmetry and Egger-type analyses indicated possible small-study effects. However, trim-and-fill imputed no missing effect sizes, the adjusted pooled estimate remained statistically significant and directionally consistent, and the fail-safe N was 120. These mixed diagnostics were considered insufficient for an additional downgrade.
**SAT – inconsistency**	Downgrade 1 level	Heterogeneity was moderate-to-high ($I^2 = 68.61\%$) and the prediction interval crossed the null (−1.000 to 0.489).
**SAT – imprecision**	Downgrade 1 level	The pooled 95% CI (−0.504 to −0.007) approached the null, the average effect was small, and study-level robust variance estimation produced a borderline interval extending approximately to the null, indicating limited statistical precision.
**SAT – publication bias**	No downgrade	No clear small-study effect was identified in the conventional or three-level Egger-type analyses, and trim-and-fill did not impute missing effects. Although the fail-safe N was low, this alone was not treated as sufficient evidence of publication bias.
**BF% – inconsistency**	Downgrade 1 level	Heterogeneity was high ($I^2 = 81.60\%$) and the prediction interval crossed the null (−2.188 to 0.336).
**BF% – publication bias**	No additional downgrade	Trim-and-fill imputed five potentially missing effect sizes, but the adjusted effect remained statistically significant and in the same direction ($g \approx -0.750$). Given the substantial heterogeneity and the exploratory nature of trim-and-fill under such conditions, no additional downgrade was applied.
**WC – inconsistency**	Downgrade 1 level	Heterogeneity was moderate ($I^2 = 58.36\%$) and the prediction interval crossed the null (−1.547 to 0.163).
**WC – publication bias**	No additional downgrade	Egger-based analyses suggested possible small-study effects, but trim-and-fill imputed no missing effects and the adjusted estimate remained essentially unchanged.
**BMI – inconsistency**	Downgrade 1 level	Heterogeneity was very high ($I^2 = 93.12\%$) and the prediction interval was wide and crossed the null (−3.232 to 1.212).
**BMI – publication bias**	Downgrade 1 level	Publication-bias/small-study-effect diagnostics raised concern and trim-and-fill imputed three potentially missing effect sizes. Although the adjusted estimate remained statistically significant, uncertainty remained regarding the magnitude of the pooled effect.
**BW – inconsistency**	Downgrade 1 level	Heterogeneity was very high ($I^2 = 92.39\%$) and the prediction interval was wide and crossed the null (−3.047 to 1.480).
**BW – imprecision**	Downgrade 1 level	The pooled CI was relatively wide and close to the null, and removal of identified outlying effects substantially attenuated the pooled estimate from $g = -0.783$ to approximately −0.316, indicating uncertainty in the magnitude of the effect.
**BW – publication bias**	No additional downgrade	Trim-and-fill did not impute missing effects. Although the fail-safe N was relatively modest and some small-study-effect uncertainty remained, the available diagnostics were not considered sufficient for a separate publication-bias downgrade.
**FM – inconsistency**	Downgrade 1 level	Heterogeneity was very high ($I^2 = 94.54\%$) and the prediction interval was wide and crossed the null (−3.850 to 1.440).
**FM – publication bias**	No additional downgrade	Some statistical signals of small-study effects were present, but trim-and-fill did not impute missing effects and sensitivity analyses retained a statistically significant effect in the same direction.

**VO_2max – inconsistency**	Downgrade 1 level	Heterogeneity was very high ($I^2 = 92.63\%$) and the prediction interval crossed the null (−0.893 to 3.526).
**VO_2max – publication bias**	No additional downgrade	Trim-and-fill imputed five potentially missing effects and reduced the pooled estimate; however, the adjusted effect remained statistically significant and clearly positive ($g \approx 1.015$). In view of the high heterogeneity and the limitations of trim-and-fill as a diagnostic under substantial heterogeneity, this was treated as a concern but not as sufficient evidence for an additional downgrade.
**Imprecision rule for outcomes not downgraded**	No downgrade	When the pooled 95% CI clearly excluded the null and the principal uncertainty was attributable primarily to between-study heterogeneity, prediction intervals crossing the null were considered principally within the inconsistency domain to avoid double downgrading for the same statistical feature.
Certainty was initially considered high because the evidence base for each outcome was derived predominantly from randomized controlled trials. Methodological limitations arising from randomized and partially randomized trials were evaluated within the risk-of-bias domain. Final certainty was classified as high, moderate, low, or very low according to the cumulative downgrading decisions across the five GRADE domains.		

Supplementary Table S8.

Supplementary Table S8. Trim-and-fill adjusted estimates and fail-safe N for VAT, SAT, BF%, WC, BMI, BW, FM, and VO₂max										
**Outcome**	**Original k**	**Missing studies imputed**	**Imputation side**	**Adjusted k**	**Trim-and-fill estimate (Hedges' g)**	**95% CI**	**P value**	**I² (%)**	**Fail-safe N**	**Interpretation**
**VAT**	32	0	Right	32	-0.7279	[-1.0426, -0.4132]	<0.001	89.73	**120**	No studies were imputed; the significant VAT-reducing effect remained stable.
**SAT**	20	0	Left	20	-0.2515	[-0.4471, -0.0559]	0.0117	67.39	**11**	No studies were imputed; however, the fail-safe N was small, suggesting limited robustness.
**BF%**	26	5	Right	31	-0.7504	[-1.0939, -0.4069]	<0.001	88.96	**230**	Five studies were imputed; the adjusted effect remained significant, although smaller than the unadjusted estimate.
**WC**	17	0	Right	17	-0.6945	[-0.9633, -0.4257]	<0.001	58.40	**72**	No studies were imputed; the significant effect remained stable.
**BMI**	25	3	Right	28	-0.9297	[-1.5186, -0.3408]	0.0020	95.97	**101**	Three studies were imputed; the adjusted effect remained significant despite very high heterogeneity.
**BW**	20	0	Right	20	-0.8619	[-1.4251, -0.2987]	0.0027	93.60	**24**	No studies were imputed; the effect remained significant, but the fail-safe N suggested moderate robustness.
**FM**	23	0	Right	23	-1.3276	[-1.9286, -0.7267]	<0.001	95.25	**75**	No studies were imputed; the significant effect remained stable despite very high heterogeneity.

**VO_2max**	20	5	Left	25	1.0146	[0.4673, 1.5618]	0.0003	94.13	**111**	Five studies were imputed; the adjusted effect remained significant, supporting a robust positive effect on cardiorespiratory fitness.

Note. VAT, visceral adipose tissue; SAT, subcutaneous adipose tissue; BF%, body fat percentage; WC, waist circumference; BMI, body mass index; BW, body weight; FM, fat mass; VO_2max, maximal oxygen uptake; CI, confidence interval; k, number of effect sizes; I^2, inconsistency statistic. Trim-and-fill analyses were conducted as publication-bias sensitivity diagnostics using random-effects models; the imputation side indicates the side of the funnel plot on which potentially missing studies were estimated. Negative values favour exercise for adiposity-related outcomes, whereas positive values favour exercise for VO_2max. Fail-safe N indicates the number of additional null-effect studies required to increase the observed significance level to the target level of 0.05.

Supplementary Table S9.

Supplementary Table S9. Original and outlier-removed pooled effects for VAT, SAT, BF%, WC, BMI, BW, FM, and VO_2max

Outcome	Model	k	Outliers removed, n	Hedges' g	95% CI	P value	Change relative to original model	Sensitivity interpretation
VAT	**Original model**	32		-0.725	[-1.120, -0.330]	<0.001	Reference model	Directionally robust; magnitude partly influenced by large-effect studies.
	**After removing outliers**	25	7	-0.384	[-0.558, -0.209]	<0.001	Attenuated but remained significant	Directionally robust; magnitude partly influenced by large-effect studies.
SAT	**Original model**	20		-0.256	[-0.504, -0.007]	0.044	Reference model	Direction stable; statistical robustness weaker than VAT.
	**After removing outliers**	17	3	-0.318	[-0.501, -0.134]	0.002	Strengthened after outlier removal; heterogeneity reduced	Direction stable; statistical robustness weaker than VAT.
BF%	**Original model**	26		-0.926	[-1.262, -0.590]	<0.001	Reference model	Robust.
	**After removing outliers**	23	3	-0.880	[-1.168, -0.592]	<0.001	Little change	Robust.
WC	**Original model**	17		-0.692	[-0.984, -0.400]	<0.001	Reference model	Robust.
	**After removing outliers**	15	2	-0.657	[-0.926, -0.388]	<0.001	Little change; heterogeneity reduced	Robust.
BMI	**Original model**	25		-1.010	[-1.580, -0.440]	0.001	Reference model	Directionally robust; magnitude influenced by several outlying effects.
	**After removing outliers**	18	7	-0.885	[-1.367, -0.404]	0.001	Attenuated but remained significant	Directionally robust; magnitude influenced by several outlying effects.

**BW**	**Original model**	20		-0.783	[-1.432, -0.135]	0.021	Reference model	Effect direction stable, but magnitude sensitive to outliers.
	**After removing outliers**	13	7	-0.316	[-0.542, -0.091]	0.010	Substantially attenuated; heterogeneity reduced	Effect direction stable, but magnitude sensitive to outliers.
**FM**	**Original model**	23		-1.205	[-1.905, -0.506]	0.002	Reference model	Robust, with residual heterogeneity.
	**After removing outliers**	17	6	-1.090	[-1.619, -0.561]	<0.001	Little change; heterogeneity persisted	Robust, with residual heterogeneity.
**VO_2max**	**Original model**	20		1.316	[0.667, 1.965]	<0.001	Reference model	Robust positive effect on cardiorespiratory fitness.
	**After removing outliers**	13	7	1.730	[1.211, 2.249]	<0.001	Strengthened after outlier removal; heterogeneity persisted	Robust positive effect on cardiorespiratory fitness.

Note. VAT, visceral adipose tissue; SAT, subcutaneous adipose tissue; BF%, body fat percentage; WC, waist circumference; BMI, body mass index; BW, body weight; FM, fat mass; VO_2max, maximal oxygen uptake; CI, confidence interval; k, number of effect sizes; g, Hedges' g. Each outcome is reported using two rows: Original model and After removing outliers. Negative values favour exercise for adiposity-related outcomes, whereas positive values favour exercise for VO_2max. Outlier removal was performed as a sensitivity analysis after identifying potentially influential or extreme effect sizes; the three-level meta-analysis model was then refitted using the reduced dataset.

Supplementary Table S10.

Supplementary Table S10. Compact leave-one-effect-out sensitivity analyses for VAT, SAT, BF%, WC, BMI, BW, FM, and VO_2max

**VAT**		**SAT**		**BF%**		**WC**		**BMI**		**BW**		**FM**		**VO_2max**	
**Effect ID**	**Hedges' g [95% CI]**	**Effect ID**	**Hedges' g [95% CI]**	**Effect ID**	**Hedges' g [95% CI]**	**Effect ID**	**Hedges' g [95% CI]**	**Effect ID**	**Hedges' g [95% CI]**	**Effect ID**	**Hedges' g [95% CI]**	**Effect ID**	**Hedges' g [95% CI]**	**Effect ID**	**Hedges' g [95% CI]**
**1**	-0.74 [-1.15 to -0.32]	**1**	-0.24 [-0.50 to 0.03]	**1**	-0.94 [-1.30 to -0.58]	**1**	-0.75 [-1.04 to -0.46]	**1**	-1.07 [-1.67 to -0.47]	**1**	-0.81 [-1.52 to -0.11]	**1**	-1.24 [-1.99 to -0.49]	**1**	1.46 [0.83 to 2.09]
**2**	-0.75 [-1.17 to -0.34]	**2**	-0.27 [-0.54 to 0.00]	**2**	-0.95 [-1.31 to -0.59]	**2**	-0.74 [-1.04 to -0.44]	**2**	-1.03 [-1.60 to -0.46]	**2**	-0.86 [-1.54 to -0.18]	**2**	-1.28 [-2.02 to -0.55]	**2**	1.41 [0.74 to 2.09]
**3**	-0.72 [-1.12 to -0.33]	**3**	-0.27 [-0.53 to -0.01]	**3**	-0.93 [-1.27 to -0.60]	**3**	-0.65 [-0.96 to -0.34]	**3**	-1.00 [-1.58 to -0.42]	**3**	-0.78 [-1.48 to -0.07]	**3**	-1.20 [-1.95 to -0.45]	**3**	1.34 [0.69 to 1.99]
**4**	-0.73 [-1.12 to -0.33]	**4**	-0.24 [-0.47 to 0.00]	**4**	-0.92 [-1.26 to -0.58]	**4**	-0.73 [-1.03 to -0.42]	**4**	-1.09 [-1.67 to -0.51]	**4**	-0.82 [-1.52 to -0.12]	**4**	-1.24 [-1.99 to -0.50]	**4**	1.34 [0.68 to 1.99]
**5**	-0.75 [-1.16 to -0.34]	**5**	-0.29 [-0.54 to -0.04]	**5**	-0.98 [-1.31 to -0.65]	**5**	-0.69 [-1.01 to -0.38]	**5**	-1.04 [-1.65 to -0.44]	**5**	-0.81 [-1.52 to -0.11]	**5**	-1.23 [-1.99 to -0.48]	**5**	1.27 [0.57 to 1.96]

**6**	-0.71 [-1.12 to -0.30]	**6**	-0.21 [-0.45 to 0.03]	**6**	-0.92 [-1.27 to -0.56]	**6**	-0.71 [-1.02 to -0.39]	**6**	-1.06 [-1.66 to -0.46]	**6**	-0.78 [-1.44 to -0.13]	**6**	-1.20 [-1.91 to -0.50]	**6**	1.38 [0.69 to 2.07]
**7**	-0.74 [-1.16 to -0.33]	**7**	-0.26 [-0.52 to 0.00]	**7**	-0.94 [-1.30 to -0.59]	**7**	-0.74 [-1.04 to -0.45]	**7**	-1.04 [-1.65 to -0.44]	**7**	-0.79 [-1.44 to -0.14]	**7**	-1.21 [-1.91 to -0.51]	**7**	1.30 [0.63 to 1.97]
**8**	-0.76 [-1.17 to -0.34]	**8**	-0.23 [-0.50 to 0.03]	**8**	-0.94 [-1.30 to -0.57]	**8**	-0.67 [-0.98 to -0.36]	**8**	-1.01 [-1.59 to -0.43]	**8**	-0.85 [-1.54 to -0.15]	**8**	-1.27 [-2.01 to -0.53]	**8**	1.35 [0.70 to 1.99]
**9**	-0.73 [-1.12 to -0.33]	**9**	-0.26 [-0.51 to -0.01]	**9**	-0.92 [-1.26 to -0.58]	**9**	-0.66 [-0.97 to -0.35]	**9**	-1.03 [-1.61 to -0.45]	**9**	-0.79 [-1.44 to -0.14]	**9**	-1.27 [-2.01 to -0.53]	**9**	1.27 [0.62 to 1.92]
**10**	-0.72 [-1.12 to -0.33]	**10**	-0.25 [-0.50 to -0.00]	**10**	-0.93 [-1.27 to -0.59]	**10**	-0.71 [-1.02 to -0.40]	**10**	-1.07 [-1.67 to -0.47]	**10**	-0.78 [-1.44 to -0.13]	**10**	-1.21 [-1.91 to -0.51]	**10**	1.35 [0.67 to 2.03]
**11**	-0.72 [-1.12 to -0.33]	**11**	-0.26 [-0.51 to -0.00]	**11**	-0.97 [-1.32 to -0.62]	**11**	-0.65 [-0.97 to -0.34]	**11**	-1.07 [-1.66 to -0.47]	**11**	-0.82 [-1.51 to -0.12]	**11**	-1.21 [-1.91 to -0.51]	**11**	1.26 [0.57 to 1.96]
**12**	-0.74 [-1.14 to -0.35]	**12**	-0.26 [-0.51 to -0.00]	**12**	-0.97 [-1.31 to -0.62]	**12**	-0.71 [-1.03 to -0.39]	**12**	-1.03 [-1.61 to -0.45]	**12**	-0.67 [-1.33 to -0.02]	**12**	-1.23 [-1.98 to -0.49]	**12**	1.25 [0.60 to 1.89]
**13**	-0.71 [-1.11 to -0.31]	**13**	-0.27 [-0.52 to -0.03]	**13**	-0.93 [-1.27 to -0.60]	**13**	-0.69 [-1.00 to -0.38]	**13**	-1.03 [-1.61 to -0.45]	**13**	-0.78 [-1.43 to -0.12]	**13**	-1.19 [-1.89 to -0.49]	**13**	1.36 [0.68 to 2.04]
**14**	-0.75 [-1.17 to -0.33]	**14**	-0.26 [-0.53 to 0.01]	**14**	-0.93 [-1.27 to -0.60]	**14**	-0.62 [-0.90 to -0.35]	**14**	-1.05 [-1.65 to -0.44]	**14**	-0.79 [-1.44 to -0.15]	**14**	-1.21 [-1.92 to -0.51]	**14**	1.29 [0.64 to 1.93]
**15**	-0.75 [-1.17 to -0.34]	**15**	-0.25 [-0.51 to 0.02]	**15**	-0.93 [-1.29 to -0.58]	**15**	-0.68 [-1.02 to -0.34]	**15**	-0.99 [-1.56 to -0.43]	**15**	-0.77 [-1.43 to -0.12]	**15**	-1.11 [-1.83 to -0.40]	**15**	1.33 [0.67 to 2.00]
**16**	-0.73 [-1.12 to -0.34]	**16**	-0.26 [-0.51 to -0.01]	**16**	-0.95 [-1.30 to -0.59]	**16**	-0.58 [-0.85 to -0.30]	**16**	-1.01 [-1.59 to -0.44]	**16**	-0.79 [-1.45 to -0.14]	**16**	-1.19 [-1.89 to -0.49]	**16**	1.29 [0.66 to 1.93]
**17**	-0.73 [-1.12 to -0.33]	**17**	-0.26 [-0.51 to -0.01]	**17**	-0.90 [-1.23 to -0.57]	**17**	-0.71 [-1.02 to -0.39]	**17**	-0.94 [-1.52 to -0.35]	**17**	-0.83 [-1.55 to -0.11]	**17**	-1.22 [-1.92 to -0.51]	**17**	1.31 [0.66 to 1.96]
**18**	-0.74 [-1.16 to -0.33]	**18**	-0.27 [-0.51 to -0.03]	**18**	-0.89 [-1.24 to -0.54]			**18**	-1.03 [-1.61 to -0.44]	**18**	-0.73 [-1.32 to -0.13]	**18**	-1.21 [-1.91 to -0.50]	**18**	1.27 [0.65 to 1.89]
**19**	-0.71 [-1.10 to -0.31]	**19**	-0.27 [-0.51 to -0.03]	**19**	-0.95 [-1.30 to -0.60]			**19**	-0.98 [-1.55 to -0.42]	**19**	-0.75 [-1.37 to -0.13]	**19**	-1.21 [-1.91 to -0.51]	**19**	1.33 [0.66 to 2.00]
**20**	-0.74 [-1.14 to -0.34]	**20**	-0.23 [-0.49 to 0.02]	**20**	-0.90 [-1.23 to -0.57]			**20**	-0.97 [-1.53 to -0.41]	**20**	-0.77 [-1.42 to -0.13]	**20**	-1.18 [-1.85 to -0.50]	**20**	1.38 [0.69 to 2.07]
**21**	-0.65 [-1.02 to -0.27]			**21**	-0.93 [-1.27 to -0.59]			**21**	-1.03 [-1.62 to -0.45]			**21**	-1.17 [-1.84 to -0.51]		

**22**	-0.71 [-1.10 to -0.31]			**22**	-0.92 [-1.25 to -0.58]			**22**	-1.08 [-1.76 to -0.41]			**22**	-1.16 [-1.84 to -0.49]		
**23**	-0.74 [-1.15 to -0.34]			**23**	-0.93 [-1.27 to -0.58]			**23**	-0.94 [-1.45 to -0.43]			**23**	-1.23 [-1.96 to -0.50]		
**24**	-0.73 [-1.12 to -0.34]			**24**	-0.89 [-1.20 to -0.58]			**24**	-0.99 [-1.55 to -0.43]						
**25**	-0.72 [-1.12 to -0.33]			**25**	-0.91 [-1.24 to -0.58]			**25**	-1.01 [-1.59 to -0.43]						
**26**	-0.72 [-1.11 to -0.32]			**26**	-0.91 [-1.23 to -0.58]										
**27**	-0.73 [-1.13 to -0.33]														
**28**	-0.69 [-1.06 to -0.33]														
**29**	-0.72 [-1.12 to -0.33]														
**30**	-0.68 [-1.04 to -0.32]														
**31**	-0.72 [-1.12 to -0.33]														
**32**	-0.74 [-1.16 to -0.33]														

Note. VAT, visceral adipose tissue; SAT, subcutaneous adipose tissue; BF%, body fat percentage; WC, waist circumference; BMI, body mass index; BW, body weight; FM, fat mass; VO_2max, maximal oxygen uptake; CI, confidence interval. Each cell reports the re-estimated pooled Hedges' g and 95% CI after omitting the corresponding effect size. Negative values favour exercise for adiposity-related outcomes, whereas positive values favour exercise for VO_2max.

Supplementary Table S11. Shared-control-adjusted sensitivity analyses for primary and secondary outcomes

Panel A. Comparison of original and shared-control-adjusted pooled effects

Outcome	Role	k	Shared-control groups	Affected comparisons	Original g [95% CI]	Adjusted g [95% CI]	Original p	Adjusted p	Sensitivity interpretation
**VAT**	Primary	32	10	21	-0.725 [-1.120, -0.330]	-0.716 [-1.105, -0.328]	<0.001	<0.001	No material change
**SAT**	Secondary	20	4	9	-0.256 [-0.504, -0.007]	-0.269 [-0.515, -0.024]	0.044	0.033	No material change
**BF%**	Secondary	26	7	14	-0.926 [-1.262, -0.590]	-0.891 [-1.208, -0.573]	<0.001	<0.001	No material change
**WC**	Primary	17	5	10	-0.692 [-0.984, -0.400]	-0.664 [-0.964, -0.364]	<0.001	<0.001	No material change
**BMI**	Secondary	25	7	14	-1.010 [-1.580, -0.440]	-0.991 [-1.559, -0.424]	0.001	0.001	No material change
**BW**	Secondary	20	5	10	-0.783 [-1.432, -0.135]	-0.777 [-1.419, -0.135]	0.021	0.020	No material change
**FM**	Secondary	23	7	14	-1.205 [-1.905, -0.506]	-1.135 [-1.777, -0.494]	0.002	0.001	No material change
**VO_2max**	Secondary	20	6	12	1.316 [0.667, 1.965]	1.259 [0.629, 1.889]	<0.001	<0.001	No material change

Note. VAT and WC were primary outcomes; SAT, BF%, BMI, BW, FM, and VO_2max were secondary outcomes. For multi-arm trials with two or more eligible comparisons sharing the same control group, the control-group sample size was divided as evenly as possible across the relevant pairwise comparisons, while the corresponding mean and standard deviation were left unchanged. Hedges' g and sampling variances were then recalculated and the three-level model refitted. No outcome changed direction or statistical significance after adjustment.

Supplementary Table S11. Shared-control-adjusted sensitivity analyses for primary and secondary outcomes (continued)

Panel B. Heterogeneity and between-modality tests before and after shared-control adjustment

Outcome	Original I^2 (%)	Adjusted I^2 (%)	Original Level 3 τ^2	Adjusted Level 3 τ^2	Original Level 2 τ^2	Adjusted Level 2 τ^2	Original modality F	Original P subgroup	Adjusted modality F	Adjusted P subgroup
**VAT**	89.706	85.943	0.692	0.649	0.000	0.000	1.924	0.116	2.419	0.056
**SAT**	68.608	61.665	0.130	0.118	0.000	0.000	0.162	0.972	0.152	0.976
**BF%**	81.604	76.885	0.388	0.321	0.000	0.000	0.721	0.638	0.752	0.615
**WC**	58.356	51.130	0.006	0.006	0.165	0.147	1.859	0.184	1.518	0.267
**BMI**	93.116	91.713	1.129	1.100	0.080	0.082	0.581	0.714	0.573	0.720
**BW**	92.389	90.781	1.238	1.196	0.000	0.000	0.478	0.813	0.509	0.791

Outcome	Original I^2 (%)	Adjusted I^2 (%)	Original Level 3 τ^2	Adjusted Level 3 τ^2	Original Level 2 τ^2	Adjusted Level 2 τ^2	Original modality F	Original P subgroup	Adjusted modality F	Adjusted P subgroup
**FM**	94.543	92.448	1.708	1.402	0.000	0.000	1.078	0.416	1.270	0.325
**VO_2max**	92.628	90.956	1.053	1.048	0.122	0.000	2.597	0.088	2.083	0.146

Note. I^2, inconsistency statistic; τ^2, estimated variance component; Level 3, between-study heterogeneity; Level 2, within-study heterogeneity; F, omnibus test statistic for exercise modality; P subgroup, p value for the overall between-modality test. All between-modality tests remained non-significant after shared-control adjustment. For VO_2max, 17 of 20 effect sizes had an exercise-modality classification and contributed to the modality test. The sensitivity procedure followed the approximate split-group approach recommended by the Cochrane Handbook (33).

Supplementary Table S12. Exercise-modality analyses before and after outlier removal
Panel A. Omnibus between-modality tests

Outcome	Outliers removed	Original F(df1, df2)	Original p	After removal F(df1, df2)	After removal p	Conclusion
**VAT**	7	1.924 (6, 25)	0.116	2.058 (5, 19)	0.116	Non-significant before and after removal
**SAT**	3	0.162 (5, 14)	0.972	0.138 (5, 11)	0.980	Non-significant before and after removal
**BF%**	3	0.721 (6, 19)	0.638	0.879 (6, 16)	0.532	Non-significant before and after removal
**WC**	2	1.859 (6, 10)	0.184	0.430 (5, 9)	0.817	Non-significant before and after removal
**BMI**	7	0.581 (5, 19)	0.714	1.971 (5, 12)	0.156	Non-significant before and after removal
**BW**	7	0.478 (6, 13)	0.813	0.408 (5, 7)	0.829	Non-significant before and after removal
**FM**	6	1.078 (6, 16)	0.416	1.325 (5, 11)	0.323	Non-significant before and after removal
**VO_2max**	7	2.597 (6, 10)	0.088	0.702 (5, 6)	0.643	Non-significant before and after removal

Panel B. Modality-specific pooled estimates

Outcome	Modality	Original k	Original g [95% CI]	Original p	After-removal k	After-removal g [95% CI]	After-removal p
**VAT**	**AE[M]**	2	-0.918 [-1.781, -0.054]	0.038	2	-0.323 [-0.935, 0.289]	0.283
**VAT**	**AE[MV]**	8	-0.466 [-0.910, -0.022]	0.040	6	-0.555 [-0.877, -0.232]	0.002
**VAT**	**AE[V]**	4	-0.676 [-1.422, 0.070]	0.074	4	-0.296 [-0.586, -0.006]	0.046
**VAT**	**AE+RT**	6	-0.438 [-0.910, 0.033]	0.067	6	-0.175 [-0.414, 0.063]	0.140
**VAT**	**HIIT**	8	-1.332 [-1.874, -0.789]	<0.001	5	-0.833 [-1.242, -0.423]	<0.001
**VAT**	**HIIT+RT**	1	-1.437 [-3.138, 0.264]	0.094	0	Not estimable	—
**VAT**	**RT**	3	-0.436 [-0.961, 0.088]	0.099	2	-0.222 [-0.637, 0.193]	0.277
**SAT**	**AE[M]**	1	-0.030 [-1.219, 1.158]	0.957	1	-0.030 [-1.051, 0.990]	0.949
**SAT**	**AE[MV]**	4	-0.224 [-0.674, 0.225]	0.302	4	-0.314 [-0.716, 0.088]	0.114
**SAT**	**AE[V]**	5	-0.406 [-0.878, 0.067]	0.087	4	-0.349 [-0.722, 0.024]	0.064
**SAT**	**AE+RT**	6	-0.206 [-0.576, 0.164]	0.253	5	-0.318 [-0.649, 0.013]	0.058
**SAT**	**HIIT**	1	-0.035 [-1.252, 1.181]	0.951	1	-0.035 [-1.089, 1.018]	0.942
**SAT**	**RT**	3	-0.213 [-0.665, 0.238]	0.328	2	-0.375 [-0.856, 0.105]	0.113
**BF%**	**AE[M]**	2	-1.244 [-2.119, -0.368]	0.008	2	-1.168 [-1.973, -0.364]	0.007
**BF%**	**AE[MV]**	6	-1.267 [-1.877, -0.658]	<0.001	4	-1.199 [-1.806, -0.592]	<0.001
**BF%**	**AE[V]**	5	-0.704 [-1.395, -0.014]	0.046	5	-0.682 [-1.213, -0.152]	0.015
**BF%**	**AE+RT**	3	-0.536 [-1.316, 0.244]	0.166	3	-0.558 [-1.178, 0.063]	0.075
**BF%**	**HIIT**	8	-0.980 [-1.529, -0.431]	0.001	7	-0.996 [-1.483, -0.509]	<0.001
**BF%**	**HIIT+RT**	1	-1.641 [-3.182, -0.099]	0.038	1	-1.850 [-3.307, -0.393]	0.016
**BF%**	**RT**	1	-0.600 [-2.223, 1.023]	0.448	1	-0.600 [-1.892, 0.692]	0.340
**WC**	**AE[M]**	1	-0.429 [-1.621, 0.762]	0.441	1	-0.429 [-1.629, 0.770]	0.439
**WC**	**AE[MV]**	4	-0.468 [-1.021, 0.086]	0.089	4	-0.467 [-1.023, 0.090]	0.090

Outcome	Modality	Original k	Original g [95% CI]	Original p	After-removal k	After-removal g [95% CI]	After-removal p
**WC**	**AE[V]**	2	-0.358 [-1.014, 0.297]	0.251	1	-0.772 [-1.872, 0.328]	0.147
**WC**	**AE+RT**	2	-0.594 [-1.405, 0.216]	0.133	2	-0.594 [-1.410, 0.221]	0.134
**WC**	**HIIT**	5	-0.928 [-1.464, -0.391]	0.003	5	-0.928 [-1.468, -0.388]	0.004
**WC**	**HIIT+RT**	1	-2.644 [-4.202, -1.086]	0.004	0	Not estimable	—
**WC**	**RT**	2	-0.697 [-1.515, 0.120]	0.086	2	-0.697 [-1.519, 0.125]	0.087
**BMI**	**AE[M]**	2	-1.297 [-2.807, 0.213]	0.088	2	-1.415 [-2.398, -0.432]	0.009
**BMI**	**AE[MV]**	7	-1.263 [-2.185, -0.341]	0.010	4	-0.387 [-1.191, 0.418]	0.316
**BMI**	**AE[V]**	4	-0.465 [-1.759, 0.829]	0.461	3	-0.611 [-1.610, 0.388]	0.207
**BMI**	**AE+RT**	3	-0.298 [-1.632, 1.036]	0.646	3	-0.332 [-1.208, 0.543]	0.424
**BMI**	**HIIT**	8	-1.456 [-2.327, -0.585]	0.002	5	-1.374 [-2.097, -0.650]	0.001
**BMI**	**HIIT+RT**	1	-1.591 [-3.893, 0.711]	0.164	1	-2.368 [-4.338, -0.398]	0.022
**BW**	**AE[M]**	1	-1.027 [-2.783, 0.728]	0.228	1	-0.256 [-1.083, 0.571]	0.488
**BW**	**AE[MV]**	5	-1.088 [-2.147, -0.029]	0.045	4	-0.305 [-0.787, 0.178]	0.179
**BW**	**AE[V]**	1	-0.181 [-2.073, 1.712]	0.840	1	-0.260 [-1.210, 0.691]	0.539
**BW**	**AE+RT**	4	-0.093 [-1.266, 1.080]	0.867	3	-0.227 [-0.686, 0.233]	0.282
**BW**	**HIIT**	6	-1.197 [-2.190, -0.204]	0.022	3	-0.364 [-0.961, 0.233]	0.192
**BW**	**HIIT+RT**	1	-1.598 [-3.877, 0.681]	0.154	0	Not estimable	—
**BW**	**RT**	2	-0.355 [-1.827, 1.117]	0.611	1	-0.918 [-2.001, 0.165]	0.085
**FM**	**AE[M]**	2	-1.712 [-2.868, -0.556]	0.006	2	-1.215 [-2.225, -0.204]	0.023
**FM**	**AE[MV]**	6	-1.442 [-2.381, -0.504]	0.005	5	-0.901 [-1.646, -0.156]	0.022
**FM**	**AE[V]**	2	-0.367 [-1.697, 0.963]	0.567	1	-0.675 [-1.992, 0.643]	0.284
**FM**	**AE+RT**	4	-0.234 [-1.407, 0.939]	0.678	3	-0.536 [-1.520, 0.447]	0.255
**FM**	**HIIT**	7	-1.932 [-2.847, -1.017]	<0.001	5	-1.439 [-2.214, -0.663]	0.002

Outcome	Modality	Original k	Original g [95% CI]	Original p	After-removal k	After-removal g [95% CI]	After-removal p
**FM**	**HIIT+RT**	1	-1.237 [-3.282, 0.808]	0.218	1	-2.827 [-4.949, -0.704]	0.014
**FM**	**RT**	1	-0.251 [-1.575, 1.074]	0.694	0	Not estimable	—
**VO_2max**	**AE[M]**	2	1.468 [0.440, 2.497]	0.010	1	1.367 [-0.997, 3.732]	0.207
**VO_2max**	**AE[MV]**	2	1.467 [0.330, 2.603]	0.016	2	1.898 [0.238, 3.557]	0.031
**VO_2max**	**AE[V]**	2	0.368 [-1.560, 2.295]	0.680	0	Not estimable	—
**VO_2max**	**AE+RT**	1	0.541 [-1.567, 2.649]	0.580	1	0.541 [-1.764, 2.846]	0.587
**VO_2max**	**HIIT**	7	2.351 [1.522, 3.180]	<0.001	5	2.037 [0.987, 3.088]	0.003
**VO_2max**	**HIIT+RT**	1	1.840 [-0.044, 3.724]	0.055	1	2.555 [-0.000, 5.110]	0.050
**VO_2max**	**RT**	2	1.219 [-0.287, 2.725]	0.101	2	1.219 [-0.427, 2.865]	0.120

Note. Hedges' g values were estimated using three-level random-effects models. The omnibus F test compares exercise modalities. If outlier removal eliminated the only available effect size within a modality, the post-removal estimate was not estimable. Negative g values favour exercise for adiposity/body-composition outcomes; positive g values favour exercise for VO_2max. Abbreviations: AE[M], moderate-intensity aerobic exercise; AE[MV], moderate-to-vigorous aerobic exercise; AE[V], vigorous-intensity aerobic exercise; AE+RT, aerobic plus resistance training; HIIT, high-intensity interval training; RT, resistance training; SAT, subcutaneous adipose tissue; BF%, body fat percentage; WC, waist circumference; BMI, body mass index; BW, body weight; FM, fat mass.

Supplementary Figure S1.

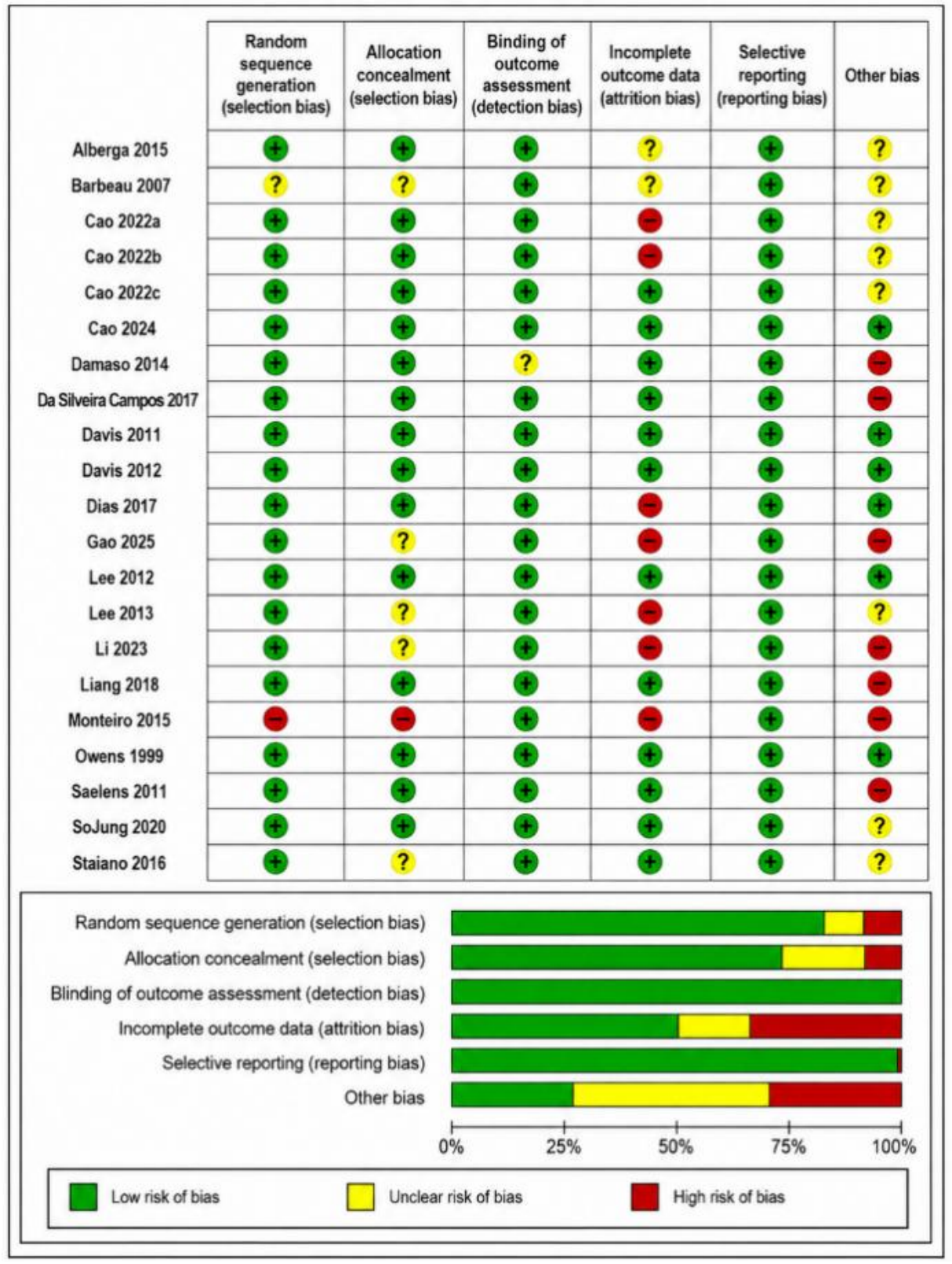

Supplementary Figure S1. Risk-of-bias summary plot for the included randomized controlled trials.

Supplementary Figure S2.

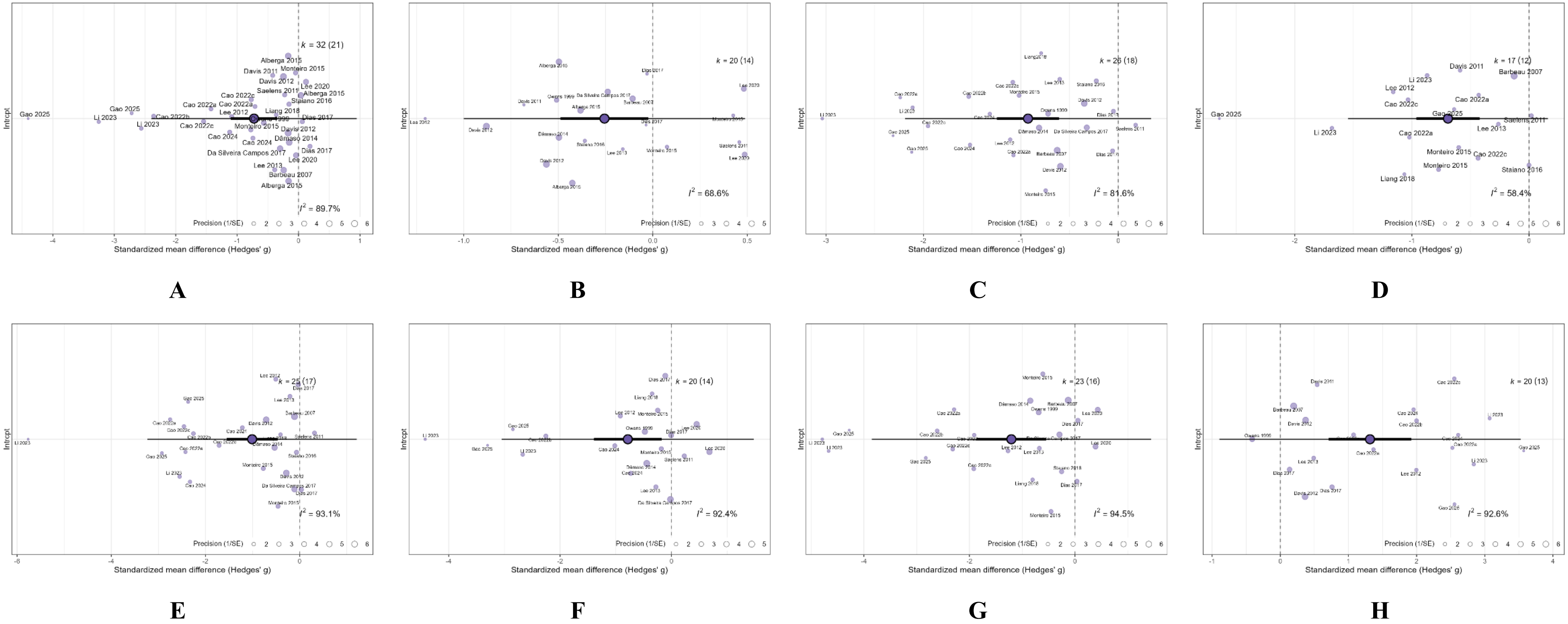

Supplementary Figure S2. Orchard plots of the main pooled effects of exercise interventions for the primary and secondary outcomes: A, visceral adipose tissue; B, subcutaneous adipose tissue; C, body fat percentage; D, waist circumference; E, body mass index; F, body weight; G, fat mass; H, maximal oxygen uptake.

Supplementary Figure S3.

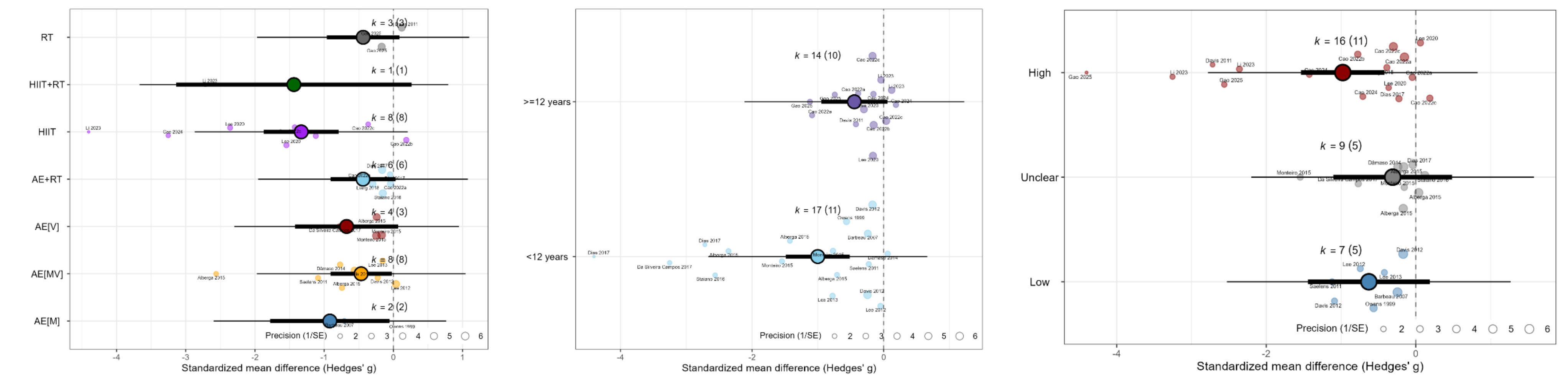

A, visceral adipose tissue

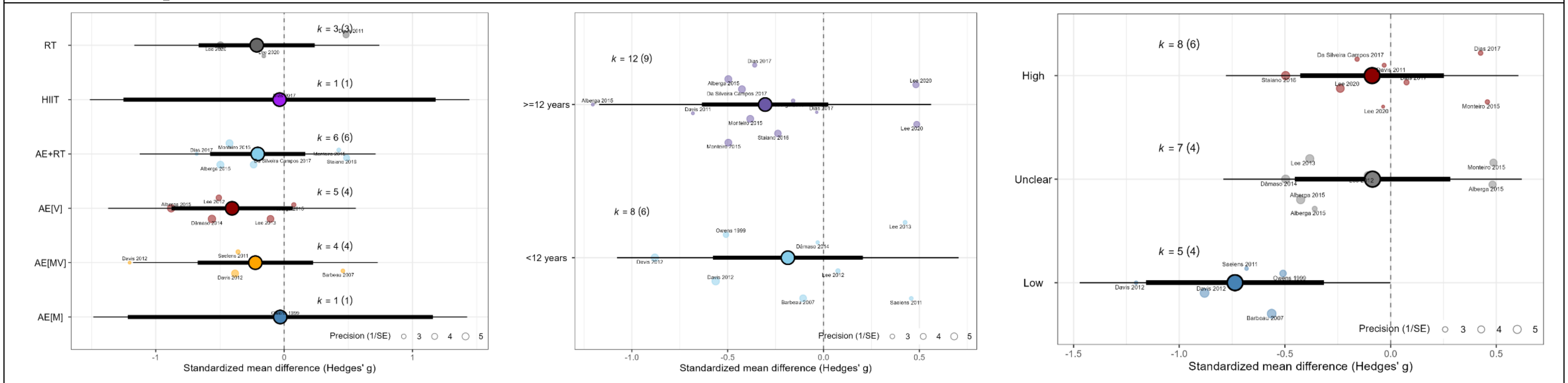

B, subcutaneous adipose tissue

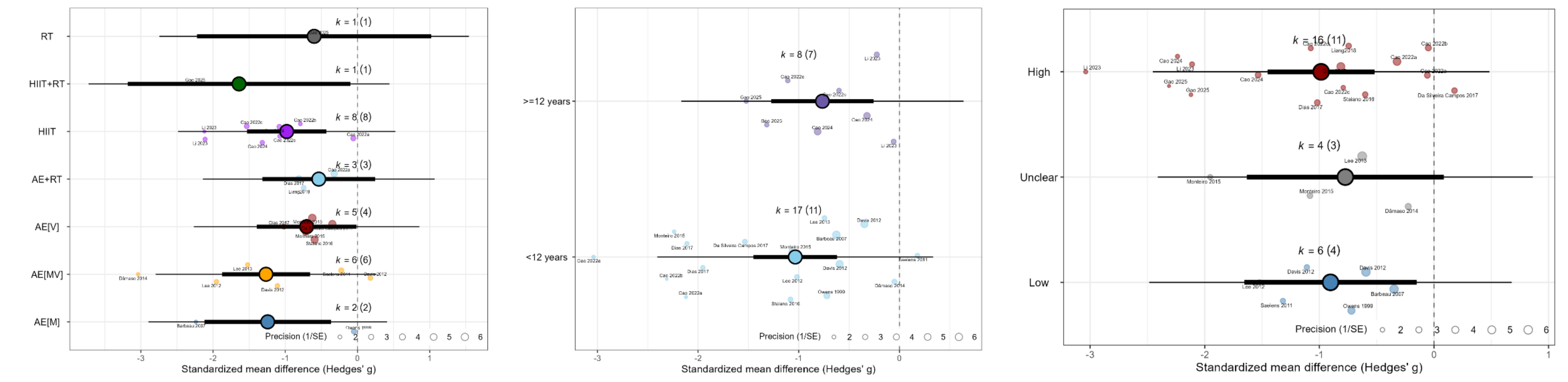

C, body fat percentage

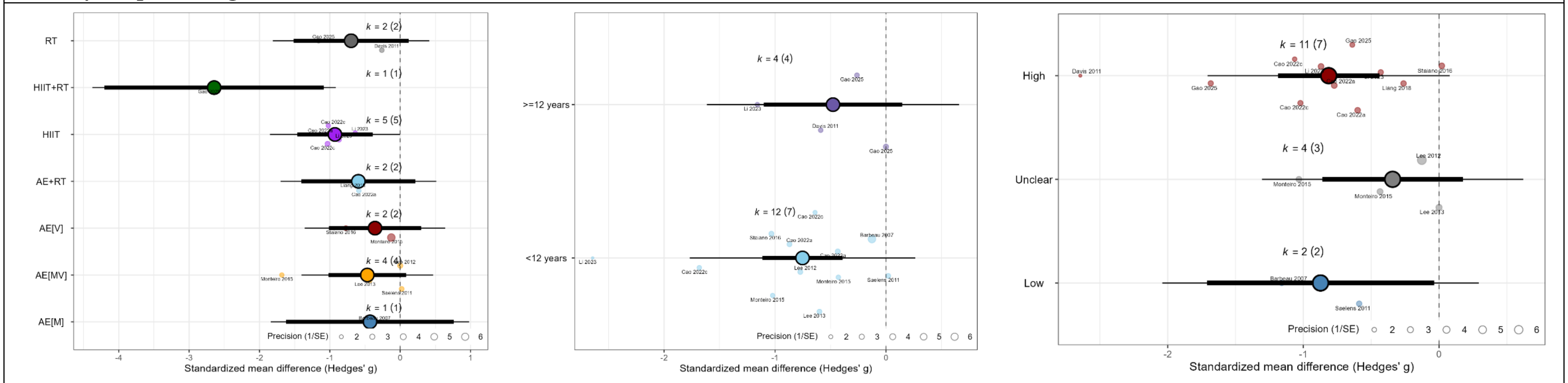

D, waist circumference

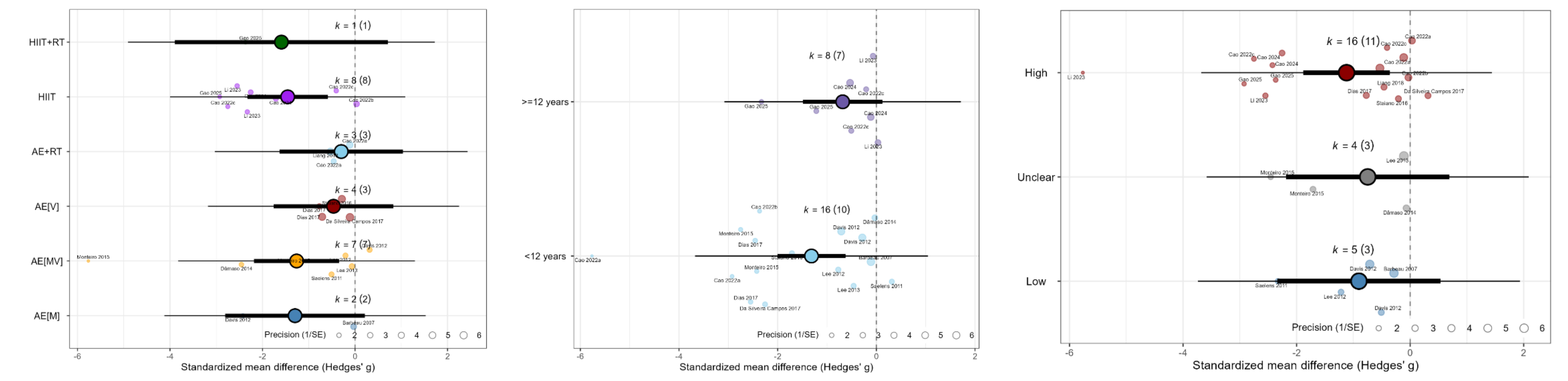

E, body mass index

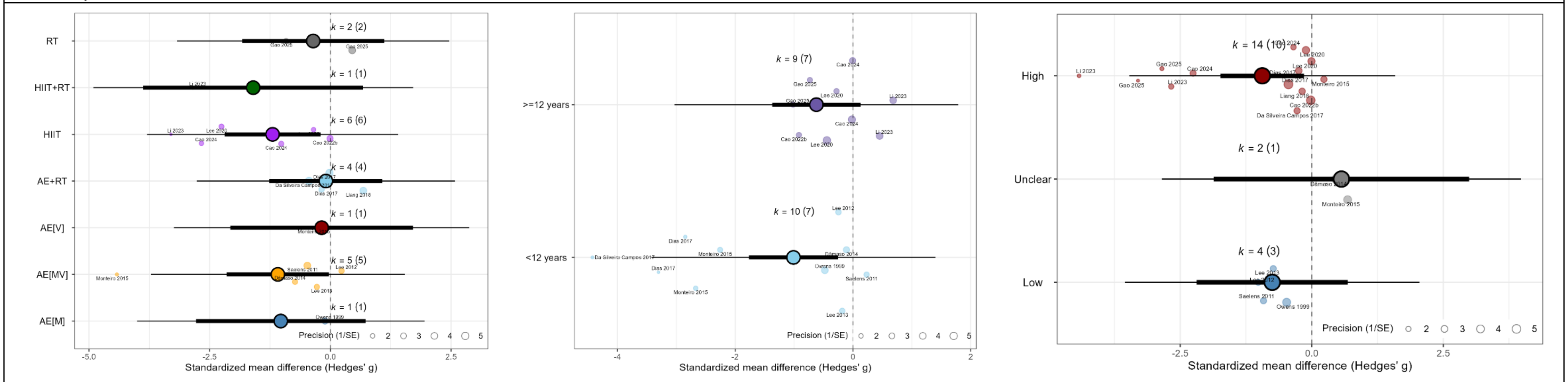

F, body weight

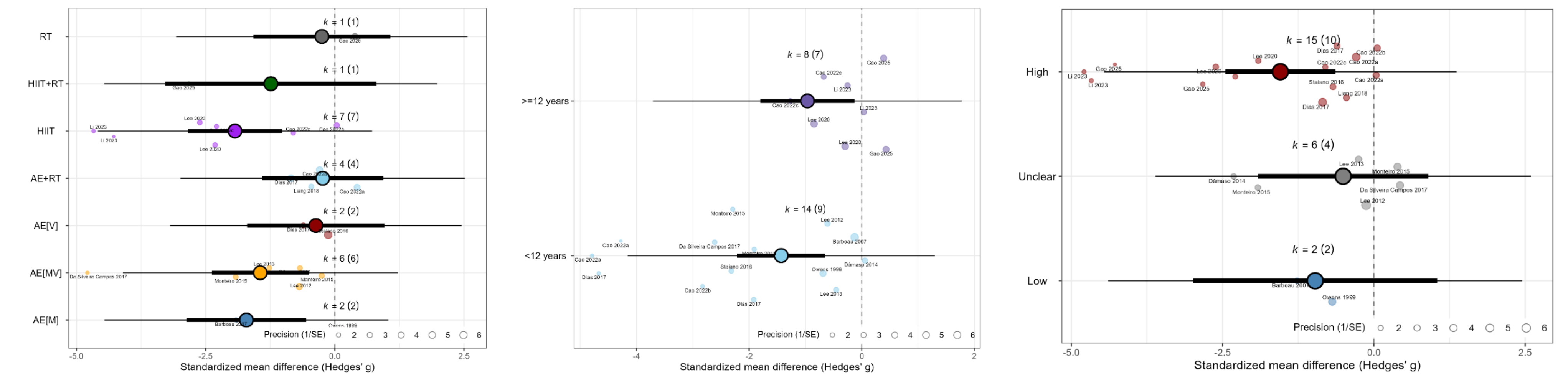

G, fat mass

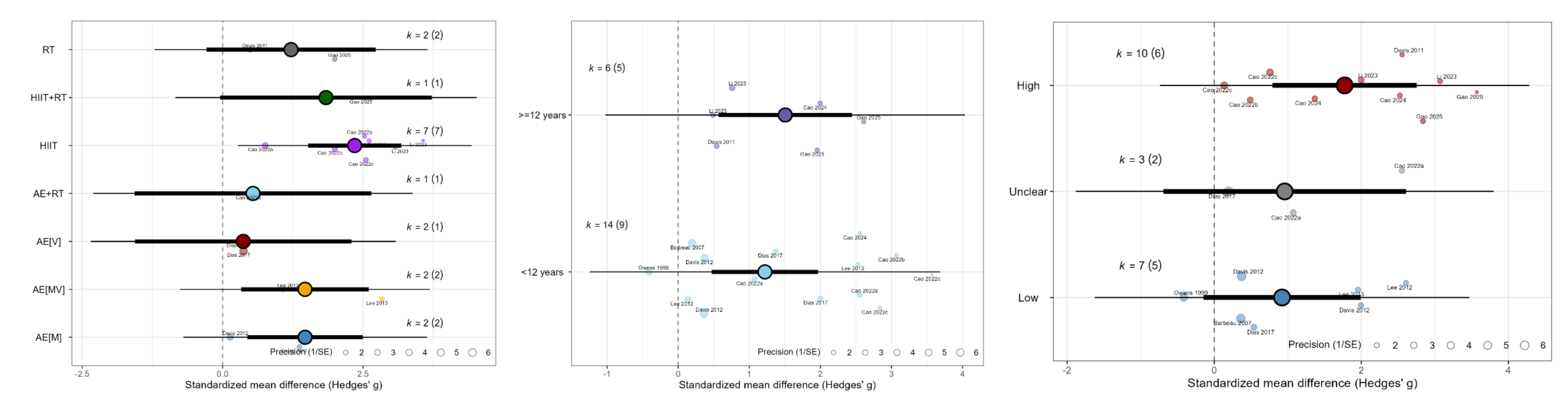

H, maximal oxygen uptake

Supplementary Figure S3. Orchard plots of subgroup analyses by exercise modality, age group, and overall risk of bias for the primary and secondary outcomes: A, visceral adipose tissue; B, subcutaneous adipose tissue; C, body fat percentage; D, waist circumference; E, body mass index; F, body weight; G, fat mass; H, maximal oxygen uptake.

Supplementary Figure S4.

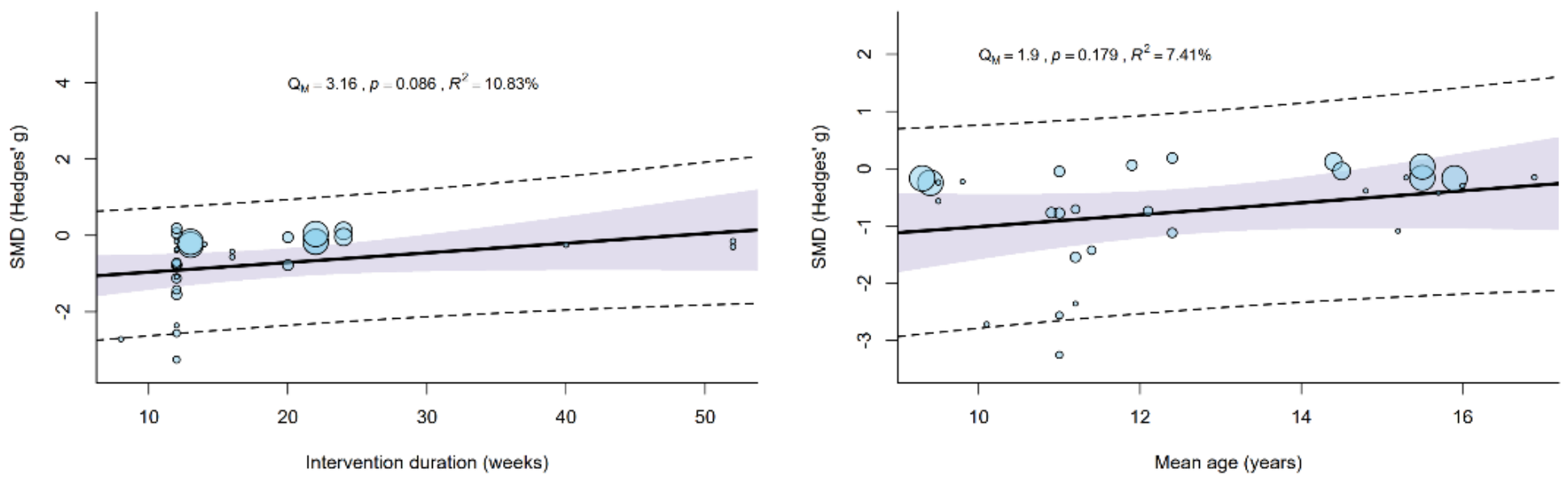

A

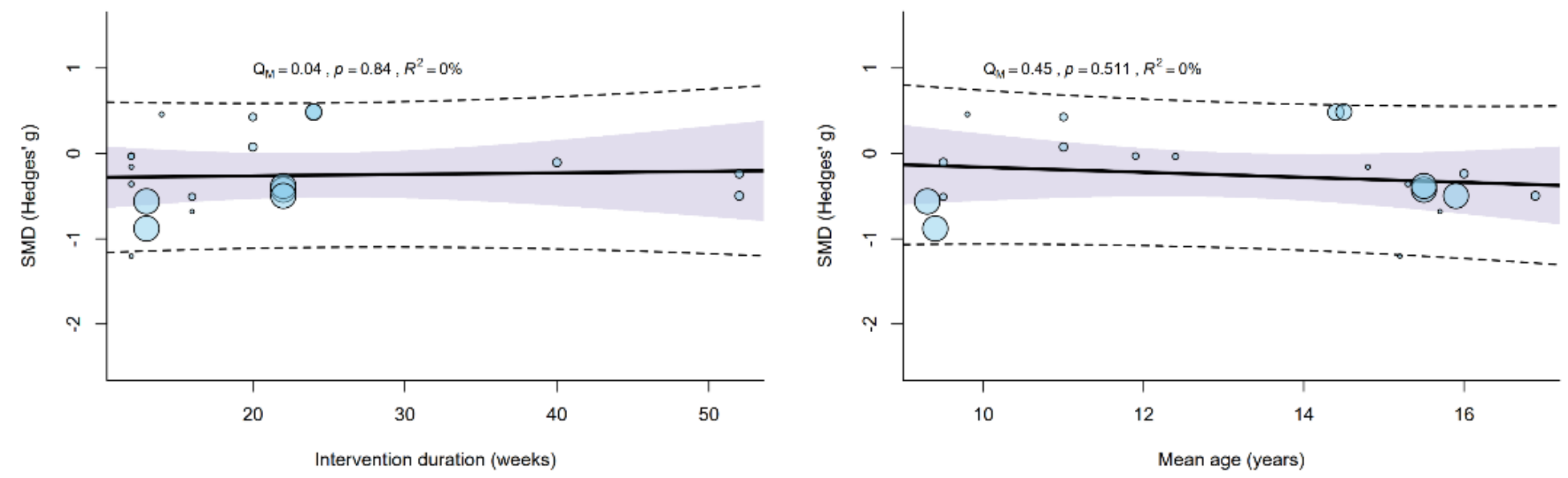

B

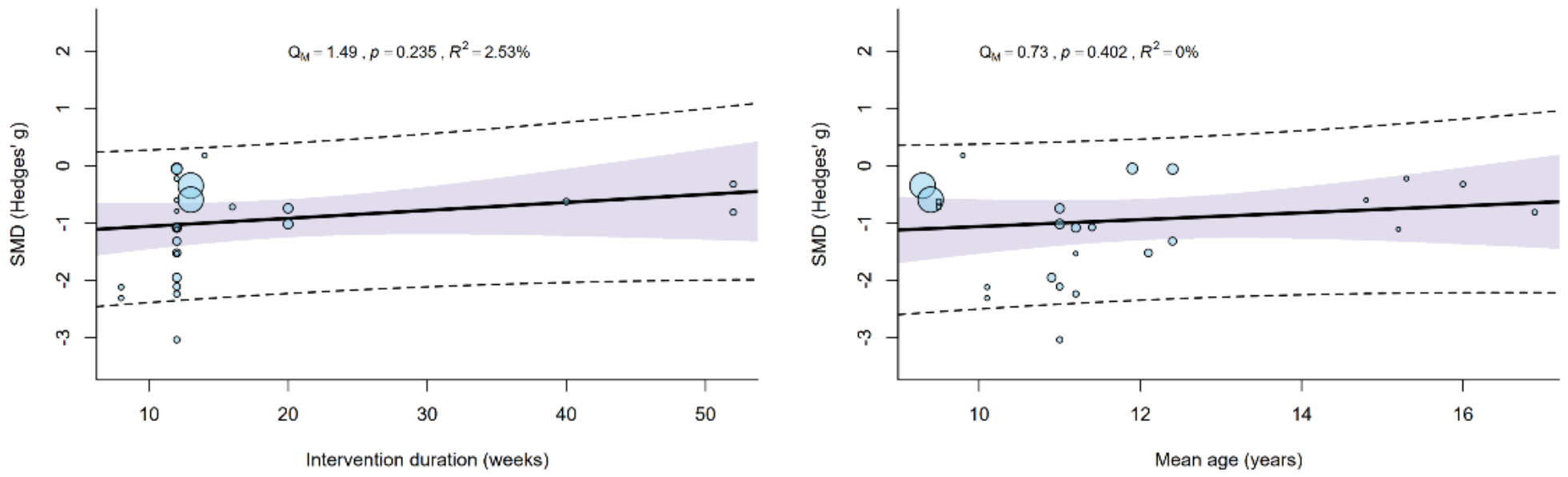

C

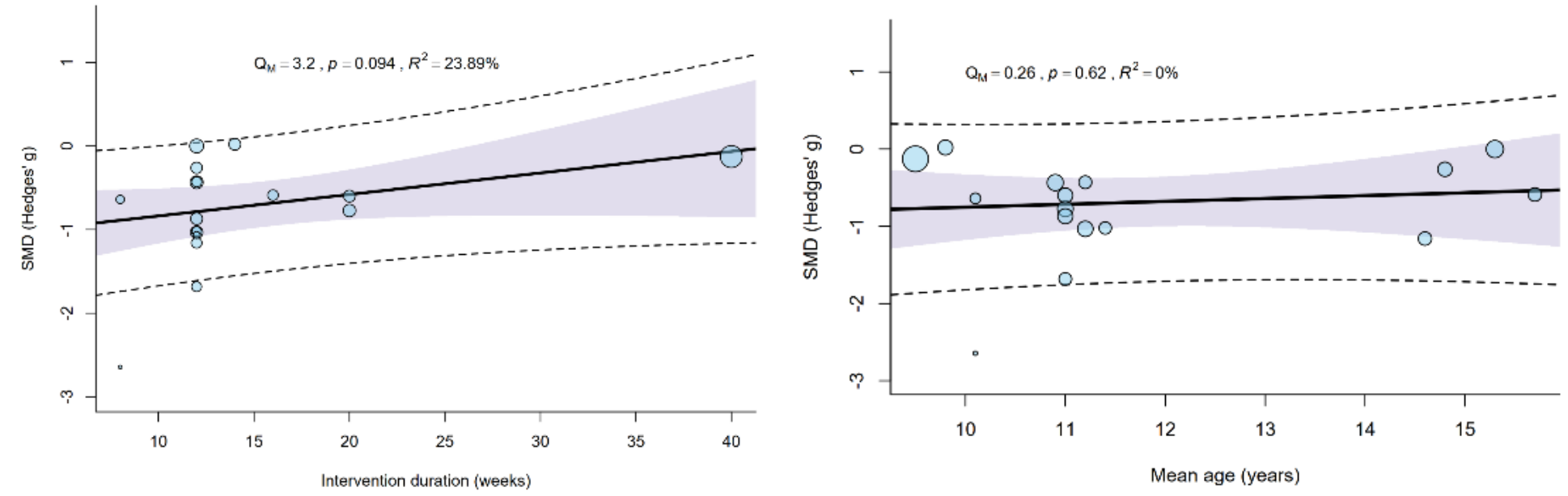

D

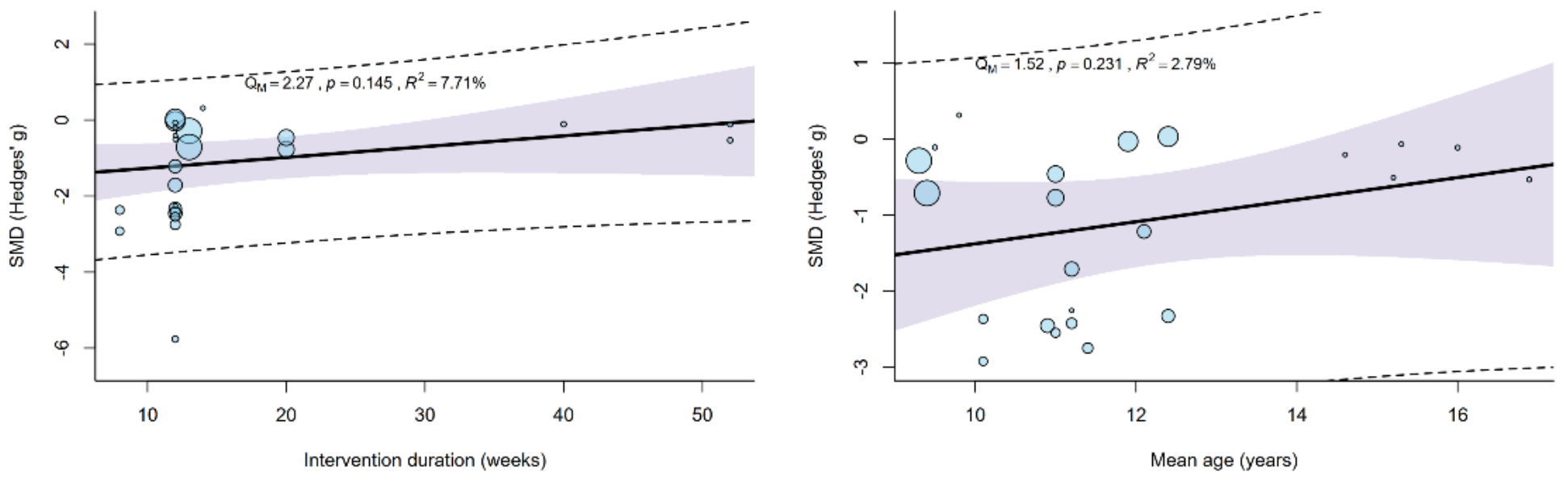

E

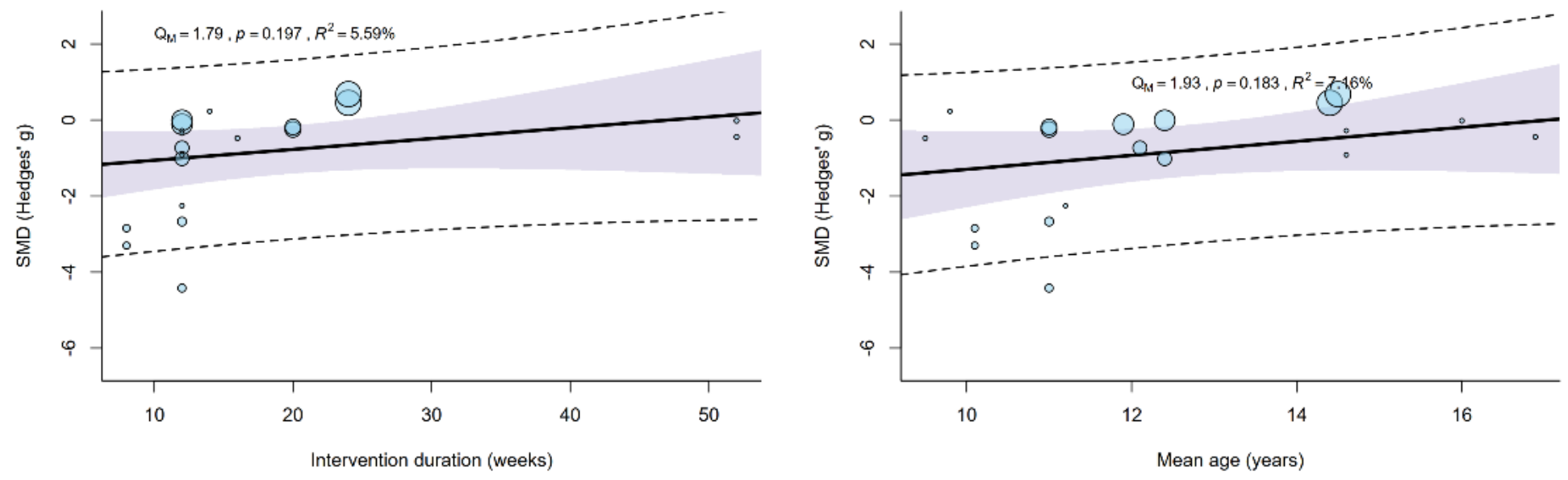

F

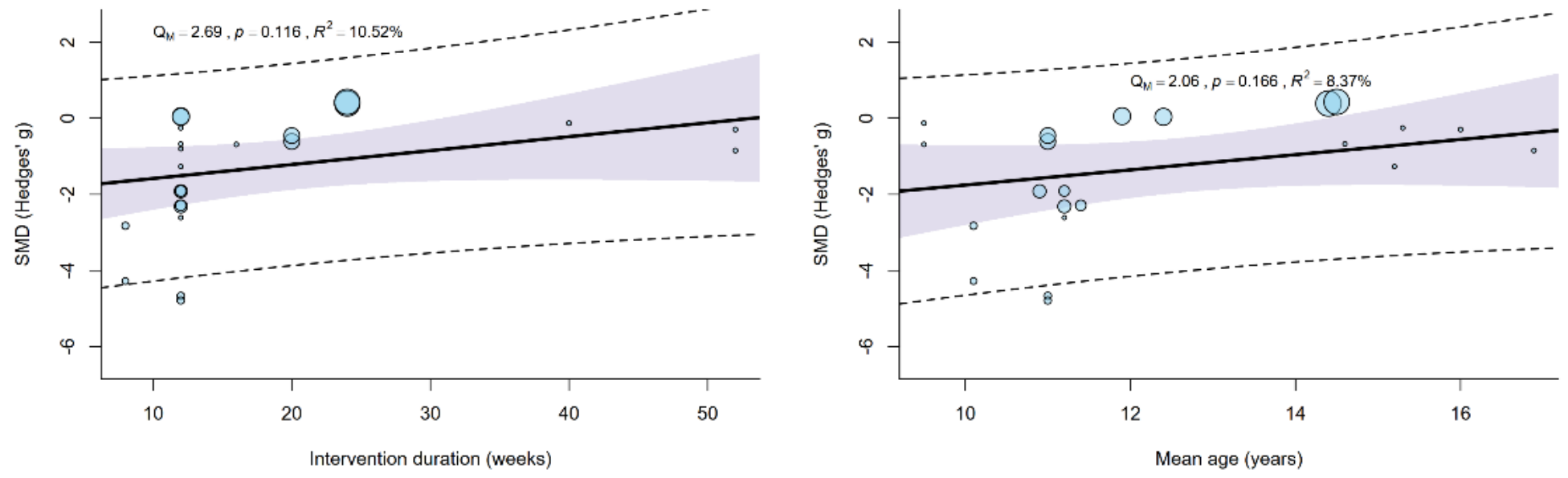

G

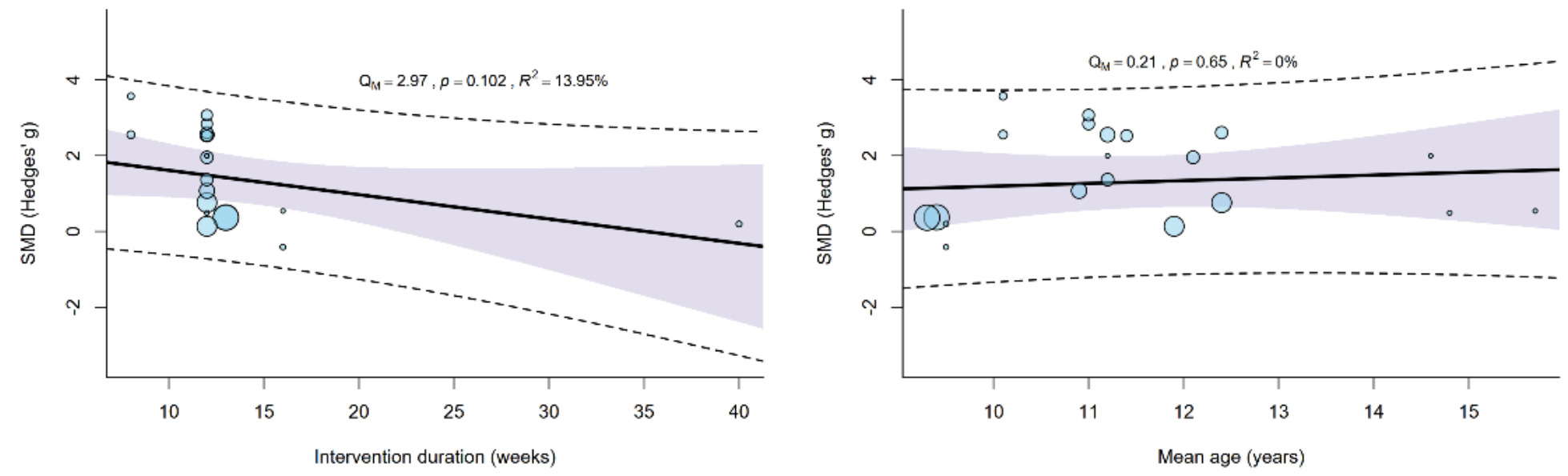

H

Supplementary Figure S4. Meta-regression plots of intervention duration and participants' mean age for the primary and secondary outcomes: A, visceral adipose tissue; B, subcutaneous adipose tissue; C, body fat percentage; D, waist circumference; E, body mass index; F, body weight; G, fat mass; H, maximal oxygen uptake.

Supplementary Figure S5.

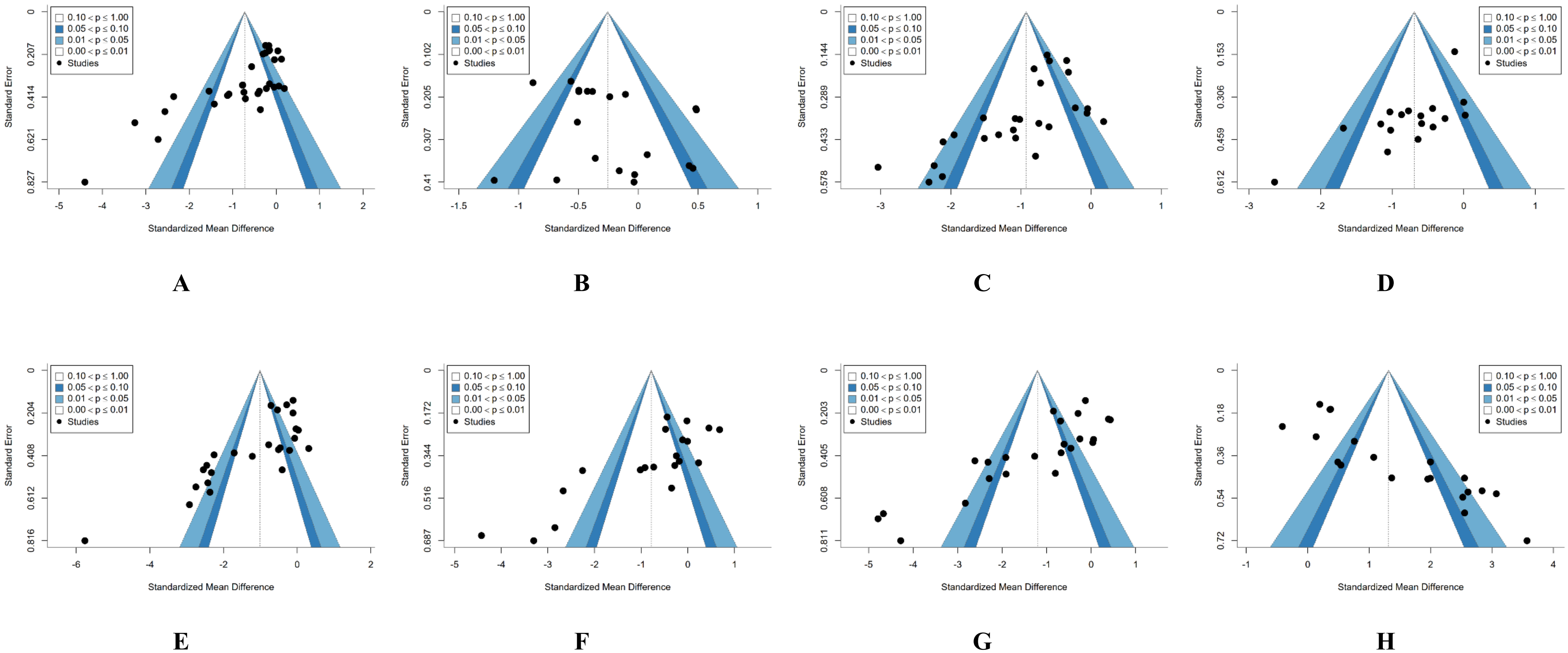

Supplementary Figure S5. Contour-enhanced funnel plots for the primary and secondary outcomes: A, visceral adipose tissue; B, subcutaneous adipose tissue; C, body fat percentage; D, waist circumference; E, body mass index; F, body weight; G, fat mass; H, maximal oxygen uptake.

Supplementary Figure S6.

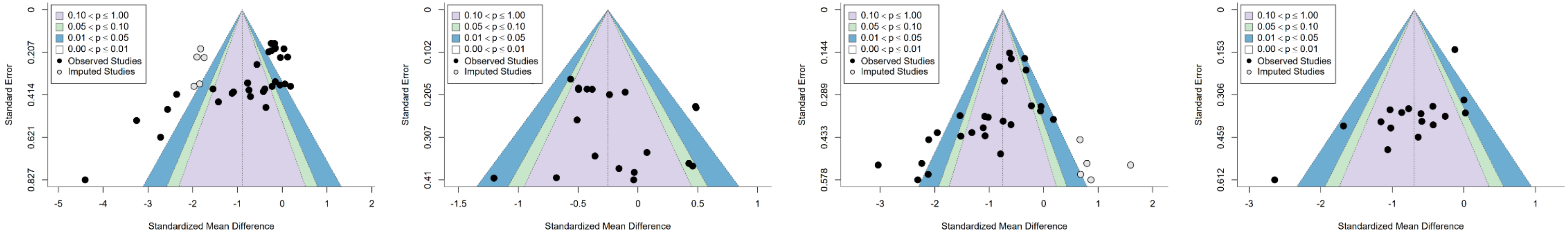

A B C D

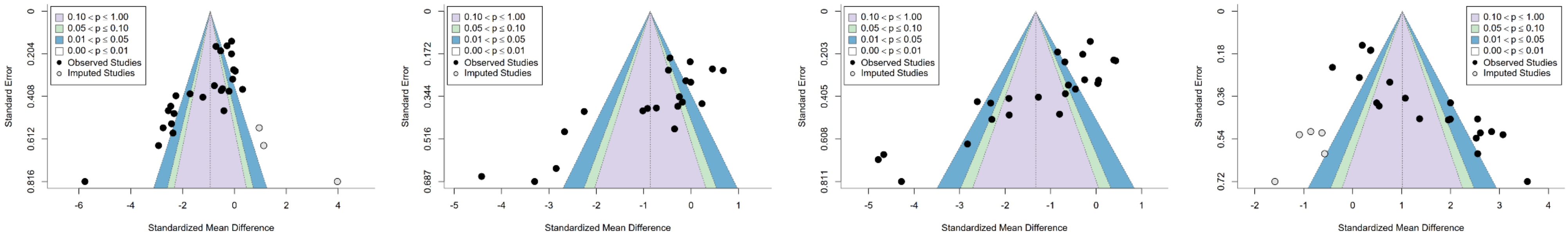

E F G H

Supplementary Figure S6. Trim-and-fill adjusted funnel plots for the primary and secondary outcomes: A, visceral adipose tissue; B, subcutaneous adipose tissue; C, body fat percentage; D, waist circumference; E, body mass index; F, body weight; G, fat mass; H, maximal oxygen uptake.

Supplementary Figure S7.

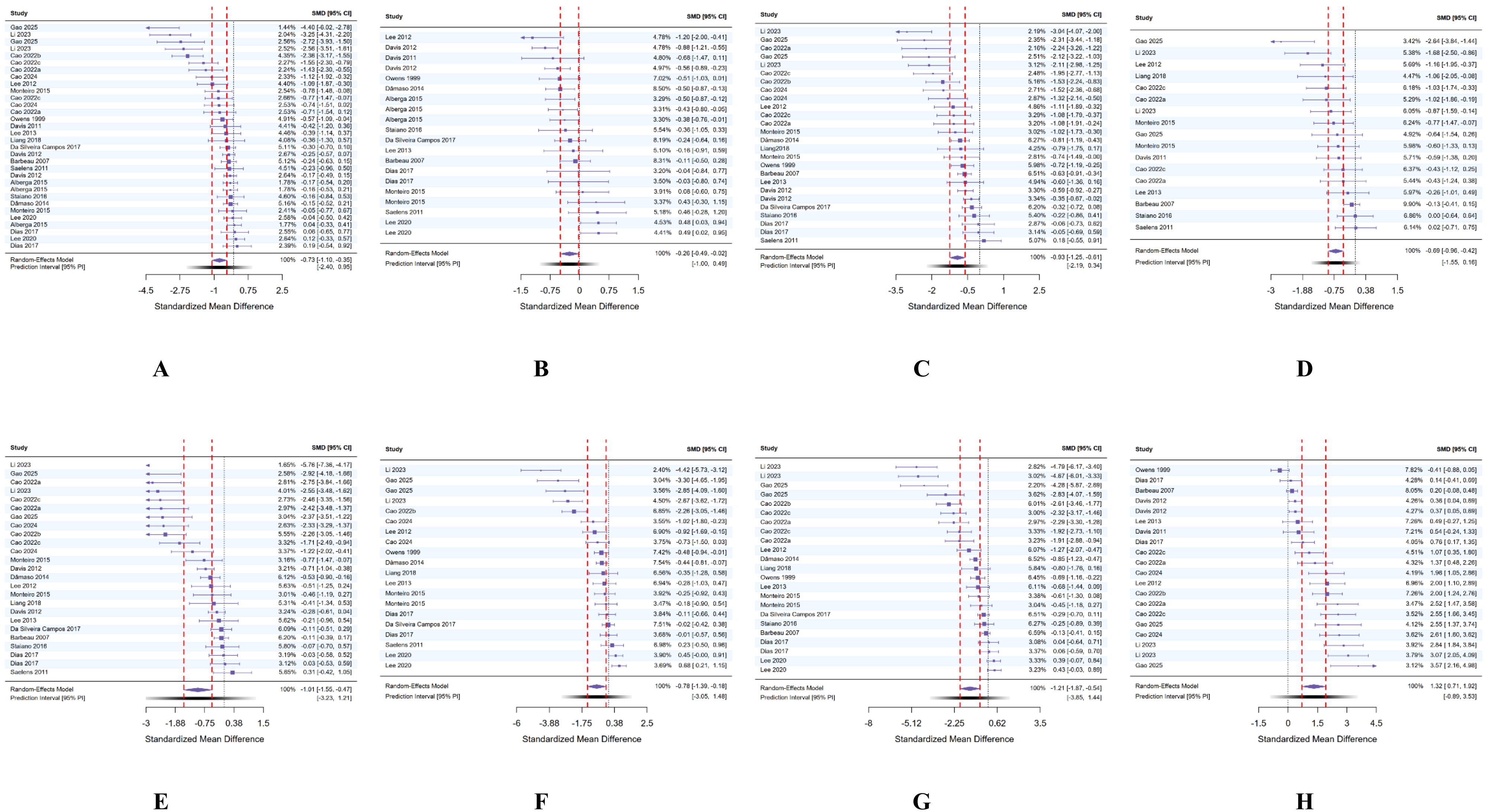

Supplementary Figure S7. Forest plots for outlier identification and comparison of pooled effects before and after outlier removal: A, visceral adipose tissue; B, subcutaneous adipose tissue; C, body fat percentage; D, waist circumference; E, body mass index; F, body weight; G, fat mass; H, maximal oxygen uptake.

Supplementary Figure S8.

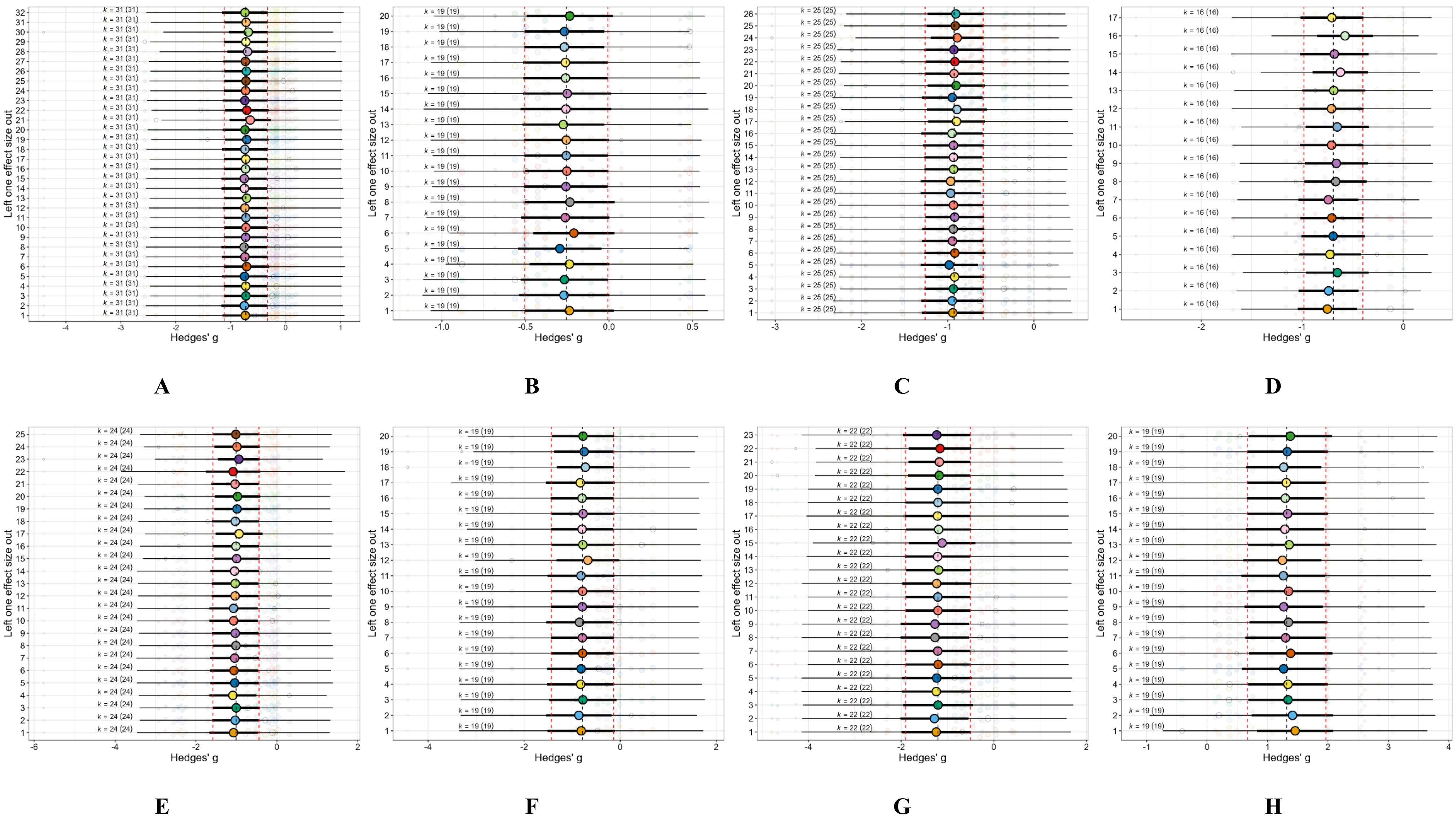

Supplementary Figure S8. Leave-one-effect-out sensitivity plots for the pooled effects of exercise interventions on the primary and secondary outcomes: A, visceral adipose tissue; B, subcutaneous adipose tissue; C, body fat percentage; D, waist circumference; E, body mass index; F, body weight; G, fat mass; H, maximal oxygen uptake.
